# The Solute Carrier Superfamily as Therapeutic Targets in Pancreatic Ductal Adenocarcinoma

**DOI:** 10.3390/genes16040463

**Published:** 2025-04-18

**Authors:** Sang Yeon Cho, Hyuk Soo Eun, Jaejeung Kim, Yun Dam Ko, Woo Sun Rou, Jong Seok Joo

**Affiliations:** 1 Graduate School of New Drug Discovery and Development, Chungnam National University, Daejeon 34134, Republic of Korea; nprc26@cnu.ac.kr; 2 CHOMEDICINE Inc., TIPS Town, Chungnam National University, Daejeon 34134, Republic of Korea; 3 Department of Internal Medicine, Chungnam National University School of Medicine, Daejeon 35015, Republic of Korea; 4 Department of Internal Medicine, Chungnam National University Hospital, Daejeon 35015, Republic of Korea; 5 Department of Computer Science and Engineering, Chungnam National University, Daejeon 34134, Republic of Korea; jjkim@cnu.ac.kr; 6 Seoul Teunteun Rehabilitation Clinic, Jeungpyeong-gun, Chungcheongbuk-do 27937, Republic of Korea; 7 Department of Internal Medicine, Chungnam National University Sejong Hospital, Sejong 30099, Republic of Korea

**Keywords:** SLC superfamily, pancreatic adenocarcinoma, precision oncology, SLC-based metabolic anti-cancer therapy

## Abstract

Background: Pancreatic ductal adenocarcinoma (PDAC), a challenging and malignant cancer, primarily originates from the exocrine cells of the pancreas. The superfamily of solute carrier (SLC) transporters, consisting of more than 450 proteins divided into 65 families, is integral to various cellular processes and represents a promising target in precision oncology. As therapeutic targets, SLC transporters are explored through an integrative analysis. Materials and Methods: The expression profiles of SLCs were systematically analyzed using mRNA data from The Cancer Genome Atlas (TCGA) and protein data from the Human Protein Atlas (HPA). Survival analysis was examined to evaluate the prognostic significance of SLC transporters for overall survival (OS) and disease-specific survival (DSS). Genetic alterations were examined using cBioPortal, while structural studies were performed with AlphaFold and AlphaMissense to predict functional impacts. Furthermore, Gene Set Enrichment Analysis (GSEA) was carried out to identify oncogenic pathways linked to SLC transporter expression. Results: SLC transporters were significantly upregulated in tumors relative to normal tissues. Higher expression levels of SLC39A10 (HR = 1.89, *p* = 0.0026), SLC22B5 (HR = 1.84, *p* = 0.0042), SLC55A2 (HR = 2.15, *p* = 0.00023), and SLC30A6 (HR = 1.90, *p* = 0.003) were strongly associated with unfavorable OS, highlighting their connection to poor prognosis in PDAC. GSEA highlighted that these four transporters are significantly involved in key oncogenic pathways, such as epithelial–mesenchymal transition (EMT), TNF-α signaling, and angiogenesis. Conclusions: The study identifies four SLCs as therapeutic targets in PDAC, highlighting their crucial role in essential metabolic pathways. These findings lay the groundwork for developing next-generation metabolic anti-cancer treatment to improve survival for PDAC patients.

## 1. Introduction

Pancreatic ductal adenocarcinoma (PDAC) poses considerable difficulties in both diagnosis and treatment, owing to its aggressive progression and dynamic molecular landscape [1]. Despite associations with risk factors like obesity, type 2 diabetes, tobacco use, and family history, PDAC often progresses undetected due to subtle early symptoms. As a result, its incidence and mortality continue to rise, making it one of the deadliest cancers [2]. This silent progression plays a major role in the alarmingly low five-year survival rate of about 10% and the increasing rates of cancer-related deaths [3]. Cancer therapy has rapidly evolved, incorporating targeted agents, immunotherapies, and personalized approaches [4]. While current therapies, such as KRAS-targeted therapies, immunotherapies, and poly(ADP-ribose) polymerase inhibitors, are being evaluated, ongoing research remains essential to discover novel therapeutic targets and improve treatment strategies for PDAC [5]. Particularly, the adaptive metabolic strategies of pancreatic cancer cells highlight an urgent need for the development of targeted metabolic therapies that could potentially disrupt these mechanisms [6].

The superfamily of solute carrier (SLC) transporters, comprising over 450 proteins organized into 65 families based on sequence similarity and transport function, plays a crucial role in transporting diverse substrates, including drugs, metabolites, and ions, through cell membranes [7]. As many SLCs are localized on the cellular surface, they can serve as metabolic targets for cancer, playing essential roles in regulating metabolic pathways and facilitating signal transduction [7,8]. SLCs, through genetic alterations, significantly drive uncontrolled cell proliferation and invasion while contributing to resistance against standard treatment [9,10]. Although specific SLC transporters have been linked to poor prognosis in other cancers, the clinical significance of the SLC transporter superfamily in PDAC remains largely unexamined [11]. Precision oncology aims to design treatments tailored to tumor genetics and characteristics, emphasizing novel therapeutic targets to improve prognosis [12]. Understanding the intricate roles of SLC transporters in various cancer types is crucial for discovering next-generation metabolic SLC targets in precision oncology [13].

Building on these insights, this study provides an integrative analysis of the SLC superfamily in PDAC, focusing on their expression patterns, genetic alterations, and prognostic significance. Leveraging multi-omics datasets, we systematically evaluated the differential expression and functional relevance of SLC transporters in PDAC. Functional enrichment analyses revealed critical metabolic and signaling pathways influenced by specific SLC transporters. These findings underscore the potential of SLC transporters as novel therapeutic targets, offering a foundation for future precision oncology strategies aimed at disrupting the metabolic adaptability of PDAC and improving patient outcomes.

## 2. Materials and Methods

### 2.1. Expression Data at the mRNA and Protein Levels

The dataset, including PDAC and normal samples RNA-seq expression data, was collected from UCSC Xena, uniformly processed by the TOIL process to be free of computational batch effects (https://xenabrowser.net/datapages/?cohort=GDC, accessed on 22 May 2024, TCGA Pancreatic Adenocarcinoma (PAAD)), encompassing data from The Cancer Genome Atlas (TCGA) and Genotype-Tissue Expression (GTEx) projects [14]. R (v.4.3.3, http://www.r-project.org) was utilized to process and analyze the raw data. Gene alterations and survival outcomes in TCGA-PAAD were examined through cBioPortal (http://www.cbioportal.org) [15]. The Human Protein Atlas (HPA) (http://www.proteinatlas.org) served as the source for all protein expression and immunohistochemistry (IHC) data. The R package “HPAanalyze” was employed to evaluate and interpret the IHC data from HPA for *SLC* genes, utilizing matched IHC images and associated information [16].

### 2.2. Survival Analysis

Kaplan–Meier plots were utilized to evaluate OS and DSS using mRNA and clinical data from TCGA-PAAD, extracted through UCSC Xena, to determine the independent prognostic value of SLC superfamily members. The optimal cutoff thresholds for mRNA expression were identified using Cutoff Finder [17]. Survival analysis was conducted for 446 *SLC* genes using three modes—outcome significance, distribution, and mean-based grouping—for both OS and DSS [17]. The optimal cutoff thresholds for mRNA expression were determined using the R script provided in the Cutoff Finder publication, rather than the web-based tool. Kaplan–Meier and Cox proportional hazards models were applied using standard survival analysis functions in R to assess survival differences [18]. A log-rank *p*-value of <0.05 was considered indicative of statistical significance.

### 2.3. Structural Analysis

The structural features of SLC proteins were investigated using AlphaFold and AlphaMissense. Protein structure predictions, leveraging deep learning techniques, were sourced from the AlphaFold database (https://alphafold.ebi.ac.uk/, accessed on 28 May 2024), which enabled the development of highly detailed 3D models for SLC transporters [19]. AlphaMissense was utilized to analyze the consequences of missense mutations on both the structure and function of these proteins. By combining structural data with mutation effect predictions, this tool offered an integrative analysis of how specific amino acid substitutions might alter the stability and functional activity of SLC transporters [20].

### 2.4. Gene Set Enrichment Analysis

GSEA was conducted using GSEA software v4.3.2 (https://www.gsea-msigdb.org/gsea/index.jsp, accessed on 28 May 2024) [20]. Enrichment analysis was performed using the Molecular Signatures Database (MSigDB, v2023.2.Hs), with 1000 permutations applied to assess statistical significance. Pathways with an FDR q-value < 0.25 and a nominal *p*-value < 0.05 were considered significantly enriched. The enrichment of hallmark pathways and Kyoto Encyclopedia of Genes and Genomes (KEGG) was investigated using GSEA, comparing groups with high and low levels of the target *SLC* gene. Significant pathways between these two groups were identified through this approach, providing an understanding of the biological processes.

### 2.5. Statistical Analysis

R software (version 4.3.3) was utilized for all statistical procedures, with access provided via http://www.r-project.org. For continuous variables, the *t*-test was applied, while categorical variables were examined using either the χ^2^ test or Fisher’s exact test, based on suitability. ANOVA was applied to evaluate statistical differences across multiple groups (≥3). To control for false discovery due to multiple testing, the Benjamini–Hochberg procedure was applied to the *p*-values obtained from *t*-tests comparing the expression of 446 *SLC* genes between cancerous and normal tissues [21]. A *p*-value threshold of <0.05 was considered indicative of statistical significance.

## 3. Results

### 3.1. mRNA Expression Profiles

**Figure 1:**
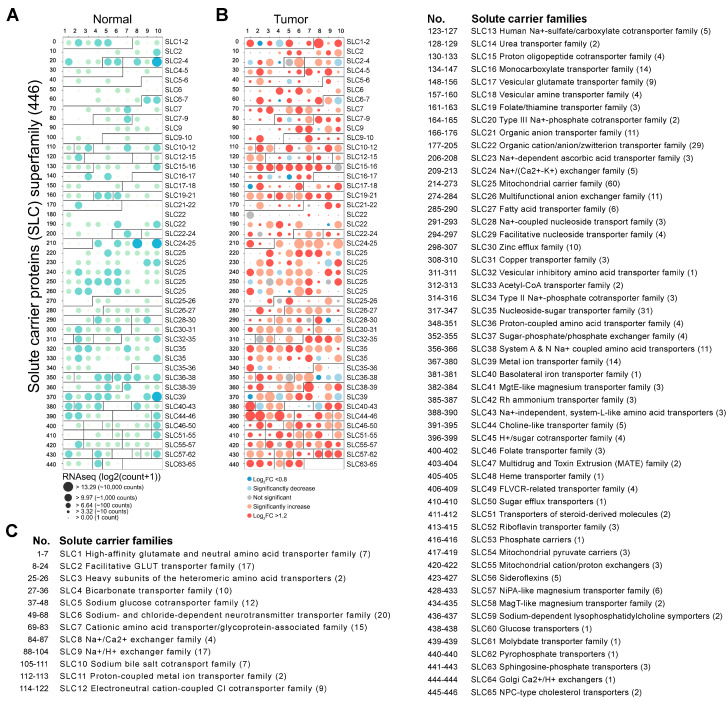
mRNA expression profiles. (**A**,**B**) The mRNA expression panel illustrates the expression changes of the 446 SLC superfamily genes, with normal tissues shown in (**A**) (green circles) and tumor tissues in (**B**) (red circles). Circle size represents mRNA expression levels, with larger circles indicating higher expression, while significant changes are emphasized by both color intensity and circle size. (**C**) The 65 SLC families are detailed in the list, providing the names of the analyzed families alongside their associated SLC numbers. The numbers on the left-hand side (0–400) correspond to the indexed order of the 446 *SLC* genes analyzed in this study. These numbers match the first column (‘No.’) in Supplementary Table S1, allowing for easy identification of specific genes within the dataset.

To explore the involvement of 65 SLC families in tumor progression, we conducted an extensive mRNA analysis encompassing all 446 SLCs across normal and tumor tissues. A detailed summary of all 446 *SLC* genes, categorized by their sequence and function, is provided in Supplementary Table S1 (http://slc.bioparadigms.org/). Supplementary Table S2 details the phenotype information for the 171 normal group samples (four from TCGA and 167 from GTEx) and the 179 PDAC tumor group samples. The findings, illustrated in Figure 1A,B, present the mRNA expression levels of *SLC* genes across normal and tumor tissues, highlighting significant differences in expression patterns between healthy and malignant tissues. Interestingly, 355 *SLC* genes exhibited marked upregulation, while 43 showed notable downregulation (Figure 1B). In normal pancreatic tissue, the genes with the most abundant expression were *SLC25A6*, *SLC39A14*, and *SLC4A4*, whereas in PDAC, *SLC25A6*, *SLC25A3*, and *SLC25A5* showed the highest levels of expression, in that order. Figure 1C lists the range of SLC members, each family name, and gene count, matched to the mRNA profile panel in Figure 1A,B, providing an accessible overview of the entire SLC superfamily. To further support the findings illustrated in Figure 1, Supplementary Table S3 provides the raw mRNA expression values for all 446 *SLC* genes across normal and tumor tissues.

### 3.2. Prognostic Survival Analysis of SLC Genes

**Figure 2:**
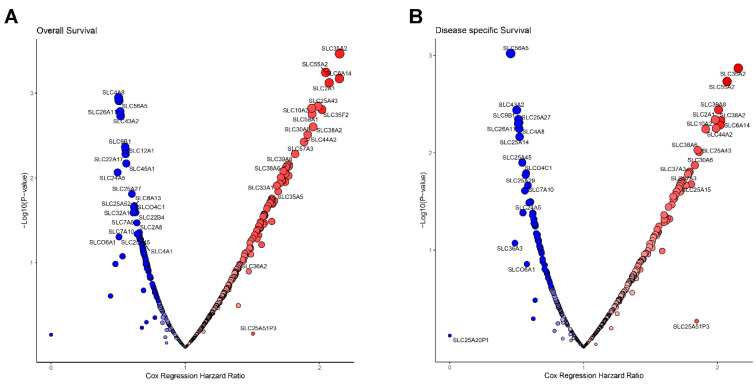
Survival analysis of SLCs. (**A**) Volcano plot illustrating the relationship between SLCs and OS. (**B**) Volcano plot depicting the correlation between SLC expression and DSS. On the plot, hazard ratios are depicted on the horizontal axis, whereas −log10(*p*-values) are displayed on the vertical axis. Blue circles denote SLCs linked to improved survival, whereas red circles represent those associated with poorer survival.

To evaluate the prognostic relevance of *SLC* genes, we conducted survival analysis for overall survival (OS) and disease-specific survival (DSS) across 446 *SLC* genes in PDAC. Figure 2A,B present the results, comparing hazard ratios and *p*-values for *SLC* genes to highlight their prognostic significance for OS and DSS. A total of 247 *SLC* genes were identified as statistically significant in the OS analysis, with 179 linked to poor prognosis and 68 to favorable prognosis (Figure 2A and Supplementary Table S4). Similarly, 226 *SLC* genes were found to be significant in the DSS analysis, including 156 associated with poor prognosis and 70 with favorable prognosis (Figure 2B and Supplementary Table S5). In both OS and DSS analyses, 200 *SLC* genes demonstrated statistically significant associations, including 147 linked to poor prognosis and 53 associated with favorable prognosis (Figure 2). To prioritize potential therapeutic targets, we identified *SLC* genes most strongly associated with poor prognosis by selecting 30 candidates that met the Cutoff Finder criteria, including thresholds for mean expression levels, distribution patterns, and outcome significance.

### 3.3. Protein Expression and Classification of SLC Transporters

**Figure 3:**
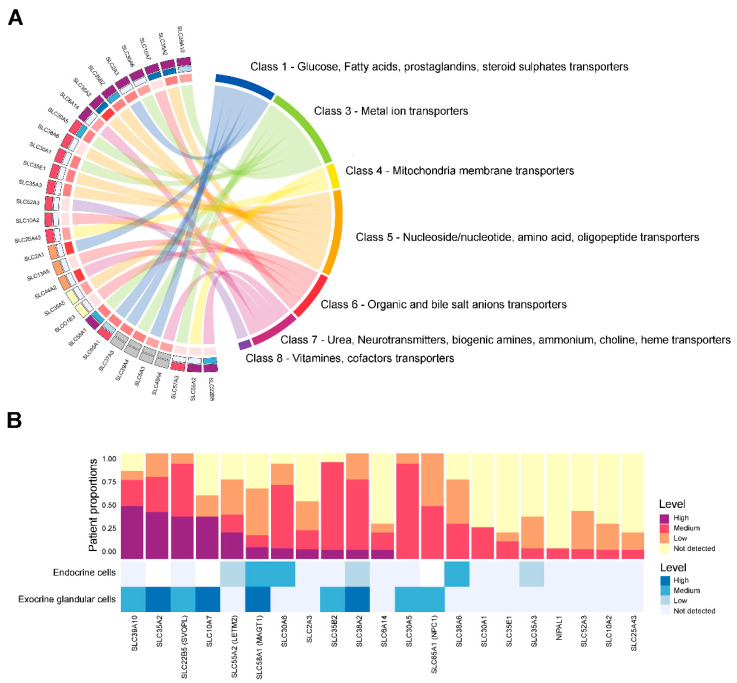
Protein expression and classification of SLC transporters in PDAC. (**A**) The Chord diagram displays the protein expression levels identified in tumor tissues (outer arc) and normal tissues (inner arc), with levels indicated by color: dark purple for high, red for medium, orange for low, yellow for not detected, and grey for unavailable. The selected 30 SLC transporters are listed in numerical order as follows: SLC2A1, SLC2A3, SLC5A3, SLC6A14, SLC10A2, SLC10A7, SLC13A5, SLCO1B3, SLC22B5, SLC25A43, SLC30A1, SLC30A5, SLC30A6, SLC35A2, SLC35A3, SLC35A5, SLC35B2, SLC35E1, SLC37A3, SLC38A2, SLC38A6, SLC39A4, SLC39A10, SLC44A2, SLC49A4, SLC52A3, SLC55A2, SLC57A3, SLC58A1, SLC65A1. (**B**) The y-axis represents the patient proportions, whereas the x-axis displays the analyzed SLC proteins. The color bars represent the detected levels of protein expression in each cell type. A bar chart illustrating the distribution of patients with selected SLC targets, with expression levels indicated by color: dark purple for high, red for medium, orange for low, and yellow for not detected. A bar chart depicting the protein expression levels of selected SLC targets in normal cells, with levels categorized by color: dark blue for high, blue for medium, light blue for low, and very light blue for not detected. The protein expression data were obtained from the Human Protein Atlas (HPA) immunohistochemistry (IHC) dataset. Expression Level Classification (Based on HPA Criteria): High: Strong staining in >75% of cells. Medium: Moderate staining in >75% of cells or strong staining in 25–75% of cells. Low: Weak staining in >75% of cells, moderate staining in 25–75% of cells, or strong staining in <25% of cells. Not detected: No staining observed or weak staining in <25% of cells.

To identify potential therapeutic targets for PDAC, we identified 30 *SLC* genes that exhibited consistent overexpression in tumor tissues relative to normal tissues and showed a strong correlation with unfavorable prognosis. Subsequently, the classification of the selected 30 SLC proteins was conducted to biologically categorize them, offering deeper insights into their characteristics in PDAC. The findings, depicted in Figure 3, classify 446 *SLC* genes into eight primary categories according to the solutes they facilitate transport for, alongside an analysis of their protein expression profiles. In Figure 3A, the 30 selected *SLC* genes are grouped into seven distinct categories based on the solutes they transport. Class 1 consists of transporters for glucose, fatty acids, prostaglandins, and steroid sulfates. Class 3 includes metal ion transporters, while Class 4 features mitochondrial membrane transporters. Class 5 encompasses nucleoside/nucleotide, amino acid, and oligopeptide transporters. Class 6 represents transporters for organic and bile salt anions. Class 7 includes those responsible for the transport of urea, neurotransmitters, biogenic amines, ammonium, choline, and heme. Finally, Class 8 is composed of transporters for vitamins and cofactors. However, no *SLC* genes from Class 2, which includes transporters for inorganic cations and anions, were detected in this analysis. Among these, protein levels for four *SLC* genes were unavailable, and five *SLC* genes exhibited undetectable or low protein expression. The classification of *SLC* genes was based on their functional ontology and substrate specificity, as detailed in Supplementary Table S6.

To gain a deeper understanding of the expression profiles, we evaluated the proportion of patients with protein expression of 21 SLC proteins in tumor tissues as well as in normal endocrine and exocrine glandular cells, as shown in Figure 3B. A significant proportion of patients showed elevated expression levels of the following proteins in PDAC: SLC39A10, SLC22B5, SLC55A2, SLC30A6, SLC2A3, SLC35B2, and SLC6A14. Despite the significant mRNA upregulation observed in SLCs, the corresponding 14 SLC proteins did not exhibit a notably elevated protein expression compared to normal exocrine glandular cells.

### 3.4. Integrative Analysis of the Four Selected SLC Targets in PDAC

**Figure 4:**
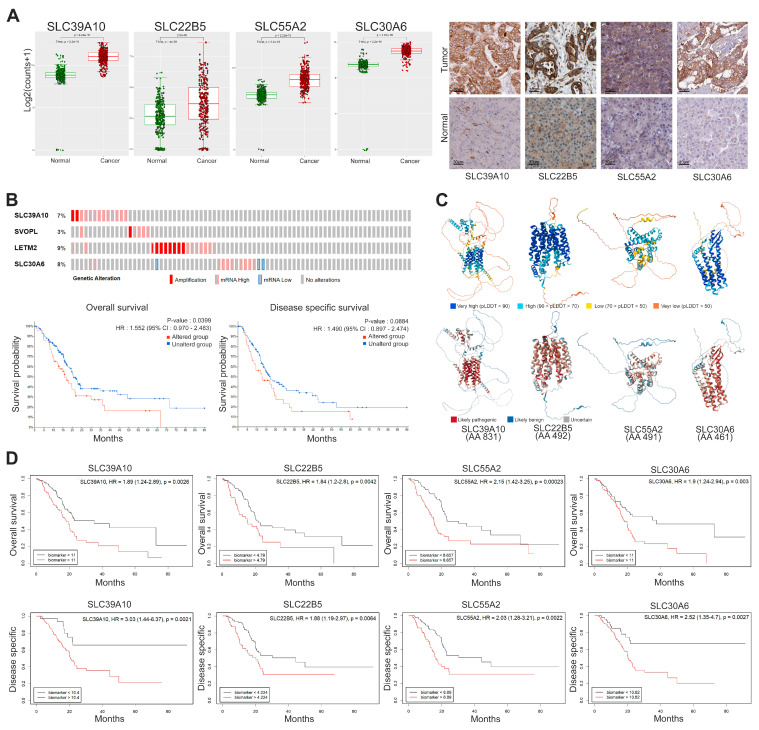
Integrative analysis of expression profiles, genetic variations, protein structural features, and survival analysis of selected SLC targets. (**A**) Box plots illustrating the mRNA levels of *SLC39A10*, *SLC22B5*, *SLC55A2*, and *SLC30A6*, comparing normal tissues (green) to cancer tissues (red). Elevated expression levels are confirmed by immunohistochemistry staining. The scale bars represent 50 μm. (**B**) Genetic alterations of selected SLC targets are shown alongside survival plots, illustrating OS and DSS for patients. Statistical significance was assessed using the log-rank test. (**C**) Using AlphaFold2, the 3D structures of SLC transporters were predicted and visually represented with color coding based on predicted local distance difference test scores: blue for very high confidence above 90, light blue for high confidence between 70 and 90, yellow for low confidence between 50 and 70, and red for very low confidence below 50, providing a detailed visualization of structural reliability. AlphaMissense was employed to categorize specific amino acid mutations as likely pathogenic, benign, or uncertain, utilizing protein structural context and evolutionary constraints for classification. (**D**) Survival curves illustrate the association between elevated (red) and reduced (black) expression levels of *SLC* genes with OS and DSS, including hazard ratios and *p*-values to highlight their prognostic relevance. Data sources: (**A**) mRNA expression data were obtained from TCGA and GTEx, and protein expression data from the Human Protein Atlas (HPA) IHC dataset. (**B**) Genetic alterations were sourced from cBioPortal (TCGA-PAAD cohort). (**C**) Protein structural predictions were retrieved from AlphaFold, with mutation impact classification performed using AlphaMissense. (**D**) Survival analysis was conducted using Cutoff Finder, with optimal cutoff values applied for OS and DSS.

For an integrative analysis, we prioritized *SLC* genes that exhibited elevated expression at both the mRNA and protein levels, focusing on those with the highest proportion of patients demonstrating high protein expression. Specifically, our analysis focused on SLC39A10, SLC22B5, SLC55A2, and SLC30A6 to evaluate their expression profiles, genetic alterations, protein structure, and prognostic significance. Figure 4A illustrates the significantly elevated mRNA expression of *SLC39A10*, *SLC22B5*, *SLC55A2*, and *SLC30A6* in cancer tissues compared to normal tissues, while the immunohistochemistry data in the right panel further confirm the increased protein levels of these SLCs in cancer tissues.

Figure 4B highlights the rates of genetic alterations in the selected SLC targets, showing notable gene amplification and elevated mRNA expression levels. Genetic alterations in these SLC targets, as demonstrated by survival analyses, are significantly associated with reduced OS, with a hazard ratio of 1.552, a 95% confidence interval of 0.970 to 2.483, and a *p*-value of 0.0399. Additionally, they show a trend toward poorer DSS, with a hazard ratio of 1.490, a 95% confidence interval of 0.897 to 2.474, and a *p*-value of 0.0884. Figure 4C presents the predicted 3D structures of the selected SLC proteins, modeled using AlphaFold2, providing information on their structural stability and functional domains, with scores indicating the confidence levels of the predictions. Among the proteins, SLC39A10 (831 amino acids) and SLC55A2 (491 amino acids) show challenges in structural prediction due to significantly low-confidence regions, while SLC22B5 (492 amino acids) and SLC30A6 (461 amino acids) demonstrate relatively higher confidence in their predicted structures, particularly in functional regions. AlphaFold2 predictions for certain regions of SLC39A10 and SLC55A2 reported low confidence scores, indicating potential challenges in modeling these domains due to their structural complexity or limited representation in the available training datasets. Despite these limitations, the combined analysis offers valuable insights into the structural and functional significance of these SLC targets and their potential pathogenic mutation sites in each SLC predicted 3D structure.

Figure 4D depicts the prognostic impact of SLC expression levels, highlighting significant variations in OS and DSS outcomes between groups with high and low expression levels. High expression of *SLC39A10* is strongly associated with poorer OS, with a hazard ratio of 1.89, a confidence interval from 1.24 to 2.89, and a *p*-value of 0.0026, as well as worse DSS, with a hazard ratio of 3.03, a confidence interval from 1.44 to 6.37, and a *p*-value of 0.0021. Similarly, elevated levels of *SLC22B5* are linked to poorer outcomes, including OS with a hazard ratio of 1.84, a confidence interval from 1.20 to 2.80, and a *p*-value of 0.0042, and DSS with a hazard ratio of 1.88, a confidence interval from 1.19 to 2.97, and a *p*-value of 0.0064. Overexpression of *SLC55A2* is also correlated with worse OS, with a hazard ratio of 2.15, a confidence interval from 1.42 to 3.25, and a *p*-value of 0.00023, as well as DSS, with a hazard ratio of 2.03, a confidence interval from 1.28 to 3.21, and a *p*-value of 0.0022. Lastly, the high expression of *SLC30A6* is significantly linked to poorer OS, with a hazard ratio of 1.90, a confidence interval from 1.24 to 2.94, and a *p*-value of 0.003, and DSS, with a hazard ratio of 2.52, a confidence interval from 1.35 to 4.70, and a *p*-value of 0.0027.

### 3.5. Gene Set Enrichment Analysis for Four Selected SLCs in PDAC

**Figure 5:**
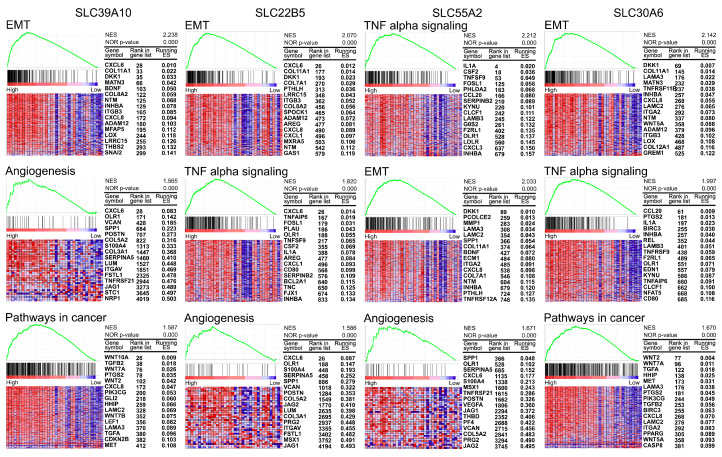
Gene Set Enrichment Analysis in PDAC. Significantly enriched pathways in cancers with elevated mRNA levels of *SLC39A10*, *SLC22B5*, *SLC55A2*, and *SLC30A6* are illustrated through heatmaps and enrichment plots. The most significant pathways, along with their corresponding NES and *p*-values, are highlighted in each panel. The expression of key genes within each pathway are illustrated in the heatmaps, with samples arranged according to the mRNA expression of the corresponding *SLC* gene. For *SLC39A10*, enrichment was observed in the EMT pathway (q < 0.0001), angiogenesis pathway (q = 0.0020), and pathways in cancer (q = 0.0301). For *SLC22B5*, significant enrichment was found in the EMT pathway (q < 0.0001), TNF-α signaling (q = 0.00049), and angiogenesis pathway (q = 0.0029). For *SLC55A2*, enrichment was detected in TNF-α signaling (q < 0.0001), EMT pathway (q < 0.0001), and angiogenesis pathway (q = 0.0023). For *SLC30A6*, significant pathways included EMT (q < 0.0001), TNF-α signaling (q < 0.0001), and pathways in cancer (q < 0.0001).

To investigate the mechanisms linked to the four SLC targets, GSEA was conducted to identify enriched pathways in tumors with elevated mRNA levels of *SLC39A10*, *SLC22B5*, *SLC55A2*, and *SLC30A6*. Figure 5 highlights significant enrichment pathways across multiple oncogenic pathways. For *SLC39A10*, significant enrichment was observed in the EMT pathway with a normalized enrichment score (NES) of 2.238 and a *p*-value < 0.0001, the angiogenesis pathway with a NES of 1.565 and a *p*-value < 0.0001, and pathways in cancer with a NES of 1.587 and a *p*-value < 0.0001. For *SLC22B5*, enrichment was noted in the EMT pathway with a NES of 2.070 and a *p*-value < 0.0001, TNF-α signaling with a NES of 1.820 and a *p*-value < 0.0001, and the angiogenesis pathway with a NES of 1.586 and a *p*-value < 0.0001. *SLC55A2* exhibited significant enrichment in TNF-α signaling with a NES of 2.212 and a *p*-value < 0.0001, the EMT pathway with a NES of 2.033 and a *p*-value < 0.0001, and the angiogenesis pathway with a NES of 1.671 and a *p*-value < 0.0001. Finally, *SLC30A6* showed enrichment in several pathways, including the EMT transition pathway with a NES of 2.142 and a *p*-value < 0.0001, TNF-α signaling with a NES of 1.997 and a *p*-value < 0.0001, and pathways in cancer with a NES of 1.670 and a *p*-value < 0.0001.

## 4. Discussion

Precision medicine employs comprehensive genomic and molecular profiling of tumor biopsies to enhance cancer diagnosis and facilitate the development of targeted therapies customized to the unique features of each tumor [22]. The progression from chemotherapy through targeted and immunotherapies has led to the field of metabolic anticancer therapies [13]. An important focus within the precision oncology approach is the superfamily of solute carrier transporters (SLC), comprising over 450 proteins divided into 65 families [7]. SLC transporters are essential for facilitating the movement of ions, metabolites, and pharmaceutical compounds across cell membranes. In cancer, these transporters are particularly important as they fulfill the increased metabolic needs of tumor cells, facilitating the absorption of key nutrients like amino acids, nucleotides, and other vital metabolites [8]. Importantly, numerous SLC transporters are located on the cell surface, positioning them as highly druggable targets for cancer therapy [23]. By leveraging SLC transporters, metabolic anticancer therapies can be tailored to the specific metabolic characteristics of different cancer types, enhancing treatment effectiveness. These insights are crucial for identifying novel therapeutic SLC targets and developing SLC-based metabolic cancer therapies. Thus, continued investigation of SLC transporter roles in cancer is crucial for driving advancements in precision oncology and improving clinical outcomes for PDAC patients.

This study highlights the significant involvement of SLC transporters, particularly SLC39A10, SLC22B5, SLC55A2, and SLC30A6, in the underlying mechanisms of PDAC, emphasizing their strong potential as effective therapeutic targets. By analyzing mRNA expression data from The Cancer Genome Atlas (TCGA) and protein expression data from the Human Protein Atlas (HPA), our study identified significant upregulation of these *SLC* genes in PDAC, correlating with a poorer prognosis. GSEA identified significant enrichment of multiple oncogenic pathways, such as EMT, angiogenesis, and the TNF-α pathway, in tumors exhibiting high expression of these SLCs. These pathways are widely acknowledged as critical regulators of tumor growth, metastatic dissemination, and alterations in the tumor microenvironment. Enhanced invasive and migratory abilities, driven by the EMT pathway, are key contributors to the promotion of metastasis in cancer cells [24]. The role of these SLCs in sustaining the aggressive phenotype of PDAC is further emphasized by their association with enhanced angiogenesis and TNF-α signaling pathways [25,26].

Among the 446 *SLC* genes, *SLC25A6*, *SLC39A14*, and *SLC4A4* exhibited the greatest expression levels in normal pancreatic tissues, in that order. The SLC25 family, referred to as the mitochondrial carrier group, encompasses various transporters that facilitate metabolite transport across the inner membrane of mitochondria [27]. As an exchanger, SLC25A6, alternatively referred to as adenine nucleotide translocase-3 (ANT3), is essential for sustaining the cellular energy equilibrium by transporting ADP into the mitochondria and ATP out [28]. SLC39A14, part of the ZIP transporter family, plays a critical role in the cellular uptake of zinc and other divalent ions [29]. This transporter is essential for maintaining zinc homeostasis within the cells, influencing various biological processes including enzyme function, protein folding, and cellular signaling pathways [30]. Its expression in the pancreas suggests a significant role in managing the ion balance necessary for pancreatic functions, with research on mouse models lacking SLC39A14 in pancreatic β cells showing that its absence leads to hyperinsulinemia, highlighting its crucial role in insulin metabolism and glucose homeostasis [31]. SLC4A4, categorized within the bicarbonate transporter family, primarily facilitates the exchange of bicarbonate and sodium ions across cellular membranes [32]. This action is vital for regulating intracellular pH and maintaining acid-base fluids, which are crucial for neutralizing stomach acid and providing an optimal intestinal environment for enzymatic digestion [33].

The strong correlation between a poorer prognosis in PDAC and the persistent overexpression of *SLC39A10*, *SLC22B5*, *SLC55A2*, and *SLC30A6* at both the mRNA and protein levels in malignant tissues was evident in our study. The SLC39A10 transporter, a member of the ZIP transporter family, is crucial for the cellular import of zinc, while the SLC30A6 transporter, from the SLC30 (ZnT) family, specifically regulates zinc distribution within the Golgi apparatus and mitochondria [29,34,35,36]. Zinc (Zn), present in the amount of about 2 g in the human body, is a vital trace element that is more extensively involved in the eukaryotic proteome than iron, associating with 9% compared to iron’s 1% [37,38]. It is essential for over 300 enzymatic reactions and the structural integrity of approximately 2000 transcription factors via Zn finger domains [39]. Zinc plays a crucial role in the pancreas and in pancreatic cancer due to its fundamental involvement in cellular growth and differentiation, acting as a key regulator of numerous enzymes and protein activities [30,40]. In the pancreas, zinc is crucial for the production, storage, and release of insulin by β cells, maintaining its structure through binding with insulin molecules [41]. However, ZIP10 is primarily localized at the plasma membrane in pancreatic α cells, while ZnT6 is found in the Golgi apparatus, reflecting distinct subcellular functions for zinc uptake and distribution [42]. Zinc also serves a protective role against oxidative stress by participating in antioxidant defense mechanisms, minimizing cellular damage [43]. In pancreatic cancer, alterations in zinc metabolism potentially facilitate the use of vital minerals necessary for proliferation and survival. Targeting zinc transporters offers a promising therapeutic approach, as regulating zinc transport can impact cancer cell metabolism and proliferation, thereby inhibiting pancreatic cancer progression and enhancing the efficacy of treatments [44]. A significant upregulation of *SLC39A10* and *SLC30A6* at both the mRNA and protein levels in PDAC was observed in our study, suggesting a movement of zinc from the extracellular space and plasma membrane to the Golgi apparatus, where ZIP10 and ZnT6 are, respectively, localized. This process enhances zinc transport to the Golgi, where Zn^2+^ is essential for the activity of glycosylation enzymes such as α-mannosidase II (GMII), which are critical for N-glycosylation, a key post-translational modification [45,46]. The strong association between elevated expression and poorer prognosis suggests that SLC39A10 and SLC30A6 could serve as valuable therapeutic targets in PDAC through their role in regulating zinc metabolism. Additionally, GSEA highlighted the significant connection of *SLC39A10* and *SLC30A6* to EMT and other cancer pathways, further supporting their potential as therapeutic targets in PDAC by influencing essential mechanisms of tumor progression.

The SLC22 family comprises a diverse group of transporters, encompassing organic cation transporters (OCTs), organic anion transporters (OATs), and zwitterion/cation transporters (OCTNs) [47]. The *SLC22B5* gene encodes SVOPL (SVOP Like), which is a member of the SLC22 transporter family. *SVOPL* is a paralog of the *SVOP* gene, which is known to encode the synaptic vesicle 2-related protein, suggesting a possible role in synaptic function that has yet to be fully explored [48]. The SLC22 family, particularly OATs and OCTs, plays a crucial role in shaping pharmacokinetics and drug disposition [48]. In this analysis, a notable overexpression of *SLC22B5* at both the mRNA and protein levels was observed in PDAC. This overexpression was significantly correlated with a worse clinical prognosis, underscoring the potential of SLC22B5 as a therapeutic target in PDAC. Moreover, *SLC22B5* is also significantly related to EMT, TNF α signaling, and angiogenesis in GSEA. Previous studies have not yet investigated the relationship between SLC22B5 and critical biological processes, including EMT, TNF-α signaling, or angiogenesis, in pancreatic cancer. As far as we are aware, this study is the first to report the implications of SLC22B5 in PDAC, suggesting new potential strategies for therapeutic intervention. The *SLC55A2* gene encodes *LETM2*, a part of the SLC55 family recognized for its function as mitochondrial cation/proton exchangers. LETM2 is primarily involved in the regulation of ion balance within mitochondria, crucial for maintaining mitochondrial membrane potential and overall cellular energy homeostasis [49]. Our research demonstrated significant overexpression of *SLC55A2* at both the mRNA and protein levels in PDAC, which was closely associated with worse clinical outcomes. GSEA revealed strong links between *SLC55A2* expression and critical pathways, such as TNF-α signaling, EMT, and angiogenesis. These findings highlight the potential of SLC55A2 as a promising therapeutic target in PDAC. It was also reported that overexpression of LETM2 impacts mitochondrial function, linking it to the advancement of gastric cancer [50]. This highlights the critical importance of mitochondrial dynamics in cancer prognosis and treatment strategies, providing valuable understanding for our observations of SLC55A2 in PDAC.

The study identifies four SLC transporters as promising therapeutic targets in PDAC, underscoring their role in metabolic adaptation and tumor progression. Notably, these transporters have also been implicated in other malignancies. For example, SLC39A10 (ZIP10) is overexpressed in gastric, hepatic, and breast cancers, where it enhances tumor growth and metastasis via Zn^2+^-dependent signaling pathways such as PI3K/AKT and MAPK/ERK [51,52,53]. Similarly, SLC30A6 (ZnT6) is significantly upregulated in pancreatic adenocarcinoma and contributes to poor prognosis by promoting cancer cell proliferation via ERK1/2, p38 MAPK, and NF-κB signaling pathways [54]. SLC55A2 (LETM2) promotes tumor growth and metastasis in pancreatic cancer via the PI3K-AKT pathway, with limited evidence in other cancer types [55]. Although research on SVOPL is limited, it may play a role in epigenetic dysregulation via allelic switching in colorectal cancer, while in breast cancer, higher expression appears linked to better treatment response and prognosis [56,57]. These observations are consistent with growing interest in targeting SLC transporters to disrupt cancer metabolism. Recent advances in cancer metabolism have highlighted the therapeutic potential of targeting solute carrier (SLC) transporters to overcome nutrient dependency and metabolic resistance. In PDAC, several studies have demonstrated promising effects of inhibiting specific SLCs. For example, the blockade of SLC1A5 (ASCT2) impaired glutamine uptake and suppressed tumor growth by disrupting energy production and inducing apoptosis [58]. Similarly, targeting SLC7A11 (xCT) with sulfasalazine or siRNA-loaded nanoparticles inhibited tumor progression and remodeled the tumor microenvironment by limiting cystine uptake and redox buffering [59]. In addition, knockdown of SLC5A3, a myo-inositol transporter, sensitized gemcitabine-resistant PDAC cells to apoptosis through mitochondrial dysfunction and mitophagy [60]. Beyond PDAC, clinical and preclinical studies in other cancers have demonstrated the efficacy of targeting SLC transporters such as SLC7A5 (LAT1) [61], SLC16A1 (MCT1) [62], and SLC6A14 [63] in suppressing tumor growth and modulating therapeutic resistance.

Despite the recognized potential of SLC transporters as critical therapeutic targets in PDAC, significant challenges hinder the full realization of their capabilities. These challenges stem from the vast diversity and heterogeneity of the SLC superfamily, comprising over 450 members across more than 65 families, each with unique substrate specificities and transport mechanisms. Additionally, the complexity of developing precise assay technologies to assess transporter activity and the technical difficulties in achieving high-quality protein crystallization for structural studies further limit progress in this field. While this study provides valuable insights, reliance on publicly available databases introduces potential biases related to variations in sample collection, processing, and data annotation [64]. Experimental validation is necessary to confirm the functional roles of the identified SLC targets and their therapeutic potential in PDAC. Future research must prioritize investigating the mechanistic roles of these transporters using both in vitro and in vivo models, including patient-derived xenograft (PDX) models, addressing these methodological challenges to advance their application in precision medicine.

## 5. Conclusions

By thoroughly characterizing the SLC transporter superfamily in PDAC, our research highlights their relevance as potential therapeutic candidates. SLC39A10, SLC22B5, SLC55A2, and SLC30A6 were identified as key mediators in PDAC pathogenesis through the integration of multi-omics data and advanced analytical pipelines. Among the identified transporters, SLC39A10 and SLC30A6 mediate PDAC-specific zinc metabolic reprogramming, SLC22B5 represents a novel finding in pancreatic cancer, and SLC55A2 is associated with mitochondrial dysfunction and aggressive tumor behavior. Targeting these transporters may impair vital metabolic functions in cancer cells, offering a promising avenue for treating this aggressive form of cancer. This study lays the groundwork for SLC-based metabolic therapies in precision oncology, with the aim of enhancing clinical outcomes in PDAC patients.

## Data Availability

Publicly available datasets were analyzed in this study. These data can be found here: The Cancer Genome Atlas (TCGA) and Genotype-Tissue Expression (GTEx) [Link: https://xenabrowser.net/datapages/?cohort=GDC TCGA Pancreatic Adenocarcinoma (PAAD) (accessed on 3 August 2024)].

## Supplementary Materials

The following supporting information can be downloaded at: https://www.mdpi.com/article/10.3390/genes16040463/s1, Table S1: The summary of 446 SLC Transporters; Table S2: The phenotype information of normal and PDAC samples; Table S3: mRNA expression profile; Table S4: Overall survival analysis by Cutoff finder; Table S5: Disease specific survival analysis by Cutoff finder; Table S6: The classification of SLC superfamily.
