# Supplementary material for: The Solute Carrier Superfamily as Therapeutic Targets in Pancreatic Ductal Adenocarcinoma
Source: Genes (Basel). 2025 Apr 18;16(4):463. doi: 10.3390/genes16040463 (PMC12027052; doi:10.3390/genes16040463)
Supplement: Supplementary file 1 [file genes-16-00463-s001.zip › Table S1.pdf]

Table S1. The summary of 446 SLC Transporters

No.	SLC name	Ensembl ID	Protein name	Aliases	Transport type*	Substrates
1	SLC1A1	ENSG00000106688.11	EAAC1, EAAT3	System X-AG	C / Na+, H+, K+	L-Glu, D/L-Asp
2	SLC1A2	ENSG00000110436.11	GLT-1, EAAT2	System X-AG	C / Na+, H+, K+	L-Glu, D/L-Asp
3	SLC1A3	ENSG00000079215.13	GLAST, EAAT1	System X-AG	C / Na+, H+, K+	L-Glu, D/L-Asp
4	SLC1A4	ENSG00000115902.10	ASCT1, SATT	System ASC	C / Na+, E / amino acids	L-Ala, L-Ser, L-Cys, L-Thr
5	SLC1A5	ENSG00000105281.12	ASCT2, AAAT	System ASC	C / Na+, E / amino acids	L-Ala, L-Ser, L-Cys, L-Thr, L-Gln, L-Asn
6	SLC1A6	ENSG00000105143.12	EAAT4	System X-AG	C / Na+, H+, K+	L-Glu, D/L-Asp
7	SLC1A7	ENSG00000162383.11	EAAT5	System X-AG	C / Na+, H+, K+	L-Glu, D/L-Asp
8	SLC2A1	ENSG00000117394.19	GLUT1		F	glucose, galactose, mannose, glucosamine
9	SLC2A2	ENSG00000163581.13	GLUT2		F	glucose, galactose, fructose, mannose, glucosamine
10	SLC2A3	ENSG00000059804.15	GLUT3		F	glucose, galactose, mannose, xylose
11	SLC2A3P1	ENSG00000253861.1	pseudogene	GLUT3 pseudogene 1		
12	SLC2A3P2	ENSG00000185031.6	pseudogene	GLUT3 pseudogene 2		
13	SLC2A3P4	ENSG00000254088.1	pseudogene	GLUT3 pseudogene 4		
14	SLC2A4	ENSG00000181856.14	GLUT4		F	glucose, glucosamine
15	SLC2A5	ENSG00000142583.17	GLUT5		F	fructose
16	SLC2A6	ENSG00000160326.13	GLUT6	GLUT9	F	glucose
17	SLC2A7	ENSG00000197241.3	GLUT7		F	glucose, fructose
18	SLC2A8	ENSG00000136856.17	GLUT8	GLUTX1	F	glucose, fructose, galactose
19	SLC2A9	ENSG00000109667.11	GLUT9	GLUTX, URATv1		urate (glucose, fructose)
20	SLC2A10	ENSG00000197496.5	GLUT10		F	glucose, galactose
21	SLC2A11	ENSG00000133460.19	GLUT11	GLUT10	F	glucose, fructose
22	SLC2A12	ENSG00000146411.5	GLUT12	GLUT8	F	glucose
23	SLC2A13	ENSG00000151229.12	HMIT	GLUT13	C / H+	myo-inositol
24	SLC2A14	ENSG00000173262.11	GLUT14	SLC2A3P3	O	
25	SLC3A1	ENSG00000138079.13	rBAT	NBAT, D2H	E (see details in SLC7 table)	system b0,+, heterodimerizes with light subunit SLC7A9
26	SLC3A2	ENSG00000168003.16	4F2hc	CD98hc, FRP	E (see details in SLC7 table)	systems L, y+L, xc- and asc with light subunits SLC7A5-8 and SLC7A10-11
27	SLC4A1	ENSG00000004939.13	AE1	Band 3	E	chloride cicarbonate
28	SLC4A2	ENSG00000164889.12	AE2		E	chloride bicarbonate

29	SLC4A3	ENSG00000114923.16	AE3		E	chloride bicarbonate
30	SLC4A4	ENSG00000080493.13	NBCe1	NBC, NBC1	C	sodium bicarbonate (and/or carbonate)
31	SLC4A5	ENSG00000188687.15	NBCe2	NBC4	C	sodium bicarbonate (and/or carbonate)
32	SLC4A7	ENSG00000033867.16	NBCn1	NBC2, NBC3, SLC4A6	C	chloride bicarbonate
33	SLC4A8	ENSG00000050438.16	NDCBE	kNBC3	C, E	sodium bicarbonate chloride
34	SLC4A9	ENSG00000113073.14		AE4		inconclusive
35	SLC4A10	ENSG00000144290.16	NBCn2	NCBE	C, E	sodium bicarbonate chloride
36	SLC4A11	ENSG00000088836.12	BTR1	NaBC1	C	sodium, borate
37	SLC5A1	ENSG00000100170.9	SGLT1	None	C / Na+, (H+), F / Na+, (H+), Ch / urea, water	glucose and galactose (urea and water)
38	SLC5A2	ENSG00000140675.12	SGLT2	None	C / Na+	glucose
39	SLC5A3	ENSG00000198743.5	SMIT1	None	C / Na+	myoinositol (glucose)
40	SLC5A4	ENSG00000100191.5	SGLT3	SAAT1	glucose activated Na+, (H+) channel	Na+ (H+)
41	SLC5A5	ENSG00000105641.3	NIS		C / Na+, F / Na+, Ch / urea, water	I- (ClO4-, SCN-, NO3-, Br-)
42	SLC5A6	ENSG00000138074.14	SMVT		C / Na+	biotin, lipoate panthothenate, I-
43	SLC5A7	ENSG00000115665.8	CHT	CHT1		choline
44	SLC5A8	ENSG00000256870.2	SMCT1	AIT	C / Na+	short chain fatty acids
45	SLC5A9	ENSG00000117834.12	SGLT4		C / Na+	mannose, fructose, glucose
46	SLC5A10	ENSG00000154025.15	SGLT5	RK-D	C / Na+	mannose, fructose, glucose
47	SLC5A11	ENSG00000158865.12	SMIT2	SGLT6, KST1	C / Na+	myoinositol, chiro-inositol
48	SLC5A12	ENSG00000148942.14	SMCT2		C / Na+	short chain fatty acids
49	SLC6A1	ENSG00000157103.10		GAT1		GABA
50	SLC6A2	ENSG00000103546.18		NAT1, NET1, NET		norepinephrine
51	SLC6A3	ENSG00000142319.17		DAT, DAT1		dopamine
52	SLC6A4	ENSG00000108576.9		5-HTT, SERT		serotonin
53	SLC6A5	ENSG00000165970.11		GlyT2		glycine
54	SLC6A6	ENSG00000131389.16		TauT		taurine
55	SLC6A7	ENSG00000011083.8		PROT		proline
56	SLC6A8	ENSG00000130821.15		CRTR, CT1		creatine
57	SLC6A9	ENSG00000196517.11		GlyT1		glycine

58	SLC6A10P	ENSG00000214617.9	pseudogene	CT2		
59	SLC6A11	ENSG00000132164.9		GAT-B, GAT-3		GABA
60	SLC6A12	ENSG00000111181.12		BGT1		betaine, GABA
61	SLC6A13	ENSG00000010379.15		GAT2		GABA
62	SLC6A14	ENSG00000268104.2		ATB0+, beta-alanine carrier system		neutral, cationic amino acids
63	SLC6A15	ENSG00000072041.16		B0AT2, v7-3, NTT7-3		large, neutral amino acids
64	SLC6A16	ENSG00000063127.15		NTT5		unknown
65	SLC6A17	ENSG00000197106.6		NTT4, XT1		neutral amino acids
66	SLC6A18	ENSG00000164363.9		B0AT3, XT2		neutral amino acids
67	SLC6A19	ENSG00000174358.15		B0AT1, HND		neutral amino acids
68	SLC6A20	ENSG00000163817.15		system IMINO, XT3, Xtrp3		proline, pipecolate, sarcosine
69	SLC7A1	ENSG00000139514.12	CAT-1	ATRC1, system y+	F (non-obligatory E)	cationic L-amino acids
70	SLC7A2	ENSG00000003989.16	CAT-2 (A or B)	ATRC2, system y+	F	cationic L-amino acids
71	SLC7A3	ENSG00000165349.11	CAT-3	ATRC3, system y+	F	cationic L-amino acids
72	SLC7A4	ENSG00000099960.12	CAT-4		O	
73	SLC7A5	ENSG00000103257.8	LAT1	[4F2hc], 4F2lc, system L	E (similar intra- and extracellular selectivities, lower intracellular apparent affinity)	large neutral L-amino acids, T3, T4, L-DOPA, BCH
74	SLC7A5P1	ENSG00000260727.1	pseudogene	LAT1-3TM, MLAS		
75	SLC7A6	ENSG00000103064.13	y+LAT2	[4F2hc], system y+L	E (preferentially intracellular cationic amino acid against extracellular neutral amino acid / Na+)	cationic amino acids (Na+ indep.), large neutral L-amino acids (Na+ dep.)
76	SLC7A7	ENSG00000155465.18	y+LAT1	[4F2hc], system y+L	E (preferentially intracellular cationic amino acid against extracellular neutral amino acid / Na+)	cationic amino acids (Na+ indep.), large neutral L-amino acids (Na+ dep.)
77	SLC7A8	ENSG00000092068.18	LAT2	[4F2hc], system L	E (similar intra- and extracellular selectivities, lower intracellular apparent affinity)	neutral L-amino acids, T3, T4, BCH
78	SLC7A9	ENSG00000021488.12	b0,+AT	[rBAT], system b0,+	E (preferentially extracellular cationic amino acid and cystine against intracellular neutral amino acid)	cationic amino acids, large neutral amino acids

79	SLC7A10	ENSG00000130876.11	Asc-1	[4F2hc], system asc	preferentially E	small neutral amino acids
80	SLC7A11	ENSG00000151012.13	xCT	[4F2hc], system xc-	E (preferentially extracellular cystine against intracellular glutamate)	cystine (anionic form), L-glutamate
81	SLC7A13	ENSG00000164893.8	AGT-1	XAT2	E	L-aspartate, L-glutamate
82	SLC7A14	ENSG00000013293.5			O	
83	SLC7A15P	ENSG00000232800.1	pseudogene			
84	SLC8A1	ENSG00000183023.18	NCX1	NACA, NCE	E / Na+, Ca2+	Na+, Ca2+
85	SLC8A2	ENSG00000118160.13	NCX2		E / Na+, Ca2+	Na+, Ca2+
86	SLC8A3	ENSG00000100678.18	NCX3		E / Na+, Ca2+	Na+, Ca2+
87	SLC8B1	ENSG00000089060.11	NCLX	mNCX	E / Na+, Li+, Ca2+	Na+, Li+, Ca2+
88	SLC9A1	ENSG00000090020.10	NHE1	APNH?	E / Na+, H+	Na+, Li+, H+, NH4+
89	SLC9A2	ENSG00000115616.2	NHE2		E / Na+, H+	Na+, Li+, H+, NH4+
90	SLC9A3	ENSG00000066230.10	NHE3		E / Na+, H+	Na+, Li+, H+, NH4+
91	SLC9A3P1	ENSG00000233011.1	pseudogene			
92	SLC9A3P2	ENSG00000238125.1	pseudogene			
93	SLC9A3P3	ENSG00000226631.1	pseudogene			
94	SLC9A4	ENSG00000180251.4	NHE4		E / Na+, H+	Na+, Li+ (?), H+, NH4+
95	SLC9A5	ENSG00000135740.16	NHE5		E / Na+, H+	Na+, Li+, H+, NH4+ (?)
96	SLC9A6	ENSG00000198689.9	NHE6		E / Na+ (K+), H+	Na+, K+, H+
97	SLC9A7	ENSG00000065923.9	NHE7		E / Na+ (K+), H+	Na+, K+, Li+, H+, NH4+ (?)
98	SLC9A7P1	ENSG00000227825.4	pseudogene, partially processed			
99	SLC9A8	ENSG00000197818.11	NHE8		E / Na+ (K+), H+	Na+, K+, H+
100	SLC9A9	ENSG00000181804.14	NHE9		E / Na+ (K+), H+	Na+, K+, H+
101	SLC9B1	ENSG00000164037.16	NHA1	NHEDC1		
102	SLC9B2	ENSG00000164038.14	NHA2	NHEDC2		Na+, Li+
103	SLC9C1	ENSG00000172139.14	Sperm-NHE			Na+, H+
104	SLC9C2	ENSG00000162753.11				
105	SLC10A1	ENSG00000100652.4	NTCP	LBAT	C / Na+	bile acids
106	SLC10A2	ENSG00000125255.6	ASBT	IBAT, ISBT, NTCP2	C / Na+	bile acids
107	SLC10A3	ENSG00000126903.15	P3		O	

108	SLC10A4	ENSG00000145248.6	P4		O	
109	SLC10A5	ENSG00000253598.1	P5		O	
110	SLC10A6	ENSG00000145283.7	SOAT	P6	C / Na+	estrone-3-sulfate, dehydroepiandrosterone sulfate, pregnenolone sulfate
111	SLC10A7	ENSG00000120519.14	P7	C4orf13	O	
112	SLC11A1	ENSG00000018280.16	NRAMP1	NRAMP, LSH	E or C ? / H+	Mn2+, Fe2+, other divalent metal ions
113	SLC11A2	ENSG00000110911.14	DMT1	NRAMP2, DCT1	C / H+	Fe2+, Cd2+, Co2+, Cu1+, Mn2+, Ni2+, Pb2+, Zn2+
114	SLC12A1	ENSG00000074803.17	NKCC2			Na+, K+, Cl-
115	SLC12A2	ENSG00000064651.13	NKCC1			Na+, K+, Cl-
116	SLC12A3	ENSG00000070915.9	NCC			Na+, Cl-
117	SLC12A4	ENSG00000124067.16	KCC1			K+, Cl-
118	SLC12A5	ENSG00000124140.12	KCC2			K+, Cl-
119	SLC12A6	ENSG00000140199.11	KCC3			K+, Cl-
120	SLC12A7	ENSG00000113504.19	KCC4			K+, Cl-
121	SLC12A8	ENSG00000221955.10	CCC9			unknown
122	SLC12A9	ENSG00000146828.17	CIP			polyamines?
123	SLC13A1	ENSG00000081800.8	NaS1	Na-sulfate, NaSi-1	C / Na+	sulfate, selenate, thiosulfate
124	SLC13A2	ENSG00000007216.14	NaC1	SDCT1, NaDC-2, NaDC1	C / Na+	succinate, citrate, ?-ketoglutarate
125	SLC13A3	ENSG00000158296.13	NaC3	NaDC3, NaC3, SDCT2	C / Na+	succinate, citrate, ?-ketoglutarate, NALA, glutarate and its derivatives
126	SLC13A4	ENSG00000164707.15	NaS2	SUT1	C / Na+	sulfate, oxyanions selenium and chromium
127	SLC13A5	ENSG00000141485.15	NaC2	NaCT	C / Na+	citrate, succinate, pyruvate
128	SLC14A1	ENSG00000141469.16		UT3, UT11, JK, Kidd antigen		
129	SLC14A2	ENSG00000132874.13		UT1		
130	SLC15A1	ENSG00000088386.15	PEPT1	oligopeptide transporter 1, H+-peptide transporter 1	C / H+	di- and tri-peptides, protons, beta-lactam antibiotics
131	SLC15A2	ENSG00000163406.10	PEPT2	oligopeptide transporter 2, H+-peptide transporter 2	C / H+	di- and tri-peptides, protons, beta-lactam antibiotics
132	SLC15A3	ENSG00000110446.9	PHT2	peptide/histidine transporter 2, PTR3	C / H+	di- and tri-peptides, protons, histidine
133	SLC15A4	ENSG00000139370.10	PHT1	peptide/histidine transporter 1, PTR4	C / H+	di- and tri-peptides, protons, histidine

134	SLC16A1	ENSG00000155380.11	MCT1,	MOT1	C / H+ or E / monocarboxylate	lactate, pyruvate, ketone bodies
135	SLC16A2	ENSG00000147100.9	MCT8	MOT8, XPCT, MCT7	F	T2, rT3, T3, T4
136	SLC16A3	ENSG00000141526.14	MCT4	MOT4, MCT3	C / H+	lactate, ketone bodies
137	SLC16A4	ENSG00000168679.17	MCT5	MOT5, MCT4	O	
138	SLC16A5	ENSG00000170190.15	MCT6	MOT6, MCT5		bumetanide, probenecid, nateglinide ?
139	SLC16A6	ENSG00000108932.11	MCT7	MOT7, MCT6	O	
140	SLC16A7	ENSG00000118596.11	MCT2	MOT2	C / H+	pyruvate, lactate, ketone bodies
141	SLC16A8	ENSG00000100156.10	MCT3	MOT3, REMP	C / H+ (pH dependent but cotransport not confirmed experimentally)	lactate
142	SLC16A9	ENSG00000165449.11	MCT9	MOT9	O	
143	SLC16A10	ENSG00000112394.16	TAT1, MCT10	MOT10	F	aromatic amino acids, T3, T4
144	SLC16A11	ENSG00000174326.11	MCT11	MOT11	O	
145	SLC16A12	ENSG00000152779.13	MCT12	MOT12	O	
146	SLC16A13	ENSG00000174327.6	MCT13	MOT13	O	
147	SLC16A14	ENSG00000163053.10	MCT14	MOT14	O	
148	SLC17A1	ENSG00000124568.10	NPT1	NaPi1	electrogenic, Cl- dependent; C/Na+; channel	organic anions, phosphate, chloride
149	SLC17A2	ENSG00000112337.10	NPT3		unknown	unknown
150	SLC17A3	ENSG00000124564.17	NPT4		electrogenic	organic anions
151	SLC17A4	ENSG00000146039.10	Na+/PO4-cotransporter homologue		unknown	unknown
152	SLC17A5	ENSG00000119899.12	sialin	AST, VEAT	C / H+	sialic acid, other acidic sugars
153	SLC17A6	ENSG00000091664.7	VGLUT2	DNPI	electrogenic, Cl- dependent	glutamate
154	SLC17A7	ENSG00000104888.9	VGLUT1	BNPI	electrogenic, Cl- dependent	glutamate
155	SLC17A8	ENSG00000179520.10	VGLUT3		electrogenic, Cl- dependent	glutamate
156	SLC17A9	ENSG00000101194.17	VNUT		electrogenic, Cl- dependent	purine nucleotides
157	SLC18A1	ENSG00000036565.14	VMAT1	CGAT, VAT	E / H+	5-HT, DA, NE, epinephrine
158	SLC18A2	ENSG00000165646.11	VMAT2	SVAT, SVMT, VAT2, MAT	E / H+	5-HT, DA, NE, epinephrine, histamine
159	SLC18A3	ENSG00000187714.6	VAChT	Unc-17, Vesamicol binding protein	E / H+	acetylcholine
160	SLC18B1	ENSG00000146409.10	C6orf192	hypothetical human protein LOC116843, dJ55C23.6 gene	unknown	unknown

				product		
161	SLC19A1	ENSG00000173638.18	RFC	RFC1, RFT	E / organic phosphates	reduced folates, antifolates
162	SLC19A2	ENSG00000117479.12	THTR1	ThTr1	F	thiamine
163	SLC19A3	ENSG00000135917.13	THTR2	ThTr2	F	thiamine
164	SLC20A1	ENSG00000144136.10	PiT-1	gibbon ape leukemia virus receptor 1, GLVR1, Glvr1, FLJ41426, DKFZp686J2397	C / Na+, H2PO4-	inorganic phosphate (monovalent)
165	SLC20A2	ENSG00000168575.9	PiT-2	amphotropic murine leukemia virus receptor 2, GLVR2, Glvr-2, MLVAR	C / Na+, H2PO4-	inorganic phosphate (monovalent)
166	SLCO2A1	ENSG00000174640.12	OATP2A1			prostaglandins (C/lactate)
167	SLCO4A1	ENSG00000101187.15		OATP-E, OATP4A1		
168	SLCO5A1	ENSG00000137571.10		OATPRP4, OATP-J, OATP5A1		
169	SLCO3A1	ENSG00000176463.13		OATP-D, OATP3A1		
170	SLCO6A1	ENSG00000205359.9		OATP6A1, OATPY, MGC26949, CT48		
171	SLCO1A2	ENSG00000084453.16	OATP1A2			bile salts, organic anions and cations
172	SLCO2B1	ENSG00000137491.14	OATP2B1			E-3-S, DHEAS
173	SLCO1B1	ENSG00000134538.2	OATP1B1			bile salts, organic anions
174	SLCO1B3	ENSG00000111700.12	OATP1B3			bile salts, organic anions
175	SLCO4C1	ENSG00000173930.8		SLC21A20, OATP4C1, OATPX, OATP-H		
176	SLCO1C1	ENSG00000139155.8	OATP1C1			T4, T3, rT3
177	SLC22A1	ENSG00000175003.12	01-Oct		F	organic cations
178	SLC22A2	ENSG00000112499.12	02-Oct		F	organic cations
179	SLC22A3	ENSG00000146477.5	02-Oct	EMT (uptake-2 system)	F	organic cations
180	SLC22A4	ENSG00000197208.5	OCTN1	ETT	C / Na+	ergothioneine, zwitterions, organic cations
181	SLC22A5	ENSG00000197375.12	OCTN2	CT1, CDSP	C / Na+, L-carnitine, F / organic cations	zwitterions (L-carnitine), organic cations
182	SLC22A6	ENSG00000197901.11	OAT1	PAHT, NKT	E / organic anions	organic anions
183	SLC22A7	ENSG00000137204.14	OAT2	NLT	F or E	organic anions
184	SLC22A8	ENSG00000149452.15	OAT3		E / dicarboxylate	organic anions

185	SLC22A9	ENSG00000149742.9	OAT7	UST3, OAT4	E / short chain fatty acids	organic anions
186	SLC22A10	ENSG00000184999.11	OAT5		O	not determined
187	SLC22A11	ENSG00000168065.15	OAT4		F or E	organic anions
188	SLC22A12	ENSG00000197891.11	URAT1	RST, OAT4L	E / organic anions	urate, organic anions
189	SLC22A13	ENSG00000172940.11	OAT10	ORCTL3, OCTL1	E / anions	urate, organic anions
190	SLC22A14	ENSG00000144671.10	OCTL2	ORCTL4	O	not determined
191	SLC22A15	ENSG00000163393.12	FLIPT1		O	not determined
192	SLC22A16	ENSG00000004809.13	39726	FLIPT2, CT2	F	L-carnitine, noncharged compounds
193	SLC22A17	ENSG00000092096.14	BOIT	BOCT, NGALR	O	not determined
194	SLC22A18	ENSG00000110628.13		IMPT1, TSSC5, ORCTL2		probably organic anions
195	SLC22A20	ENSG00000197847.12	OAT6		E / dicarboxylate	probably organic anions
196	SLC22A23	ENSG00000137266.14			O	
197	SLC22A24	ENSG00000197658.9			O	
198	SLC22A25	ENSG00000196600.12	UST6	HIMTP	O	
199	SLC22A31	ENSG00000259803.6				
200	SLC22A32	ENSG00000109736.14	MFSD10	TETRAN	C / H+	p-aminohippuric acid (PAH), indomethacin, diclofenac, mefenamic acid, etodolac [PMID: 18638446]
201	SLC22B1	ENSG00000159164.9	SV2A			Galactose [PMID: 25326386]; also binds levetiracetam [PMID: 15210974], selectracetam [PMID: 18183537], brivaracetam [PMID: 17785672].
202	SLC22B2	ENSG00000185518.11	SV2B			
203	SLC22B3	ENSG00000122012.13	SV2C			
204	SLC22B4	ENSG00000166111.9	SVOP			Nicotinate [PMID: 21953179]; binds nucleotides in the TM region [PMID: 19390693]
205	SLC22B5	ENSG00000157703.15	SVOPL			
206	SLC23A1	ENSG00000170482.16	SVCT1	YSPL3, NCBT2, MGC22361	C / Na+	L-ascorbic acid
207	SLC23A2	ENSG00000089057.14	SVCT2	YSPL2, KIAA0238, NBTL1, NCBT1	C / Na+	L-ascorbic acid
208	SLC23A3	ENSG00000213901.10	SVCT3	YSPL1, FLJ31168, E2BP3		
209	SLC24A1	ENSG00000074621.13	NCKX1	RODX, HsT17412, KIAA0702	E / 4 Na+, Ca2+, K+	Na+, Ca2+, K+
210	SLC24A2	ENSG00000155886.11	NCKX2		E / 4 Na+, Ca2+, K+	Na+, Ca2+, K+
211	SLC24A3	ENSG00000185052.11	NCKX3		E / 4 Na+, Ca2+, K+	Na+, Ca2+, K+

212	SLC24A4	ENSG00000140090.17	NCKX4	SHEP6, FLJ38852	E / 4 Na+, Ca2+, K+	Na+, Ca2+, K+
213	SLC24A5	ENSG00000188467.10	NCKX5	JSX, SHEP4	E / 4 Na+, Ca2+, K+	Na+, Ca2+, K+
214	SLC25A1	ENSG00000100075.9	CIC (citrate carrier)	CTP, tricarboxylate carrier, SLC20A3	E / citrate-H+, malate	citrate, isocitrate, malate, PEP
215	SLC25A2	ENSG00000120329.6	ORC2 (ornithine carrier 2)	ORNT2	E / ornithine, citrulline, H+; ornithine, H+	ornithine, citrulline, lysine, arginine, histidine
216	SLC25A3	ENSG00000075415.12	PHC (phosphate carrier)	PTP, PiC	C / phosphate, H+; E / phosphate, OH-	phosphate
217	SLC25A4	ENSG00000151729.10	ANT1 (adenine nucleotide translocase-1)	AAC1, T1, PEO2, PEO3	E / ADP, ATP	ADP, ATP
218	SLC25A5	ENSG00000005022.5	ANT2 (adenine nucleotide translocase-2)	AAC2, T2	E / ADP, ATP	ADP, ATP
219	SLC25A5P1	ENSG00000215347.3	pseudogene	bK250D10, ANTP3		
220	SLC25A6	ENSG00000169100.12	ANT3 (adenine nucleotide translocase-3)	AAC3, T3	E / ADP, ATP	ADP, ATP
221	SLC25A6P1	ENSG00000233015.1	pseudogene			
222	SLC25A7	ENSG00000109424.3	UCP1 (uncoupling protein 1)	thermogenin, proton carrier	F	H+
223	SLC25A8	ENSG00000175567.8	UCP2 (uncoupling protein 2)	proton carrier, UCPH	F	H+
224	SLC25A9	ENSG00000175564.12	UCP3 (uncoupling protein 3)	proton carrier	F	H+
225	SLC25A10	ENSG00000183048.11	DIC (dicarboxylate carrier)		E / malate, phosphate	malate, phosphate, succinate, sulphate, thiosulphate
226	SLC25A11	ENSG00000108528.13	OGC (oxoglutarate carrier)	oxoglutarate / malate carrier, SLC20A4	E / oxoglutarate, malate	2-oxoglutarate, malate
227	SLC25A12	ENSG00000115840.13	AGC1 (aspartate / glutamate carrier 1)	aralar1	E / aspartate, glutamate, H+	aspartate, glutamate
228	SLC25A13	ENSG00000004864.13	AGC2 (aspartate / glutamate carrier 2)	citrin, CTLN2, aralar2	E / aspartate, glutamate, H+	aspartate, glutamate
229	SLC25A14	ENSG00000102078.15	UCP5 (uncoupling protein 5)	BMCP1, brain mitochondrial carrier protein	O	
230	SLC25A15	ENSG00000102743.14	ORC1 (ornithine	ORNT1, HHH	E / ornithine, citrulline, H+;	ornithine, citrulline, lysine, arginine

			carrier 1)		ornithine, H+	
231	SLC25A15P1	ENSG00000227629.1	pseudogene			
232	SLC25A16	ENSG00000122912.14	GDC (Graves' disease carrier)	GDA, ML7, HGT.1	O	
233	SLC25A17	ENSG00000100372.14		PMP34, ANC1, ANT1	E / CoA; PAP, CoA, dPCoA; CoA,AMP; FAD, FMN; NAD+, AMP	CoA, FAD, NAD+, AMP, ADP, PAP, dPCoA, FMN
234	SLC25A18	ENSG00000182902.13	GC2 (glutamate carrier 2)		C / glutamate, H+; E / glutamate, OH-	glutamate
235	SLC25A19	ENSG00000125454.11	DNC (deoxynucleotide carrier)	MUP1, TPC, MCPHA	E / thiamine pyrophosphate, thiamine monophosphate, thiamine; pyrophosphate, (deoxy)nucleotide	thiamine pyrophosphate, thiamine monophosphate, (deoxy)nucleotides
236	SLC25A20	ENSG00000178537.9	CAC (carnitine / acylcarnitine carrier)	CACT, carnitine carrier	E / carnitine, acylcarnitine; F (at slow rate)	carnitine, acylcarnitine
237	SLC25A20P1	ENSG00000218363.1	pseudogene			
238	SLC25A21	ENSG00000183032.10	ODC (oxoadipate carrier)		E / oxoadipate, oxoglutarate	oxoadipate, oxoglutarate
239	SLC25A22	ENSG00000177542.10	GC1 (glutamate carrier 1)		C / glutamate, H+; E / glutamate, OH-	glutamate
240	SLC25A23	ENSG00000125648.14	APC2	ScaMC-3, MCSC2	E / ATP-Mg, Pi	ATP-Mg2+, ATP, ADP, AMP, Pi
241	SLC25A24	ENSG00000085491.15	APC1	ScaMC-1	E / ATP-Mg, Pi	ATP-Mg2+, ATP, ADP, AMP, Pi
242	SLC25A25	ENSG00000148339.12	APC3	ScaMC-2, MCSC, PCSCL	O	
243	SLC25A26	ENSG00000144741.17	SAMC		E / S-adenosyl-methionine, S-adenosyl-homocysteine	S-adenosyl-methionine, S-adenosyl-homocysteine
244	SLC25A27	ENSG00000153291.15	UCP4 (uncoupling protein 4)		O	
245	SLC25A28	ENSG00000155287.10	Mitoferrin 2 (Mfrn2)	MRS3 / 4, MRS4L		Fe2+
246	SLC25A29	ENSG00000197119.12	ORNT3	CACL		ornithine, acylcarnitine
247	SLC25A30	ENSG00000174032.16		KMCP1, UCP6	O	
248	SLC25A31	ENSG00000151475.5	AAC4, ANT4 (adenine nucleotide carrier 4)	SFEC	E / ADP, ATP	ADP, ATP
249	SLC25A32	ENSG00000164933.11	MFT	MFTC		folate
250	SLC25A33	ENSG00000171612.6	PNC1 (pyrimidine nucleotide	BMSC-MCP		UTP

			carrier 1)			
251	SLC25A34	ENSG00000162461.7			O	
252	SLC25A35	ENSG00000125434.10			O	
253	SLC25A36	ENSG00000114120.11	PNC2 (pyrimidine nucleotide carrier 2)			pyrimidine nucleotides
254	SLC25A37	ENSG00000147454.13	Mitoferrin 1 (Mfrn1)	HT015, MSC, MSCP		Fe2+
255	SLC25A38	ENSG00000144659.10				glycine ?
256	SLC25A39	ENSG00000013306.15		CGI-69	O	
257	SLC25A40	ENSG00000075303.12		MCFP	O	
258	SLC25A41	ENSG00000181240.13	APC4	SCaMC-3L	E	ATP-Mg / Pi
259	SLC25A42	ENSG00000181035.13			E	CoA, ADP, ATP, adenosine 3',5'-diphosphate, dPCoA
260	SLC25A43	ENSG00000077713.18			O	
261	SLC25A44	ENSG00000160785.13			O	
262	SLC25A45	ENSG00000162241.12			O	
263	SLC25A46	ENSG00000164209.16			O	
264	SLC25A47	ENSG00000140107.10		C14orf68, HDMCP	O	
265	SLC25A48	ENSG00000145832.12		FLJ44862	O	
266	SLC25A49	ENSG00000137409.18	MTCH1	MCH1	O	
267	SLC25A50	ENSG00000109919.9	MTCH2	MCH2	O	
268	SLC25A51	ENSG00000122696.12	MCART1	MCTR1	O	
269	SLC25A51P1	ENSG00000220483.4	pseudogene	MCART3P		
270	SLC25A51P2	ENSG00000263716.1	pseudogene	MCART4P		
271	SLC25A51P3	ENSG00000254181.1	pseudogene	MCART5P		
272	SLC25A52	ENSG00000141437.8	MCART2	MCTR2	O	
273	SLC25A53	ENSG00000269743.2	MCART6	MCTR6	O	
274	SLC26A1	ENSG00000145217.13	SAT1, SLC26A1		E / SO42-, oxalate, glyoxylate	SO42-, oxalate, glyoxylate
275	SLC26A2	ENSG00000155850.7	DTDST, SLC26A2		E / SO42-, oxalate, Cl-	SO42-, oxalate, Cl-
276	SLC26A3	ENSG00000091138.12	DRA, CLD, SLC26A3		E / Cl-, HCO3-, oxalate	Cl-, HCO3-, oxalate
277	SLC26A4	ENSG00000091137.11	PDS, Pendrin, SLC26A4		E / I-, Cl-, HCO3-	I-, Cl-, HCO3-

278	SLC26A5	ENSG00000170615.14	Prestin, SLC26A5		E / Cl-, formate, oxalate, SO42-	Cl-, formate, oxalate, SO42-
279	SLC26A6	ENSG00000225697.10	CFEX, PAT1, SLC26A6		E / Cl-, HCO3-, oxalate, OH-, formate	Cl-, HCO3-, oxalate, OH-, formate
280	SLC26A7	ENSG00000147606.8	SUT2, SLC26A7		E / Cl-, HCO3-, OH-, SO42-, Ch / Cl-	Cl-, HCO3-, OH-, SO42-, Ch: Cl-
281	SLC26A8	ENSG00000112053.13	TAT1, SLC26A8		E / Cl-, HCO3-, OH-	Cl-, HCO3-, OH-
282	SLC26A9	ENSG00000174502.18	SLC26A9		E / Cl-, HCO3, Ch / Cl-, HCO3-	Cl-, HCO3, Ch: Cl-, HCO3-
283	SLC26A10	ENSG00000135502.16	(SLC26A10)			
284	SLC26A11	ENSG00000181045.14	SUT1, KBAT, SLC26A11		E / Cl-, HCO3-, SO42-, oxalate, ?Ch / Cl-	Cl-, HCO3-, SO42-, oxalate (?), Ch: Cl-
285	SLC27A1	ENSG00000130304.16	FATP1	FATP, ACSVL5	LCFA transport, VLCFA activation	LCFA, VLCFA
286	SLC27A2	ENSG00000140284.10	FATP2	ACSVL1, VLCS, VLACS, FACVL1	LCFA transport, VLCFA activation	LCFA, VLCFA
287	SLC27A3	ENSG00000143554.13	FATP3	ACSVL3, VLCS-3	LCFA transport, VLCFA activation	LCFA, VLCFA
288	SLC27A4	ENSG00000167114.12	FATP4	ACSVL4	LCFA transport, VLCFA activation	LCFA, VLCFA
289	SLC27A5	ENSG00000083807.9	FATP5	ACSVL6, VLCS-H2, VLACSR, FACVL3, BAL, ACSB, BACS	LCFA transport, bile acid conjugation	LCFA, bile acids
290	SLC27A6	ENSG00000113396.12	FATP6	ACSVL2, VLCS-H1, FACVL2	LCFA transport, VLCFA activation	LCFA, VLCFA
291	SLC28A1	ENSG00000156222.11	CNT1		concentrative 1:1 sodium:nucleoside	
292	SLC28A2	ENSG00000137860.11	CNT2		concentrative 1:1 sodium:nucleoside	
293	SLC28A3	ENSG00000197506.7	CNT3		concentrative 2:1 sodium: nucleoside 1:1 proton: nucleoside probably 1:1:1 sodium:proton:nucleoside	
294	SLC29A1	ENSG00000112759.16	ENT1		F	
295	SLC29A2	ENSG00000174669.11	ENT2		F	
296	SLC29A3	ENSG00000198246.7	ENT3		unclear, possibly proton-linked	
297	SLC29A4	ENSG00000164638.10	ENT4		unclear, possibly proton-linked	
298	SLC30A1	ENSG00000170385.9		ZnT1		
299	SLC30A2	ENSG00000158014.14		ZnT2		
300	SLC30A3	ENSG00000115194.10		ZnT3		

301	SLC30A4	ENSG00000104154.6		ZnT4		
302	SLC30A5	ENSG00000145740.18		ZnT5		
303	SLC30A6	ENSG00000152683.14		ZnT6		
304	SLC30A7	ENSG00000162695.11		ZnT7		
305	SLC30A8	ENSG00000164756.12		ZnT8		
306	SLC30A9	ENSG00000014824.13		ZnT9		
307	SLC30A10	ENSG00000196660.10		ZnT10		
308	SLC31A1	ENSG00000136868.10	CTR1	hCtr1, Ctr1, COPT1	energy in-dependent, potassium-dependent	copper (I), cisplatin
309	SLC31A1P1	ENSG00000224426.1	pseudogene	SLC31A1P, CTR1psi, CTR1P		
310	SLC31A2	ENSG00000136867.10	CTR2	hCtr2, Ctr2, COPT2	unknown	copper, cisplatin
311	SLC32A1	ENSG00000101438.3	VIAAT	VGAT	E / H+	GABA / glycine
312	SLC33A1	ENSG00000169359.13	ACATN1	AT-1	F	acetyl-CoA
313	SLC33A2	ENSG00000167700.8	MFSD3			
314	SLC34A1	ENSG00000131183.10	NaPi-IIa	Napi-3, NPT2, npt2	C / Na, HPO42-	inorganic phosphate (divalent)
315	SLC34A2	ENSG00000157765.11	NaPi-IIb		C / Na, HPO42-	inorganic phosphate (divalent)
316	SLC34A3	ENSG00000198569.9	NaPi-IIc		C / Na, HPO42-	inorganic phosphate (divalent)
317	SLC35A1	ENSG00000164414.16	CST	CMPST	E / CMP	CMP-sialic acid
318	SLC35A2	ENSG00000102100.14	UGT	UGAT, UGTL, UGALT, UGT1, UGT2	E / UMP	UDP-galactose, UDP-N-acetylgalactosamine
319	SLC35A3	ENSG00000117620.12		DKFZp781P1297	E / UMP	UDP-N-acetylglucosamine
320	SLC35A4	ENSG00000176087.14		MGC2541		
321	SLC35A5	ENSG00000138459.8		FLJ11130, FLJ20730, FLJ25973, DKFZp434E102		
322	SLC35B1	ENSG00000121073.13		UGTREL1		
323	SLC35B2	ENSG00000157593.16	PAPST1	UGTrel4, SLL		PAPS
324	SLC35B3	ENSG00000124786.9	PAPST2	CGI-19, C6orf196		PAPS
325	SLC35B4	ENSG00000205060.10	YEA	YEA4, FLJ14697		UDP-xylose, UDP-N-acetylglucosamine
326	SLC35C1	ENSG00000181830.8	FUCT1	FLJ11320, FLJ14841	E / GMP	GDP-fucose
327	SLC35C2	ENSG00000080189.14	OVCOV1	CGI-15, C20orf5, FLJ37039, FLJ46434, MGC20633, MGC32079, MGC39183, BA394O2.1		GDP-fucose (?)
328	SLC35D1	ENSG00000116704.7	UGTREL7	KIAA0260, MGC138236	E / UMP	UDP-glucuronic acid, UDP-N-acetylgalactosamine

329	SLC35D2	ENSG00000130958.11	HFRC1	UGTrel8, Hfrc, SQV7L, MGC117215, MGC142139		UDP-N-acetylglucosamine
330	SLC35D3	ENSG00000182747.4	FRCL1	MGC102873, bA55K22.3		
331	SLC35D4	ENSG00000134490.13	TMEM241	C18orf45		
332	SLC35E1	ENSG00000127526.13		FLJ14251, FLJ36689, MGC44954, DKFZp564G0462		
333	SLC35E2	ENSG00000215790.6		FLJ34996, FLJ44537, KIAA0447, MGC104754, MGC117254, MGC126715, MGC138494, DKFZp686M0869		
334	SLC35E3	ENSG00000175782.9		BLOV1		
335	SLC35E4	ENSG00000100036.12		MGC129826		
336	SLC35F1	ENSG00000196376.10		C6orf169, FLJ13018, dJ230I3.1		
337	SLC35F2	ENSG00000110660.14		HSNOV1, FLJ13018, DKFZp667H1615		
338	SLC35F3	ENSG00000183780.12		FLJ37712		
339	SLC35F4	ENSG00000151812.14		C14orf36, FLJ37712, c14_5373		
340	SLC35F5	ENSG00000115084.12		FLJ22004		
341	SLC35F6	ENSG00000213699.8	C2orf18	ANT2BP		
342	SLC35G1	ENSG00000176273.14		TMEM20, C10orf60		
343	SLC35G2	ENSG00000168917.8		TMEM22		
344	SLC35G3	ENSG00000164729.7		AMAC1, TMEM21A		
345	SLC35G4	ENSG00000236396.6		AMAC1L1		
346	SLC35G5	ENSG00000177710.5		AMAC, AMAC1L2		
347	SLC35G6	ENSG00000259224.2		AMAC1L3, TMEM21B		
348	SLC36A1	ENSG00000123643.12	PAT1	LYAAT1	C / H+	GABA, P, G, beta-alanine
349	SLC36A2	ENSG00000186335.8	PAT2	tamodorin 1	C / H+	P, G, A, hydroxyproline
350	SLC36A3	ENSG00000186334.9	PAT3			
351	SLC36A4	ENSG00000180773.14	PAT4	LYAAT2	C / H+	P, tryptophan
352	SLC37A1	ENSG00000160190.13	SLC37A1, SPX1	glucose-6-phosphate	E / inorganic phosphate	
353	SLC37A2	ENSG00000134955.11	SLC37A2, SPX2	glucose-6-phosphate	E / inorganic phosphate	
354	SLC37A3	ENSG00000157800.17	SLC37A3, SPX3	unknown		
355	SLC37A4	ENSG00000137700.16	SLC37A4, G6PT, SPX4	glucose-6-phosphate	E / inorganic phosphate	
356	SLC38A1	ENSG00000111371.15	SNAT1	ATA1, NAT2, SAT1	C / Na+	Q, A, N, C, H, S

357	SLC38A2	ENSG00000134294.13	SNAT2	ATA2, SAT2	C / Na+	A, N, C, Q, G, H, M, P, S
358	SLC38A3	ENSG00000188338.14	SNAT3	SN1	C / Na+, E / H+	Q, H, A, N
359	SLC38A4	ENSG00000139209.15	SNAT4	ATA3, NAT3, PAAT	C / Na+	A, N, C, G, S, T
360	SLC38A5	ENSG00000017483.14	SNAT5	SN2	C / Na+, E / H+	Q, N, H, S
361	SLC38A6	ENSG00000139974.15	SNAT6			
362	SLC38A7	ENSG00000103042.8	SNAT7		? / Na+	Q, H, S ,A, N
363	SLC38A8	ENSG00000166558.10	SNAT8		? / Na+	Q, H, A, N [PMID: 25451601]
364	SLC38A9	ENSG00000177058.11	N/A			R, Q, H, P, K, E, L
365	SLC38A10	ENSG00000157637.12	N/A			
366	SLC38A11	ENSG00000169507.9	N/A			
367	SLC39A1	ENSG00000143570.17	ZIP1, ZIRTL			Zn
368	SLC39A2	ENSG00000165794.9	ZIP2, Eti-1, 6A1			Zn
369	SLC39A3	ENSG00000141873.10	ZIP3			Zn, not specific
370	SLC39A4	ENSG00000147804.9	ZIP4			Zn
371	SLC39A5	ENSG00000139540.11	ZIP5, LZT-Hs7			Zn
372	SLC39A6	ENSG00000141424.12	ZIP6, LIV-1			Zn
373	SLC39A7	ENSG00000112473.16	ZIP7, HKE4, RING5			Zn, Mn
374	SLC39A8	ENSG00000138821.12	ZIP8, BIGM103, LZT-Hs6			Zn, Cd, Mn
375	SLC39A9	ENSG00000029364.11	ZIP9			
376	SLC39A10	ENSG00000196950.13	ZIP10, LZT-Hs2			Zn
377	SLC39A11	ENSG00000133195.11	ZIP11			
378	SLC39A12	ENSG00000148482.11	ZIP12j, LZT-Hs8			Zn
379	SLC39A13	ENSG00000165915.13	ZIP13, LZT-Hs9			Zn
380	SLC39A14	ENSG00000104635.13	ZIP14, LZT-Hs4			Zn, Fe, Mn, Cd
381	SLC40A1	ENSG00000138449.10	ferroportin (FPN1)	MTP1, IREG1	F ?	ferrous iron
382	SLC41A1	ENSG00000133065.10	MgtE		Ch	Mg2+ (Sr2+, Zn2+, Cu2+, Fe2+, Co2+, Ba2+, Cd2+)
383	SLC41A2	ENSG00000136052.9		SLC41A1-L1, SLC41A1-like 1	Ch	Mg2+ (Ba2+, Ni2+, Co2+, Fe2+, Mn2+)
384	SLC41A3	ENSG00000114544.15		SLC41A1-L2, SLC41A1-like 2, FLJ20473	Ch? (predicted based on homology with the other 2 members/MgtE transporter)	

385	SLC42A1	ENSG00000112077.15	RhAG	RHAG (Rh50A)	H+	NH4+, NH3
386	SLC42A2	ENSG00000132677.12	RhBG	RHBG	electrogenic, no coupled ions	NH4+, NH3, methyl amine, methyl ammonium
387	SLC42A3	ENSG00000140519.12	RhCG	RHCG (RhGK)	electroneutral, possibly H+	NH4+, NH3
388	SLC43A1	ENSG00000149150.8	LAT3	POV1	F	L-BCAAs, amino alcohols
389	SLC43A2	ENSG00000167703.14	LAT4		F	L-BCAAs, amino alcohols
390	SLC43A3	ENSG00000134802.17	EEG1	FOAP-13	O	
391	SLC44A1	ENSG00000070214.15	CTL1	CDw92, CHTL1	E?	choline
392	SLC44A2	ENSG00000129353.14	CTL2	PP1292	O	choline
393	SLC44A3	ENSG00000143036.16	CTL3	MGC45474	O	
394	SLC44A4	ENSG00000204385.10	CTL4	NG22, FLJ14491	O	
395	SLC44A5	ENSG00000137968.16	CTL5	MGC34032	O	
396	SLC45A1	ENSG00000162426.14	SLC45A1	Past-A, DNB5	C / H+	glucose, galactose, sucrose
397	SLC45A2	ENSG00000164175.14	SLC45A2	MATP, AIM1, underwhite	C / H+ [PMID: 25164149]	sucrose, glucose, fructose [PMID: 25164149]
398	SLC45A3	ENSG00000158715.5	SLC45A3	Prostein	C / H+ [PMID: 25164149]	sucrose, glucose, fructose [PMID: 25164149]
399	SLC45A4	ENSG00000022567.9	SLC45A4	KIAA1126	C / H+ [PMID: 25164149]	sucrose, glucose, fructose [PMID: 25164149]
400	SLC46A1	ENSG00000076351.12	PCFT	HCP1	C / proton-symporter	Reduced folates, folic acid, antifolates
401	SLC46A2	ENSG00000119457.7	TSCOT	Ly110		unknown
402	SLC46A3	ENSG00000139508.14				Uncertain; lysosomal export of maytansine conjugates
403	SLC47A1	ENSG00000142494.13		MATE1		tetraethylammonium (TEA), 1-methyl-4-phenylpyridinium (MPP), cimetidine, metformin, guanidine, procainamide, cephalexin, cephradine
404	SLC47A2	ENSG00000180638.17		MATE2-K (MATE2, MATE2-B)		TEA, MPP, cimetidine, metformin, guanidine, procainamide
405	SLC48A1	ENSG00000211584.13	HRG-1		C / H+ ?	heme
406	SLC49A1	ENSG00000162769.12	FLVCR1	FLVCR, MFSD7B, AXPC1, PCARP	unknown	heme
407	SLC49A2	ENSG00000119686.9	FLVCR2	MFSD7C, CCT, EPV, PVHH, FLVCRL14q	unknown	heme
408	SLC49A3	ENSG00000169026.12	MFSD7	LP2561, FLJ22269	unknown	unknown
409	SLC49A4	ENSG00000138463.8	DIRC2	RCC4, FLJ14784	unknown	unknown
410	SLC50A1	ENSG00000169241.17	RAG1AP	HsSWEET1	F	glucose
411	SLC51A	ENSG00000163959.9	OST alpha	OST alpha-OST beta	F	bile acids
412	SLC51B	ENSG00000186198.3	OST beta			steroids

413	SLC52A1	ENSG00000132517.14		RFVT1, RFT1, GPR172B, PERV-A receptor 2, GPCR42, PAR2, FLJ10060	Na+, Cl-, pH-independent	
414	SLC52A2	ENSG00000185803.8		RFVT2, RFT3, GPR172A, PERV-A receptor 1, GPCR41, PAR1, D15Ertd747e, FLJ11856	Na+, Cl-, pH-independent	
415	SLC52A3	ENSG00000101276.14		RFVT3, RFT2, C20orf54, RIKEN cDNA 2310046K01, hypothetical protein LOC113278, bA371L19.1	C / Na+, Cl-indep., pH sensitive	
416	SLC53A1	ENSG00000143324.13	XPR1	SYG1, XR		Phosphate [PMID: 23791524]
417	SLC54A1	ENSG00000060762.18	MPC1	BRP44L		Pyruvate [PMID: 22628554] [PMID: 22628558]
418	SLC54A2	ENSG00000143158.10	MPC2	BRP44		Pyruvate [PMID: 22628558]
419	SLC54A3	ENSG00000238205.2	MPC1L			Pyruvate [PMID: 27317664]
420	SLC55A1	ENSG00000168924.14	LETM1	Mdm38 homolog (yeast)	E / Ca2+:H+[PMID: 19797662] [PMID: 27669901], E / K+:H+ [PMID: 15138253] [PMID: 17925330]	Ca2+, K+, H+ (K+: [PMID: 20197279] [PMID: 15138253], [PMID: 17925330], yeast protein: [PMID: 17541427]), arguments against Ca2+ transport [PMID: 20197279].
421	SLC55A2	ENSG00000165046.12	LETM2	FLJ25409		
422	SLC55A3	ENSG00000050426.15	LETMD1	HCCR, HCCR-1, HCCR-2		
423	SLC56A1	ENSG00000164466.12	SFXN1	FLJ12876		Probably falsely identified as a tricarboxylate carrier in [PMID: 8132491] as discussed in [PMID: 11274051]. Likely transports pyridoxin or another heme precursor or ALAS2 cofactor [PMID: 11274051] [PMID: 12670026].
424	SLC56A2	ENSG00000156398.12	SFXN2			
425	SLC56A3	ENSG00000107819.13	SFXN3			
426	SLC56A4	ENSG00000183605.16	SFXN4			
427	SLC56A5	ENSG00000144040.12	SFXN5			
428	SLC57A1	ENSG00000170113.15	NIPA1			Mg2+ [PMID: 17166836], also Sr2+, Fe2+ and Co2+ to a lesser extent [PMID: 18667602]
429	SLC57A2	ENSG00000140157.14	NIPA2			Mg2+ [PMID: 18667602]
430	SLC57A3	ENSG00000163293.11	NIPAL1	NIPA3 [PMID: 18667602]		Mg2+, Sr2+, Ba2+, Fe2+, Cu2+ [PMID: 18667602]

431	SLC57A4	ENSG00000104361.9	NIPAL2	NIPA4 [PMID: 18667602]		Mg2+, Sr2+, Ba2+[PMID: 18667602]
432	SLC57A5	ENSG00000001461.16	NIPAL3	NPAL3		
433	SLC57A6	ENSG00000172548.14	NIPAL4	ichthyin, NIPA4		
434	SLC58A1	ENSG00000102158.19	MAGT1		Channel-like [PMID: 19940067]	Mg2+ [PMID: 15804357]
435	SLC58A2	ENSG00000104723.20	TUSC3	N33		Mg2+, Fe2+, Cu2+, Mn2+ [PMID: 18667602] [PMID: 19940067]
436	SLC59A1	ENSG00000168389.17	MFSD2A	MFSD2	C / LPC:Na+, uptake [PMID: 24828044]	LPC (lysophosphatidylcholine) form of DHA (docosahexaenoic acid) [PMID: 24828044]
437	SLC59A2	ENSG00000205639.9	MFSD2B			
438	SLC60A1	ENSG00000174514.12	MFSD4A			
439	SLC61A1	ENSG00000182544.8	MFSD5			Molybdate [PMID: 21464289]
440	SLC62A1	ENSG00000154122.12	ANKH			Pyrophosphate [PMID: 10894769]
441	SLC63A1	ENSG00000169682.17	SPNS1			
442	SLC63A2	ENSG00000183018.8	SPNS2			Phosphorylated sphingolipids [PMID: 19074308]; phosphorylated Fingolimod (FTY720-P) [PMID: 21084291]; sphingosine-1-phosphate (S1P), dihydrosphingosine-1-phosphate (DH-S1P), phyto-S1P, C17-S1P [PMID: 21084291]
443	SLC63A3	ENSG00000182557.7	SPNS3			
444	SLC64A1	ENSG00000134851.12	TMEM165		E / Ca2+:H+	
445	SLC65A1	ENSG00000141458.12	NPC1			Cholesterol [PMID: 17989073] [PMID: 17989072] [PMID: 27410046]
446	SLC65A2	ENSG00000015520.14	NPC1L1			Cholesterol [PMID: 14976318]

* Abbreviations for transport type: C: Cotransporter; E: Exchanger; F: Facilitated transporter; O: Orphan transporter.
