# Supplementary material for: The Solute Carrier Superfamily as Therapeutic Targets in Pancreatic Ductal Adenocarcinoma
Source: Genes (Basel). 2025 Apr 18;16(4):463. doi: 10.3390/genes16040463 (PMC12027052; doi:10.3390/genes16040463)
Supplement: Supplementary file 1 [file genes-16-00463-s001.zip › Table S2.pdf]

Table S2. The phenotype information of normal and PDAC samples

No.	Group	sample	detailed_category	primary disease or tissue	primary_site	sample_type	gender	study
1	Normal	GTEX-13NZA-1726-SM-5J1NA	Pancreas	Pancreas	Pancreas	Normal Tissue	Male	GTEX
2	Normal	GTEX-1399R-0426-SM-5IJE3	Pancreas	Pancreas	Pancreas	Normal Tissue	Male	GTEX
3	Normal	GTEX-ZYT6-1326-SM-5E453	Pancreas	Pancreas	Pancreas	Normal Tissue	Male	GTEX
4	Normal	GTEX-131XE-1926-SM-5IFER	Pancreas	Pancreas	Pancreas	Normal Tissue	Male	GTEX
5	Normal	GTEX-ZAB5-0826-SM-5P9FU	Pancreas	Pancreas	Pancreas	Normal Tissue	Male	GTEX
6	Normal	GTEX-NPJ8-2126-SM-3MJGK	Pancreas	Pancreas	Pancreas	Normal Tissue	Male	GTEX
7	Normal	GTEX-V1D1-0726-SM-4JBH7	Pancreas	Pancreas	Pancreas	Normal Tissue	Male	GTEX
8	Normal	GTEX-VUSG-0326-SM-3GIJ7	Pancreas	Pancreas	Pancreas	Normal Tissue	Male	GTEX
9	Normal	GTEX-ZYW4-2126-SM-59HJ9	Pancreas	Pancreas	Pancreas	Normal Tissue	Male	GTEX
10	Normal	GTEX-VJYA-0826-SM-4KL1M	Pancreas	Pancreas	Pancreas	Normal Tissue	Male	GTEX
11	Normal	GTEX-XXEK-1726-SM-4BRVB	Pancreas	Pancreas	Pancreas	Normal Tissue	Male	GTEX
12	Normal	GTEX-ZV7C-0726-SM-59HKH	Pancreas	Pancreas	Pancreas	Normal Tissue	Male	GTEX
13	Normal	GTEX-13YAN-2126-SM-5Q5C4	Pancreas	Pancreas	Pancreas	Normal Tissue	Male	GTEX
14	Normal	GTEX-11NSD-0526-SM-5A5LT	Pancreas	Pancreas	Pancreas	Normal Tissue	Male	GTEX
15	Normal	GTEX-139YR-1526-SM-5IFJ1	Pancreas	Pancreas	Pancreas	Normal Tissue	Male	GTEX
16	Normal	GTEX-PW2O-0826-SM-48TC5	Pancreas	Pancreas	Pancreas	Normal Tissue	Male	GTEX
17	Normal	GTEX-145MO-2126-SM-5Q5CZ	Pancreas	Pancreas	Pancreas	Normal Tissue	Male	GTEX
18	Normal	GTEX-PLZ6-0726-SM-3P619	Pancreas	Pancreas	Pancreas	Normal Tissue	Male	GTEX
19	Normal	GTEX-U8XE-2026-SM-3DB8S	Pancreas	Pancreas	Pancreas	Normal Tissue	Male	GTEX
20	Normal	GTEX-132QS-0926-SM-5P9GB	Pancreas	Pancreas	Pancreas	Normal Tissue	Male	GTEX
21	Normal	GTEX-146FQ-1526-SM-5Q5CX	Pancreas	Pancreas	Pancreas	Normal Tissue	Male	GTEX
22	Normal	GTEX-12WSN-0826-SM-5GCOF	Pancreas	Pancreas	Pancreas	Normal Tissue	Male	GTEX
23	Normal	GTEX-Y3I4-0826-SM-4TT2A	Pancreas	Pancreas	Pancreas	Normal Tissue	Male	GTEX
24	Normal	GTEX-OHPM-1026-SM-3LK74	Pancreas	Pancreas	Pancreas	Normal Tissue	Male	GTEX
25	Normal	GTEX-12WSL-0426-SM-5GCNX	Pancreas	Pancreas	Pancreas	Normal Tissue	Male	GTEX
26	Normal	GTEX-P4QS-1026-SM-3NMCW	Pancreas	Pancreas	Pancreas	Normal Tissue	Male	GTEX
27	Normal	GTEX-OOBJ-1026-SM-3NB2L	Pancreas	Pancreas	Pancreas	Normal Tissue	Male	GTEX
28	Normal	GTEX-Q2AH-0926-SM-48TZK	Pancreas	Pancreas	Pancreas	Normal Tissue	Male	GTEX

29	Normal	GTEX-V955-0326-SM-4JBGV	Pancreas	Pancreas	Pancreas	Normal Tissue	Male	GTEX
30	Normal	GTEX-ZAB4-1726-SM-5HL8C	Pancreas	Pancreas	Pancreas	Normal Tissue	Male	GTEX
31	Normal	GTEX-YF7O-1326-SM-4W1ZR	Pancreas	Pancreas	Pancreas	Normal Tissue	Male	GTEX
32	Normal	GTEX-11ONC-0526-SM-5BC57	Pancreas	Pancreas	Pancreas	Normal Tissue	Male	GTEX
33	Normal	GTEX-XMK1-0326-SM-4B652	Pancreas	Pancreas	Pancreas	Normal Tissue	Male	GTEX
34	Normal	GTEX-QEL4-1326-SM-447AD	Pancreas	Pancreas	Pancreas	Normal Tissue	Male	GTEX
35	Normal	GTEX-QV44-0426-SM-4R1KF	Pancreas	Pancreas	Pancreas	Normal Tissue	Male	GTEX
36	Normal	GTEX-WFJO-0626-SM-4LVMC	Pancreas	Pancreas	Pancreas	Normal Tissue	Male	GTEX
37	Normal	GTEX-WYJK-2426-SM-4ONDQ	Pancreas	Pancreas	Pancreas	Normal Tissue	Male	GTEX
38	Normal	GTEX-144GN-0826-SM-5LU5G	Pancreas	Pancreas	Pancreas	Normal Tissue	Male	GTEX
39	Normal	GTEX-SIU7-0926-SM-4BRX1	Pancreas	Pancreas	Pancreas	Normal Tissue	Male	GTEX
40	Normal	GTEX-145MN-1426-SM-5SI9H	Pancreas	Pancreas	Pancreas	Normal Tissue	Male	GTEX
41	Normal	GTEX-Y9LG-0726-SM-4VDS3	Pancreas	Pancreas	Pancreas	Normal Tissue	Male	GTEX
42	Normal	GTEX-11LCK-0226-SM-5A5M6	Pancreas	Pancreas	Pancreas	Normal Tissue	Male	GTEX
43	Normal	GTEX-QESD-0226-SM-447BH	Pancreas	Pancreas	Pancreas	Normal Tissue	Male	GTEX
44	Normal	GTEX-WHWD-0726-SM-4OORX	Pancreas	Pancreas	Pancreas	Normal Tissue	Male	GTEX
45	Normal	GTEX-144FL-1526-SM-5Q5CA	Pancreas	Pancreas	Pancreas	Normal Tissue	Male	GTEX
46	Normal	GTEX-YB5E-0526-SM-4VDSD	Pancreas	Pancreas	Pancreas	Normal Tissue	Male	GTEX
47	Normal	GTEX-PSDG-1526-SM-48TCY	Pancreas	Pancreas	Pancreas	Normal Tissue	Male	GTEX
48	Normal	GTEX-S33H-1226-SM-4AD69	Pancreas	Pancreas	Pancreas	Normal Tissue	Male	GTEX
49	Normal	GTEX-XPVG-0326-SM-4B653	Pancreas	Pancreas	Pancreas	Normal Tissue	Male	GTEX
50	Normal	GTEX-R55D-1426-SM-48FEN	Pancreas	Pancreas	Pancreas	Normal Tissue	Male	GTEX
51	Normal	GTEX-S95S-0726-SM-4B64H	Pancreas	Pancreas	Pancreas	Normal Tissue	Male	GTEX
52	Normal	GTEX-YEC4-1326-SM-5IFHG	Pancreas	Pancreas	Pancreas	Normal Tissue	Male	GTEX
53	Normal	GTEX-ZEX8-1026-SM-4WKHE	Pancreas	Pancreas	Pancreas	Normal Tissue	Male	GTEX
54	Normal	GTEX-11EQ9-1026-SM-5H134	Pancreas	Pancreas	Pancreas	Normal Tissue	Male	GTEX
55	Normal	GTEX-WFON-0626-SM-4LVLX	Pancreas	Pancreas	Pancreas	Normal Tissue	Male	GTEX
56	Normal	GTEX-11P7K-0526-SM-5BC5I	Pancreas	Pancreas	Pancreas	Normal Tissue	Male	GTEX
57	Normal	GTEX-13FTW-0526-SM-5IFIP	Pancreas	Pancreas	Pancreas	Normal Tissue	Male	GTEX
58	Normal	GTEX-ZDYS-2526-SM-4WKGU	Pancreas	Pancreas	Pancreas	Normal Tissue	Male	GTEX

59	Normal	GTEX-ZVZP-0626-SM-59HL5	Pancreas	Pancreas	Pancreas	Normal Tissue	Male	GTEX
60	Normal	GTEX-X4LF-0326-SM-4QAS9	Pancreas	Pancreas	Pancreas	Normal Tissue	Male	GTEX
61	Normal	GTEX-YEC3-0626-SM-5IFHZ	Pancreas	Pancreas	Pancreas	Normal Tissue	Male	GTEX
62	Normal	GTEX-WHSB-0726-SM-4M1XQ	Pancreas	Pancreas	Pancreas	Normal Tissue	Male	GTEX
63	Normal	GTEX-13O61-2126-SM-5IJEO	Pancreas	Pancreas	Pancreas	Normal Tissue	Male	GTEX
64	Normal	GTEX-QLQW-0326-SM-447A8	Pancreas	Pancreas	Pancreas	Normal Tissue	Male	GTEX
65	Normal	GTEX-QV31-0226-SM-447BO	Pancreas	Pancreas	Pancreas	Normal Tissue	Male	GTEX
66	Normal	GTEX-XBED-0226-SM-47JY8	Pancreas	Pancreas	Pancreas	Normal Tissue	Male	GTEX
67	Normal	GTEX-Y8E4-1326-SM-5IFIY	Pancreas	Pancreas	Pancreas	Normal Tissue	Male	GTEX
68	Normal	GTEX-ZLFU-0726-SM-57WF6	Pancreas	Pancreas	Pancreas	Normal Tissue	Male	GTEX
69	Normal	GTEX-S3XE-0526-SM-4AD4G	Pancreas	Pancreas	Pancreas	Normal Tissue	Male	GTEX
70	Normal	GTEX-SNOS-0926-SM-4DM7A	Pancreas	Pancreas	Pancreas	Normal Tissue	Male	GTEX
71	Normal	GTEX-XQ8I-1926-SM-4BOOK	Pancreas	Pancreas	Pancreas	Normal Tissue	Male	GTEX
72	Normal	GTEX-YEC3-0626-SM-4YCFE	Pancreas	Pancreas	Pancreas	Normal Tissue	Male	GTEX
73	Normal	GTEX-QDVJ-1226-SM-48U1V	Pancreas	Pancreas	Pancreas	Normal Tissue	Male	GTEX
74	Normal	GTEX-X5EB-0526-SM-46MVP	Pancreas	Pancreas	Pancreas	Normal Tissue	Male	GTEX
75	Normal	GTEX-Q2AI-0426-SM-48U13	Pancreas	Pancreas	Pancreas	Normal Tissue	Male	GTEX
76	Normal	GTEX-WHPG-0326-SM-4M1XV	Pancreas	Pancreas	Pancreas	Normal Tissue	Male	GTEX
77	Normal	GTEX-SUCS-1426-SM-4DM5W	Pancreas	Pancreas	Pancreas	Normal Tissue	Male	GTEX
78	Normal	GTEX-ZT9W-0926-SM-57WFS	Pancreas	Pancreas	Pancreas	Normal Tissue	Male	GTEX
79	Normal	GTEX-ZDTT-1126-SM-4WKFW	Pancreas	Pancreas	Pancreas	Normal Tissue	Male	GTEX
80	Normal	GTEX-14ICL-1126-SM-5S2RE	Pancreas	Pancreas	Pancreas	Normal Tissue	Male	GTEX
81	Normal	GTEX-OOBK-1026-SM-48TC2	Pancreas	Pancreas	Pancreas	Normal Tissue	Male	GTEX
82	Normal	GTEX-11TT1-0326-SM-5LUAY	Pancreas	Pancreas	Pancreas	Normal Tissue	Male	GTEX
83	Normal	GTEX-T8EM-0826-SM-4DM76	Pancreas	Pancreas	Pancreas	Normal Tissue	Male	GTEX
84	Normal	GTEX-ZG7Y-0326-SM-4WWEY	Pancreas	Pancreas	Pancreas	Normal Tissue	Male	GTEX
85	Normal	GTEX-111CU-0526-SM-5EGHK	Pancreas	Pancreas	Pancreas	Normal Tissue	Male	GTEX
86	Normal	GTEX-111YS-1226-SM-5EGGJ	Pancreas	Pancreas	Pancreas	Normal Tissue	Male	GTEX
87	Normal	GTEX-117YX-0226-SM-5EGH6	Pancreas	Pancreas	Pancreas	Normal Tissue	Male	GTEX
88	Normal	GTEX-X3Y1-0726-SM-3P5YU	Pancreas	Pancreas	Pancreas	Normal Tissue	Male	GTEX

89	Normal	GTEX-TKQ2-0426-SM-4DXUO	Pancreas	Pancreas	Pancreas	Normal Tissue	Male	GTEX
90	Normal	GTEX-RM2N-0326-SM-48FD8	Pancreas	Pancreas	Pancreas	Normal Tissue	Male	GTEX
91	Normal	GTEX-WFG8-0326-SM-4LVN4	Pancreas	Pancreas	Pancreas	Normal Tissue	Male	GTEX
92	Normal	GTEX-WH7G-0826-SM-4LVMR	Pancreas	Pancreas	Pancreas	Normal Tissue	Male	GTEX
93	Normal	GTEX-1339X-1026-SM-5IFH5	Pancreas	Pancreas	Pancreas	Normal Tissue	Male	GTEX
94	Normal	GTEX-U8XE-2026-SM-5CHQF	Pancreas	Pancreas	Pancreas	Normal Tissue	Male	GTEX
95	Normal	GTEX-146FH-1826-SM-5QGQ7	Pancreas	Pancreas	Pancreas	Normal Tissue	Female	GTEX
96	Normal	GTEX-XYKS-1226-SM-4BRVI	Pancreas	Pancreas	Pancreas	Normal Tissue	Female	GTEX
97	Normal	GTEX-ZF3C-2026-SM-4WWB5	Pancreas	Pancreas	Pancreas	Normal Tissue	Female	GTEX
98	Normal	GTEX-11ILO-1526-SM-5A5KZ	Pancreas	Pancreas	Pancreas	Normal Tissue	Female	GTEX
99	Normal	GTEX-ZYWO-1326-SM-5SI8X	Pancreas	Pancreas	Pancreas	Normal Tissue	Female	GTEX
100	Normal	GTEX-Y3IK-0426-SM-4WWE2	Pancreas	Pancreas	Pancreas	Normal Tissue	Female	GTEX
101	Normal	GTEX-WYVS-0926-SM-4SOJV	Pancreas	Pancreas	Pancreas	Normal Tissue	Female	GTEX
102	Normal	GTEX-R53T-0426-SM-48FEM	Pancreas	Pancreas	Pancreas	Normal Tissue	Female	GTEX
103	Normal	GTEX-132AR-1826-SM-5EGHR	Pancreas	Pancreas	Pancreas	Normal Tissue	Female	GTEX
104	Normal	GTEX-ZTPG-1026-SM-5DUWP	Pancreas	Pancreas	Pancreas	Normal Tissue	Female	GTEX
105	Normal	GTEX-QCQG-0426-SM-48U29	Pancreas	Pancreas	Pancreas	Normal Tissue	Female	GTEX
106	Normal	GTEX-13X6H-0626-SM-5LU53	Pancreas	Pancreas	Pancreas	Normal Tissue	Female	GTEX
107	Normal	GTEX-S32W-0826-SM-4AD5Z	Pancreas	Pancreas	Pancreas	Normal Tissue	Female	GTEX
108	Normal	GTEX-ZYY3-0826-SM-5E44R	Pancreas	Pancreas	Pancreas	Normal Tissue	Female	GTEX
109	Normal	GTEX-13D11-2226-SM-5IFEO	Pancreas	Pancreas	Pancreas	Normal Tissue	Female	GTEX
110	Normal	GTEX-OHPL-1026-SM-3MJGI	Pancreas	Pancreas	Pancreas	Normal Tissue	Female	GTEX
111	Normal	GTEX-14AS3-0326-SM-5Q5DB	Pancreas	Pancreas	Pancreas	Normal Tissue	Female	GTEX
112	Normal	GTEX-S4P3-0626-SM-4AD59	Pancreas	Pancreas	Pancreas	Normal Tissue	Female	GTEX
113	Normal	GTEX-Y5LM-0526-SM-4V6G3	Pancreas	Pancreas	Pancreas	Normal Tissue	Female	GTEX
114	Normal	GTEX-P4PP-1026-SM-3NM9O	Pancreas	Pancreas	Pancreas	Normal Tissue	Female	GTEX
115	Normal	GTEX-13N11-0226-SM-5KM3C	Pancreas	Pancreas	Pancreas	Normal Tissue	Female	GTEX
116	Normal	GTEX-N7MT-1626-SM-3LK71	Pancreas	Pancreas	Pancreas	Normal Tissue	Female	GTEX
117	Normal	GTEX-146FR-0126-SM-5Q5F3	Pancreas	Pancreas	Pancreas	Normal Tissue	Female	GTEX
118	Normal	GTEX-WXYG-0826-SM-4ONC7	Pancreas	Pancreas	Pancreas	Normal Tissue	Female	GTEX

119	Normal	GTEX-13U4I-1526-SM-5IFFF	Pancreas	Pancreas	Pancreas	Normal Tissue	Female	GTEX
120	Normal	GTEX-1122O-0726-SM-5GIEV	Pancreas	Pancreas	Pancreas	Normal Tissue	Female	GTEX
121	Normal	GTEX-11I78-0626-SM-5A5LZ	Pancreas	Pancreas	Pancreas	Normal Tissue	Female	GTEX
122	Normal	GTEX-ZZPU-0726-SM-5N9C8	Pancreas	Pancreas	Pancreas	Normal Tissue	Female	GTEX
123	Normal	GTEX-PX3G-1026-SM-48TZW	Pancreas	Pancreas	Pancreas	Normal Tissue	Female	GTEX
124	Normal	GTEX-11XUK-0626-SM-5N9ES	Pancreas	Pancreas	Pancreas	Normal Tissue	Female	GTEX
125	Normal	GTEX-ZC5H-0826-SM-5N9FH	Pancreas	Pancreas	Pancreas	Normal Tissue	Female	GTEX
126	Normal	GTEX-ZPIC-0926-SM-4WWFK	Pancreas	Pancreas	Pancreas	Normal Tissue	Female	GTEX
127	Normal	GTEX-ZYFG-0826-SM-5BC5T	Pancreas	Pancreas	Pancreas	Normal Tissue	Female	GTEX
128	Normal	GTEX-13VXT-1226-SM-5LU3M	Pancreas	Pancreas	Pancreas	Normal Tissue	Female	GTEX
129	Normal	GTEX-13FH7-1426-SM-5IFIC	Pancreas	Pancreas	Pancreas	Normal Tissue	Female	GTEX
130	Normal	GTEX-Y8LW-1026-SM-5IFJY	Pancreas	Pancreas	Pancreas	Normal Tissue	Female	GTEX
131	Normal	GTEX-RWS6-1126-SM-47JXC	Pancreas	Pancreas	Pancreas	Normal Tissue	Female	GTEX
132	Normal	GTEX-1399U-0926-SM-5IFHA	Pancreas	Pancreas	Pancreas	Normal Tissue	Female	GTEX
133	Normal	GTEX-13CF3-1026-SM-5LZWY	Pancreas	Pancreas	Pancreas	Normal Tissue	Female	GTEX
134	Normal	GTEX-13W3W-1026-SM-5IFG4	Pancreas	Pancreas	Pancreas	Normal Tissue	Female	GTEX
135	Normal	GTEX-PWOO-0626-SM-48TZH	Pancreas	Pancreas	Pancreas	Normal Tissue	Female	GTEX
136	Normal	GTEX-ZP4G-0426-SM-4YCER	Pancreas	Pancreas	Pancreas	Normal Tissue	Female	GTEX
137	Normal	GTEX-13FTX-1226-SM-5IFGN	Pancreas	Pancreas	Pancreas	Normal Tissue	Female	GTEX
138	Normal	GTEX-UJHI-0626-SM-3DB8T	Pancreas	Pancreas	Pancreas	Normal Tissue	Female	GTEX
139	Normal	GTEX-ZF2S-0426-SM-4WKGP	Pancreas	Pancreas	Pancreas	Normal Tissue	Female	GTEX
140	Normal	GTEX-131XG-1426-SM-5GCMO	Pancreas	Pancreas	Pancreas	Normal Tissue	Female	GTEX
141	Normal	GTEX-ZVT2-2026-SM-5NQ8Q	Pancreas	Pancreas	Pancreas	Normal Tissue	Female	GTEX
142	Normal	GTEX-12WSD-1626-SM-5GCNR	Pancreas	Pancreas	Pancreas	Normal Tissue	Female	GTEX
143	Normal	GTEX-12WSK-0226-SM-5BC62	Pancreas	Pancreas	Pancreas	Normal Tissue	Female	GTEX
144	Normal	GTEX-X15G-0726-SM-4PQZ5	Pancreas	Pancreas	Pancreas	Normal Tissue	Female	GTEX
145	Normal	GTEX-ZLWG-0326-SM-4WWC7	Pancreas	Pancreas	Pancreas	Normal Tissue	Female	GTEX
146	Normal	GTEX-13PL7-2226-SM-5L3IC	Pancreas	Pancreas	Pancreas	Normal Tissue	Female	GTEX
147	Normal	GTEX-WI4N-1826-SM-4OOSF	Pancreas	Pancreas	Pancreas	Normal Tissue	Female	GTEX
148	Normal	GTEX-1128S-0826-SM-5GZZI	Pancreas	Pancreas	Pancreas	Normal Tissue	Female	GTEX

149	Normal	GTEX-11VI4-0426-SM-5EGHZ	Pancreas	Pancreas	Pancreas	Normal Tissue	Female	GTEX
150	Normal	GTEX-Y5V5-1026-SM-5LUAH	Pancreas	Pancreas	Pancreas	Normal Tissue	Female	GTEX
151	Normal	GTEX-R55G-0326-SM-48FDM	Pancreas	Pancreas	Pancreas	Normal Tissue	Female	GTEX
152	Normal	GTEX-13OVI-0526-SM-5IFFQ	Pancreas	Pancreas	Pancreas	Normal Tissue	Female	GTEX
153	Normal	GTEX-1399S-0326-SM-5IFFS	Pancreas	Pancreas	Pancreas	Normal Tissue	Female	GTEX
154	Normal	GTEX-ZF29-1126-SM-4WKGO	Pancreas	Pancreas	Pancreas	Normal Tissue	Female	GTEX
155	Normal	GTEX-11DXX-0926-SM-5H112	Pancreas	Pancreas	Pancreas	Normal Tissue	Female	GTEX
156	Normal	GTEX-ZAK1-2326-SM-5CVMY	Pancreas	Pancreas	Pancreas	Normal Tissue	Female	GTEX
157	Normal	GTEX-145ME-0326-SM-5S2QO	Pancreas	Pancreas	Pancreas	Normal Tissue	Female	GTEX
158	Normal	GTEX-SE5C-0326-SM-4BRWX	Pancreas	Pancreas	Pancreas	Normal Tissue	Female	GTEX
159	Normal	GTEX-YB5K-1826-SM-5IFJC	Pancreas	Pancreas	Pancreas	Normal Tissue	Female	GTEX
160	Normal	GTEX-1211K-1126-SM-5EGGB	Pancreas	Pancreas	Pancreas	Normal Tissue	Female	GTEX
161	Normal	GTEX-TMMY-1326-SM-4DXU9	Pancreas	Pancreas	Pancreas	Normal Tissue	Female	GTEX
162	Normal	GTEX-W5WG-0826-SM-4RGNE	Pancreas	Pancreas	Pancreas	Normal Tissue	Female	GTEX
163	Normal	GTEX-13SLX-1326-SM-5S2QS	Pancreas	Pancreas	Pancreas	Normal Tissue	Female	GTEX
164	Normal	GTEX-XUW1-1726-SM-4BOOZ	Pancreas	Pancreas	Pancreas	Normal Tissue	Female	GTEX
165	Normal	GTEX-12WSG-1026-SM-5EGII	Pancreas	Pancreas	Pancreas	Normal Tissue	Female	GTEX
166	Normal	GTEX-WRHK-0226-SM-4MVOH	Pancreas	Pancreas	Pancreas	Normal Tissue	Female	GTEX
167	Normal	GTEX-W5X1-0226-SM-5CHTO	Pancreas	Pancreas	Pancreas	Normal Tissue	Female	GTEX
168	Normal	TCGA-YB-A89D-11	Pancreatic Adenocarcinoma	Pancreatic Adenocarcinoma	Pancreas	Solid Tissue Normal	Male	TCGA
169	Normal	TCGA-HV-A5A3-11	Pancreatic Adenocarcinoma	Pancreatic Adenocarcinoma	Pancreas	Solid Tissue Normal	Male	TCGA
170	Normal	TCGA-H6-A45N-11	Pancreatic Adenocarcinoma	Pancreatic Adenocarcinoma	Pancreas	Solid Tissue Normal	Female	TCGA
171	Normal	TCGA-H6-8124-11	Pancreatic Adenocarcinoma	Pancreatic Adenocarcinoma	Pancreas	Solid Tissue Normal	Female	TCGA
172	Tumor	TCGA-FB-AAQ6-01	Pancreatic Adenocarcinoma	Pancreatic Adenocarcinoma	Pancreas	Primary Tumor	Male	TCGA
173	Tumor	TCGA-2L-AAQL-01	Pancreatic Adenocarcinoma	Pancreatic Adenocarcinoma	Pancreas	Primary Tumor	Male	TCGA
174	Tumor	TCGA-HZ-8005-01	Pancreatic Adenocarcinoma	Pancreatic Adenocarcinoma	Pancreas	Primary Tumor	Male	TCGA
175	Tumor	TCGA-HZ-A8P1-01	Pancreatic Adenocarcinoma	Pancreatic Adenocarcinoma	Pancreas	Primary Tumor	Male	TCGA

176	Tumor	TCGA-IB-A7M4-01	Pancreatic Adenocarcinoma	Pancreatic Adenocarcinoma	Pancreas	Primary Tumor	Male	TCGA
177	Tumor	TCGA-IB-7654-01	Pancreatic Adenocarcinoma	Pancreatic Adenocarcinoma	Pancreas	Primary Tumor	Male	TCGA
178	Tumor	TCGA-IB-7886-01	Pancreatic Adenocarcinoma	Pancreatic Adenocarcinoma	Pancreas	Primary Tumor	Male	TCGA
179	Tumor	TCGA-F2-A7TX-01	Pancreatic Adenocarcinoma	Pancreatic Adenocarcinoma	Pancreas	Primary Tumor	Male	TCGA
180	Tumor	TCGA-HZ-7289-01	Pancreatic Adenocarcinoma	Pancreatic Adenocarcinoma	Pancreas	Primary Tumor	Male	TCGA
181	Tumor	TCGA-HZ-A49I-01	Pancreatic Adenocarcinoma	Pancreatic Adenocarcinoma	Pancreas	Primary Tumor	Male	TCGA
182	Tumor	TCGA-HZ-A77P-01	Pancreatic Adenocarcinoma	Pancreatic Adenocarcinoma	Pancreas	Primary Tumor	Male	TCGA
183	Tumor	TCGA-IB-A5SP-01	Pancreatic Adenocarcinoma	Pancreatic Adenocarcinoma	Pancreas	Primary Tumor	Male	TCGA
184	Tumor	TCGA-2L-AAQA-01	Pancreatic Adenocarcinoma	Pancreatic Adenocarcinoma	Pancreas	Primary Tumor	Male	TCGA
185	Tumor	TCGA-F2-A8YN-01	Pancreatic Adenocarcinoma	Pancreatic Adenocarcinoma	Pancreas	Primary Tumor	Male	TCGA
186	Tumor	TCGA-HZ-A8P0-01	Pancreatic Adenocarcinoma	Pancreatic Adenocarcinoma	Pancreas	Primary Tumor	Male	TCGA
187	Tumor	TCGA-IB-AAUM-01	Pancreatic Adenocarcinoma	Pancreatic Adenocarcinoma	Pancreas	Primary Tumor	Male	TCGA
188	Tumor	TCGA-2J-AAB6-01	Pancreatic Adenocarcinoma	Pancreatic Adenocarcinoma	Pancreas	Primary Tumor	Male	TCGA
189	Tumor	TCGA-3A-A9J0-01	Pancreatic Adenocarcinoma	Pancreatic Adenocarcinoma	Pancreas	Primary Tumor	Male	TCGA
190	Tumor	TCGA-HZ-A4BH-01	Pancreatic Adenocarcinoma	Pancreatic Adenocarcinoma	Pancreas	Primary Tumor	Male	TCGA
191	Tumor	TCGA-OE-A75W-01	Pancreatic Adenocarcinoma	Pancreatic Adenocarcinoma	Pancreas	Primary Tumor	Male	TCGA
192	Tumor	TCGA-2J-AABV-01	Pancreatic Adenocarcinoma	Pancreatic Adenocarcinoma	Pancreas	Primary Tumor	Male	TCGA
193	Tumor	TCGA-FB-A5VM-01	Pancreatic Adenocarcinoma	Pancreatic Adenocarcinoma	Pancreas	Primary Tumor	Male	TCGA
194	Tumor	TCGA-2J-AABE-01	Pancreatic Adenocarcinoma	Pancreatic Adenocarcinoma	Pancreas	Primary Tumor	Male	TCGA
195	Tumor	TCGA-2J-AABF-01	Pancreatic Adenocarcinoma	Pancreatic Adenocarcinoma	Pancreas	Primary Tumor	Male	TCGA
196	Tumor	TCGA-HZ-8519-01	Pancreatic Adenocarcinoma	Pancreatic Adenocarcinoma	Pancreas	Primary Tumor	Male	TCGA
197	Tumor	TCGA-IB-7890-01	Pancreatic Adenocarcinoma	Pancreatic Adenocarcinoma	Pancreas	Primary Tumor	Male	TCGA

198	Tumor	TCGA-US-A77E-01	Pancreatic Adenocarcinoma	Pancreatic Adenocarcinoma	Pancreas	Primary Tumor	Male	TCGA
199	Tumor	TCGA-HV-A7OP-01	Pancreatic Adenocarcinoma	Pancreatic Adenocarcinoma	Pancreas	Primary Tumor	Male	TCGA
200	Tumor	TCGA-HZ-7918-01	Pancreatic Adenocarcinoma	Pancreatic Adenocarcinoma	Pancreas	Primary Tumor	Male	TCGA
201	Tumor	TCGA-HZ-A4BK-01	Pancreatic Adenocarcinoma	Pancreatic Adenocarcinoma	Pancreas	Primary Tumor	Male	TCGA
202	Tumor	TCGA-2J-AAB8-01	Pancreatic Adenocarcinoma	Pancreatic Adenocarcinoma	Pancreas	Primary Tumor	Male	TCGA
203	Tumor	TCGA-2J-AABK-01	Pancreatic Adenocarcinoma	Pancreatic Adenocarcinoma	Pancreas	Primary Tumor	Male	TCGA
204	Tumor	TCGA-3E-AAAZ-01	Pancreatic Adenocarcinoma	Pancreatic Adenocarcinoma	Pancreas	Primary Tumor	Male	TCGA
205	Tumor	TCGA-FB-AAPP-01	Pancreatic Adenocarcinoma	Pancreatic Adenocarcinoma	Pancreas	Primary Tumor	Male	TCGA
206	Tumor	TCGA-FB-AAPY-01	Pancreatic Adenocarcinoma	Pancreatic Adenocarcinoma	Pancreas	Primary Tumor	Male	TCGA
207	Tumor	TCGA-HZ-7920-01	Pancreatic Adenocarcinoma	Pancreatic Adenocarcinoma	Pancreas	Primary Tumor	Male	TCGA
208	Tumor	TCGA-IB-A5SO-01	Pancreatic Adenocarcinoma	Pancreatic Adenocarcinoma	Pancreas	Primary Tumor	Male	TCGA
209	Tumor	TCGA-F2-6880-01	Pancreatic Adenocarcinoma	Pancreatic Adenocarcinoma	Pancreas	Primary Tumor	Male	TCGA
210	Tumor	TCGA-HV-A7OL-01	Pancreatic Adenocarcinoma	Pancreatic Adenocarcinoma	Pancreas	Primary Tumor	Male	TCGA
211	Tumor	TCGA-HZ-A9TJ-01	Pancreatic Adenocarcinoma	Pancreatic Adenocarcinoma	Pancreas	Primary Tumor	Male	TCGA
212	Tumor	TCGA-HZ-8001-01	Pancreatic Adenocarcinoma	Pancreatic Adenocarcinoma	Pancreas	Primary Tumor	Male	TCGA
213	Tumor	TCGA-FB-AAQ0-01	Pancreatic Adenocarcinoma	Pancreatic Adenocarcinoma	Pancreas	Primary Tumor	Male	TCGA
214	Tumor	TCGA-IB-AAUP-01	Pancreatic Adenocarcinoma	Pancreatic Adenocarcinoma	Pancreas	Primary Tumor	Male	TCGA
215	Tumor	TCGA-3A-A9I9-01	Pancreatic Adenocarcinoma	Pancreatic Adenocarcinoma	Pancreas	Primary Tumor	Male	TCGA
216	Tumor	TCGA-3A-A9IS-01	Pancreatic Adenocarcinoma	Pancreatic Adenocarcinoma	Pancreas	Primary Tumor	Male	TCGA
217	Tumor	TCGA-3E-AAAY-01	Pancreatic Adenocarcinoma	Pancreatic Adenocarcinoma	Pancreas	Primary Tumor	Male	TCGA
218	Tumor	TCGA-IB-AAUR-01	Pancreatic Adenocarcinoma	Pancreatic Adenocarcinoma	Pancreas	Primary Tumor	Male	TCGA
219	Tumor	TCGA-S4-A8RM-01	Pancreatic Adenocarcinoma	Pancreatic Adenocarcinoma	Pancreas	Primary Tumor	Male	TCGA

220	Tumor	TCGA-XN-A8T3-01	Pancreatic Adenocarcinoma	Pancreatic Adenocarcinoma	Pancreas	Primary Tumor	Male	TCGA
221	Tumor	TCGA-2L-AAQI-01	Pancreatic Adenocarcinoma	Pancreatic Adenocarcinoma	Pancreas	Primary Tumor	Male	TCGA
222	Tumor	TCGA-HZ-7925-01	Pancreatic Adenocarcinoma	Pancreatic Adenocarcinoma	Pancreas	Primary Tumor	Male	TCGA
223	Tumor	TCGA-2J-AAB1-01	Pancreatic Adenocarcinoma	Pancreatic Adenocarcinoma	Pancreas	Primary Tumor	Male	TCGA
224	Tumor	TCGA-3A-A9IJ-01	Pancreatic Adenocarcinoma	Pancreatic Adenocarcinoma	Pancreas	Primary Tumor	Male	TCGA
225	Tumor	TCGA-3A-A9IU-01	Pancreatic Adenocarcinoma	Pancreatic Adenocarcinoma	Pancreas	Primary Tumor	Male	TCGA
226	Tumor	TCGA-F2-A44H-01	Pancreatic Adenocarcinoma	Pancreatic Adenocarcinoma	Pancreas	Primary Tumor	Male	TCGA
227	Tumor	TCGA-FB-AAPQ-01	Pancreatic Adenocarcinoma	Pancreatic Adenocarcinoma	Pancreas	Primary Tumor	Male	TCGA
228	Tumor	TCGA-HZ-7923-01	Pancreatic Adenocarcinoma	Pancreatic Adenocarcinoma	Pancreas	Primary Tumor	Male	TCGA
229	Tumor	TCGA-IB-A6UG-01	Pancreatic Adenocarcinoma	Pancreatic Adenocarcinoma	Pancreas	Primary Tumor	Male	TCGA
230	Tumor	TCGA-IB-AAUT-01	Pancreatic Adenocarcinoma	Pancreatic Adenocarcinoma	Pancreas	Primary Tumor	Male	TCGA
231	Tumor	TCGA-F2-7276-01	Pancreatic Adenocarcinoma	Pancreatic Adenocarcinoma	Pancreas	Primary Tumor	Male	TCGA
232	Tumor	TCGA-IB-7893-01	Pancreatic Adenocarcinoma	Pancreatic Adenocarcinoma	Pancreas	Primary Tumor	Male	TCGA
233	Tumor	TCGA-US-A77G-01	Pancreatic Adenocarcinoma	Pancreatic Adenocarcinoma	Pancreas	Primary Tumor	Male	TCGA
234	Tumor	TCGA-IB-A6UF-01	Pancreatic Adenocarcinoma	Pancreatic Adenocarcinoma	Pancreas	Primary Tumor	Male	TCGA
235	Tumor	TCGA-2J-AABH-01	Pancreatic Adenocarcinoma	Pancreatic Adenocarcinoma	Pancreas	Primary Tumor	Male	TCGA
236	Tumor	TCGA-HZ-8002-01	Pancreatic Adenocarcinoma	Pancreatic Adenocarcinoma	Pancreas	Primary Tumor	Male	TCGA
237	Tumor	TCGA-US-A776-01	Pancreatic Adenocarcinoma	Pancreatic Adenocarcinoma	Pancreas	Primary Tumor	Male	TCGA
238	Tumor	TCGA-IB-7646-01	Pancreatic Adenocarcinoma	Pancreatic Adenocarcinoma	Pancreas	Primary Tumor	Male	TCGA
239	Tumor	TCGA-IB-8127-01	Pancreatic Adenocarcinoma	Pancreatic Adenocarcinoma	Pancreas	Primary Tumor	Male	TCGA
240	Tumor	TCGA-YB-A89D-01	Pancreatic Adenocarcinoma	Pancreatic Adenocarcinoma	Pancreas	Primary Tumor	Male	TCGA
241	Tumor	TCGA-Q3-A5QY-01	Pancreatic Adenocarcinoma	Pancreatic Adenocarcinoma	Pancreas	Primary Tumor	Male	TCGA

242	Tumor	TCGA-3A-A9I5-01	Pancreatic Adenocarcinoma	Pancreatic Adenocarcinoma	Pancreas	Primary Tumor	Male	TCGA
243	Tumor	TCGA-F2-6879-01	Pancreatic Adenocarcinoma	Pancreatic Adenocarcinoma	Pancreas	Primary Tumor	Male	TCGA
244	Tumor	TCGA-HZ-7926-01	Pancreatic Adenocarcinoma	Pancreatic Adenocarcinoma	Pancreas	Primary Tumor	Male	TCGA
245	Tumor	TCGA-IB-A7LX-01	Pancreatic Adenocarcinoma	Pancreatic Adenocarcinoma	Pancreas	Primary Tumor	Male	TCGA
246	Tumor	TCGA-2J-AABU-01	Pancreatic Adenocarcinoma	Pancreatic Adenocarcinoma	Pancreas	Primary Tumor	Male	TCGA
247	Tumor	TCGA-2L-AAQE-01	Pancreatic Adenocarcinoma	Pancreatic Adenocarcinoma	Pancreas	Primary Tumor	Male	TCGA
248	Tumor	TCGA-XD-AAUL-01	Pancreatic Adenocarcinoma	Pancreatic Adenocarcinoma	Pancreas	Primary Tumor	Male	TCGA
249	Tumor	TCGA-2J-AABA-01	Pancreatic Adenocarcinoma	Pancreatic Adenocarcinoma	Pancreas	Primary Tumor	Male	TCGA
250	Tumor	TCGA-3A-A9IO-01	Pancreatic Adenocarcinoma	Pancreatic Adenocarcinoma	Pancreas	Primary Tumor	Male	TCGA
251	Tumor	TCGA-F2-7273-01	Pancreatic Adenocarcinoma	Pancreatic Adenocarcinoma	Pancreas	Primary Tumor	Male	TCGA
252	Tumor	TCGA-FB-A4P6-01	Pancreatic Adenocarcinoma	Pancreatic Adenocarcinoma	Pancreas	Primary Tumor	Male	TCGA
253	Tumor	TCGA-FB-AAPZ-01	Pancreatic Adenocarcinoma	Pancreatic Adenocarcinoma	Pancreas	Primary Tumor	Male	TCGA
254	Tumor	TCGA-H8-A6C1-01	Pancreatic Adenocarcinoma	Pancreatic Adenocarcinoma	Pancreas	Primary Tumor	Male	TCGA
255	Tumor	TCGA-2L-AAQM-01	Pancreatic Adenocarcinoma	Pancreatic Adenocarcinoma	Pancreas	Primary Tumor	Male	TCGA
256	Tumor	TCGA-HV-A5A3-01	Pancreatic Adenocarcinoma	Pancreatic Adenocarcinoma	Pancreas	Primary Tumor	Male	TCGA
257	Tumor	TCGA-HV-AA8V-01	Pancreatic Adenocarcinoma	Pancreatic Adenocarcinoma	Pancreas	Primary Tumor	Male	TCGA
258	Tumor	TCGA-IB-AAUQ-01	Pancreatic Adenocarcinoma	Pancreatic Adenocarcinoma	Pancreas	Primary Tumor	Male	TCGA
259	Tumor	TCGA-FB-AAQ1-01	Pancreatic Adenocarcinoma	Pancreatic Adenocarcinoma	Pancreas	Primary Tumor	Male	TCGA
260	Tumor	TCGA-IB-AAUV-01	Pancreatic Adenocarcinoma	Pancreatic Adenocarcinoma	Pancreas	Primary Tumor	Male	TCGA
261	Tumor	TCGA-2J-AAB4-01	Pancreatic Adenocarcinoma	Pancreatic Adenocarcinoma	Pancreas	Primary Tumor	Male	TCGA
262	Tumor	TCGA-FB-A7DR-01	Pancreatic Adenocarcinoma	Pancreatic Adenocarcinoma	Pancreas	Primary Tumor	Male	TCGA
263	Tumor	TCGA-3A-A9IZ-01	Pancreatic Adenocarcinoma	Pancreatic Adenocarcinoma	Pancreas	Primary Tumor	Male	TCGA

264	Tumor	TCGA-3A-A9I7-01	Pancreatic Adenocarcinoma	Pancreatic Adenocarcinoma	Pancreas	Primary Tumor	Male	TCGA
265	Tumor	TCGA-2J-AABO-01	Pancreatic Adenocarcinoma	Pancreatic Adenocarcinoma	Pancreas	Primary Tumor	Male	TCGA
266	Tumor	TCGA-RB-AA9M-01	Pancreatic Adenocarcinoma	Pancreatic Adenocarcinoma	Pancreas	Primary Tumor	Male	TCGA
267	Tumor	TCGA-IB-7647-01	Pancreatic Adenocarcinoma	Pancreatic Adenocarcinoma	Pancreas	Primary Tumor	Male	TCGA
268	Tumor	TCGA-3A-A9IX-01	Pancreatic Adenocarcinoma	Pancreatic Adenocarcinoma	Pancreas	Primary Tumor	Male	TCGA
269	Tumor	TCGA-IB-AAUU-01	Pancreatic Adenocarcinoma	Pancreatic Adenocarcinoma	Pancreas	Primary Tumor	Male	TCGA
270	Tumor	TCGA-H6-A45N-01	Pancreatic Adenocarcinoma	Pancreatic Adenocarcinoma	Pancreas	Primary Tumor	Female	TCGA
271	Tumor	TCGA-IB-7889-01	Pancreatic Adenocarcinoma	Pancreatic Adenocarcinoma	Pancreas	Primary Tumor	Female	TCGA
272	Tumor	TCGA-HZ-8638-01	Pancreatic Adenocarcinoma	Pancreatic Adenocarcinoma	Pancreas	Primary Tumor	Female	TCGA
273	Tumor	TCGA-IB-AAUS-01	Pancreatic Adenocarcinoma	Pancreatic Adenocarcinoma	Pancreas	Primary Tumor	Female	TCGA
274	Tumor	TCGA-FB-AAQ2-01	Pancreatic Adenocarcinoma	Pancreatic Adenocarcinoma	Pancreas	Primary Tumor	Female	TCGA
275	Tumor	TCGA-RB-A7B8-01	Pancreatic Adenocarcinoma	Pancreatic Adenocarcinoma	Pancreas	Primary Tumor	Female	TCGA
276	Tumor	TCGA-US-A77J-01	Pancreatic Adenocarcinoma	Pancreatic Adenocarcinoma	Pancreas	Primary Tumor	Female	TCGA
277	Tumor	TCGA-F2-A44G-01	Pancreatic Adenocarcinoma	Pancreatic Adenocarcinoma	Pancreas	Primary Tumor	Female	TCGA
278	Tumor	TCGA-IB-8126-01	Pancreatic Adenocarcinoma	Pancreatic Adenocarcinoma	Pancreas	Primary Tumor	Female	TCGA
279	Tumor	TCGA-IB-7885-01	Pancreatic Adenocarcinoma	Pancreatic Adenocarcinoma	Pancreas	Primary Tumor	Female	TCGA
280	Tumor	TCGA-HZ-A77O-01	Pancreatic Adenocarcinoma	Pancreatic Adenocarcinoma	Pancreas	Primary Tumor	Female	TCGA
281	Tumor	TCGA-S4-A8RP-01	Pancreatic Adenocarcinoma	Pancreatic Adenocarcinoma	Pancreas	Primary Tumor	Female	TCGA
282	Tumor	TCGA-HZ-8637-01	Pancreatic Adenocarcinoma	Pancreatic Adenocarcinoma	Pancreas	Primary Tumor	Female	TCGA
283	Tumor	TCGA-US-A774-01	Pancreatic Adenocarcinoma	Pancreatic Adenocarcinoma	Pancreas	Primary Tumor	Female	TCGA
284	Tumor	TCGA-HV-AA8X-01	Pancreatic Adenocarcinoma	Pancreatic Adenocarcinoma	Pancreas	Primary Tumor	Female	TCGA
285	Tumor	TCGA-S4-A8RO-01	Pancreatic Adenocarcinoma	Pancreatic Adenocarcinoma	Pancreas	Primary Tumor	Female	TCGA

286	Tumor	TCGA-IB-AAUN-01	Pancreatic Adenocarcinoma	Pancreatic Adenocarcinoma	Pancreas	Primary Tumor	Female	TCGA
287	Tumor	TCGA-LB-A7SX-01	Pancreatic Adenocarcinoma	Pancreatic Adenocarcinoma	Pancreas	Primary Tumor	Female	TCGA
288	Tumor	TCGA-Z5-AAPL-01	Pancreatic Adenocarcinoma	Pancreatic Adenocarcinoma	Pancreas	Primary Tumor	Female	TCGA
289	Tumor	TCGA-IB-7649-01	Pancreatic Adenocarcinoma	Pancreatic Adenocarcinoma	Pancreas	Primary Tumor	Female	TCGA
290	Tumor	TCGA-YH-A8SY-01	Pancreatic Adenocarcinoma	Pancreatic Adenocarcinoma	Pancreas	Primary Tumor	Female	TCGA
291	Tumor	TCGA-2J-AABT-01	Pancreatic Adenocarcinoma	Pancreatic Adenocarcinoma	Pancreas	Primary Tumor	Female	TCGA
292	Tumor	TCGA-FB-A545-01	Pancreatic Adenocarcinoma	Pancreatic Adenocarcinoma	Pancreas	Primary Tumor	Female	TCGA
293	Tumor	TCGA-HV-A5A4-01	Pancreatic Adenocarcinoma	Pancreatic Adenocarcinoma	Pancreas	Primary Tumor	Female	TCGA
294	Tumor	TCGA-FB-A78T-01	Pancreatic Adenocarcinoma	Pancreatic Adenocarcinoma	Pancreas	Primary Tumor	Female	TCGA
295	Tumor	TCGA-2J-AAB9-01	Pancreatic Adenocarcinoma	Pancreatic Adenocarcinoma	Pancreas	Primary Tumor	Female	TCGA
296	Tumor	TCGA-3A-A9IB-01	Pancreatic Adenocarcinoma	Pancreatic Adenocarcinoma	Pancreas	Primary Tumor	Female	TCGA
297	Tumor	TCGA-FB-A4P5-01	Pancreatic Adenocarcinoma	Pancreatic Adenocarcinoma	Pancreas	Primary Tumor	Female	TCGA
298	Tumor	TCGA-HZ-8317-01	Pancreatic Adenocarcinoma	Pancreatic Adenocarcinoma	Pancreas	Primary Tumor	Female	TCGA
299	Tumor	TCGA-HZ-A49H-01	Pancreatic Adenocarcinoma	Pancreatic Adenocarcinoma	Pancreas	Primary Tumor	Female	TCGA
300	Tumor	TCGA-3A-A9IH-01	Pancreatic Adenocarcinoma	Pancreatic Adenocarcinoma	Pancreas	Primary Tumor	Female	TCGA
301	Tumor	TCGA-IB-7888-01	Pancreatic Adenocarcinoma	Pancreatic Adenocarcinoma	Pancreas	Primary Tumor	Female	TCGA
302	Tumor	TCGA-XD-AAUG-01	Pancreatic Adenocarcinoma	Pancreatic Adenocarcinoma	Pancreas	Primary Tumor	Female	TCGA
303	Tumor	TCGA-FB-AAQ3-01	Pancreatic Adenocarcinoma	Pancreatic Adenocarcinoma	Pancreas	Primary Tumor	Female	TCGA
304	Tumor	TCGA-HV-A5A6-01	Pancreatic Adenocarcinoma	Pancreatic Adenocarcinoma	Pancreas	Primary Tumor	Female	TCGA
305	Tumor	TCGA-HZ-8003-01	Pancreatic Adenocarcinoma	Pancreatic Adenocarcinoma	Pancreas	Primary Tumor	Female	TCGA
306	Tumor	TCGA-IB-7644-01	Pancreatic Adenocarcinoma	Pancreatic Adenocarcinoma	Pancreas	Primary Tumor	Female	TCGA
307	Tumor	TCGA-3A-A9IR-01	Pancreatic Adenocarcinoma	Pancreatic Adenocarcinoma	Pancreas	Primary Tumor	Female	TCGA

308	Tumor	TCGA-IB-7651-01	Pancreatic Adenocarcinoma	Pancreatic Adenocarcinoma	Pancreas	Primary Tumor	Female	TCGA
309	Tumor	TCGA-IB-A5SS-01	Pancreatic Adenocarcinoma	Pancreatic Adenocarcinoma	Pancreas	Primary Tumor	Female	TCGA
310	Tumor	TCGA-IB-A5ST-01	Pancreatic Adenocarcinoma	Pancreatic Adenocarcinoma	Pancreas	Primary Tumor	Female	TCGA
311	Tumor	TCGA-IB-AAUO-01	Pancreatic Adenocarcinoma	Pancreatic Adenocarcinoma	Pancreas	Primary Tumor	Female	TCGA
312	Tumor	TCGA-LB-A8F3-01	Pancreatic Adenocarcinoma	Pancreatic Adenocarcinoma	Pancreas	Primary Tumor	Female	TCGA
313	Tumor	TCGA-Q3-AA2A-01	Pancreatic Adenocarcinoma	Pancreatic Adenocarcinoma	Pancreas	Primary Tumor	Female	TCGA
314	Tumor	TCGA-IB-AAUW-01	Pancreatic Adenocarcinoma	Pancreatic Adenocarcinoma	Pancreas	Primary Tumor	Female	TCGA
315	Tumor	TCGA-LB-A9Q5-01	Pancreatic Adenocarcinoma	Pancreatic Adenocarcinoma	Pancreas	Primary Tumor	Female	TCGA
316	Tumor	TCGA-3A-A9IN-01	Pancreatic Adenocarcinoma	Pancreatic Adenocarcinoma	Pancreas	Primary Tumor	Female	TCGA
317	Tumor	TCGA-FB-AAPS-01	Pancreatic Adenocarcinoma	Pancreatic Adenocarcinoma	Pancreas	Primary Tumor	Female	TCGA
318	Tumor	TCGA-IB-7887-01	Pancreatic Adenocarcinoma	Pancreatic Adenocarcinoma	Pancreas	Primary Tumor	Female	TCGA
319	Tumor	TCGA-3A-A9IC-01	Pancreatic Adenocarcinoma	Pancreatic Adenocarcinoma	Pancreas	Primary Tumor	Female	TCGA
320	Tumor	TCGA-HV-A5A5-01	Pancreatic Adenocarcinoma	Pancreatic Adenocarcinoma	Pancreas	Primary Tumor	Female	TCGA
321	Tumor	TCGA-HZ-7922-01	Pancreatic Adenocarcinoma	Pancreatic Adenocarcinoma	Pancreas	Primary Tumor	Female	TCGA
322	Tumor	TCGA-YY-A8LH-01	Pancreatic Adenocarcinoma	Pancreatic Adenocarcinoma	Pancreas	Primary Tumor	Female	TCGA
323	Tumor	TCGA-2J-AABR-01	Pancreatic Adenocarcinoma	Pancreatic Adenocarcinoma	Pancreas	Primary Tumor	Female	TCGA
324	Tumor	TCGA-HZ-7924-01	Pancreatic Adenocarcinoma	Pancreatic Adenocarcinoma	Pancreas	Primary Tumor	Female	TCGA
325	Tumor	TCGA-RL-AAAS-01	Pancreatic Adenocarcinoma	Pancreatic Adenocarcinoma	Pancreas	Primary Tumor	Female	TCGA
326	Tumor	TCGA-3A-A9IV-01	Pancreatic Adenocarcinoma	Pancreatic Adenocarcinoma	Pancreas	Primary Tumor	Female	TCGA
327	Tumor	TCGA-2J-AABP-01	Pancreatic Adenocarcinoma	Pancreatic Adenocarcinoma	Pancreas	Primary Tumor	Female	TCGA
328	Tumor	TCGA-HZ-8636-01	Pancreatic Adenocarcinoma	Pancreatic Adenocarcinoma	Pancreas	Primary Tumor	Female	TCGA
329	Tumor	TCGA-HZ-A49G-01	Pancreatic Adenocarcinoma	Pancreatic Adenocarcinoma	Pancreas	Primary Tumor	Female	TCGA

330	Tumor	TCGA-XD-AAUH-01	Pancreatic Adenocarcinoma	Pancreatic Adenocarcinoma	Pancreas	Primary Tumor	Female	TCGA
331	Tumor	TCGA-H6-8124-01	Pancreatic Adenocarcinoma	Pancreatic Adenocarcinoma	Pancreas	Primary Tumor	Female	TCGA
332	Tumor	TCGA-IB-A5SQ-01	Pancreatic Adenocarcinoma	Pancreatic Adenocarcinoma	Pancreas	Primary Tumor	Female	TCGA
333	Tumor	TCGA-2J-AABI-01	Pancreatic Adenocarcinoma	Pancreatic Adenocarcinoma	Pancreas	Primary Tumor	Female	TCGA
334	Tumor	TCGA-HZ-A77Q-01	Pancreatic Adenocarcinoma	Pancreatic Adenocarcinoma	Pancreas	Primary Tumor	Female	TCGA
335	Tumor	TCGA-HZ-8315-01	Pancreatic Adenocarcinoma	Pancreatic Adenocarcinoma	Pancreas	Primary Tumor	Female	TCGA
336	Tumor	TCGA-US-A779-01	Pancreatic Adenocarcinoma	Pancreatic Adenocarcinoma	Pancreas	Primary Tumor	Female	TCGA
337	Tumor	TCGA-IB-7897-01	Pancreatic Adenocarcinoma	Pancreatic Adenocarcinoma	Pancreas	Primary Tumor	Female	TCGA
338	Tumor	TCGA-XN-A8T5-01	Pancreatic Adenocarcinoma	Pancreatic Adenocarcinoma	Pancreas	Primary Tumor	Female	TCGA
339	Tumor	TCGA-HZ-7919-01	Pancreatic Adenocarcinoma	Pancreatic Adenocarcinoma	Pancreas	Primary Tumor	Female	TCGA
340	Tumor	TCGA-XD-AAUI-01	Pancreatic Adenocarcinoma	Pancreatic Adenocarcinoma	Pancreas	Primary Tumor	Female	TCGA
341	Tumor	TCGA-2L-AAQJ-01	Pancreatic Adenocarcinoma	Pancreatic Adenocarcinoma	Pancreas	Primary Tumor	Female	TCGA
342	Tumor	TCGA-IB-7652-01	Pancreatic Adenocarcinoma	Pancreatic Adenocarcinoma	Pancreas	Primary Tumor	Female	TCGA
343	Tumor	TCGA-IB-7891-01	Pancreatic Adenocarcinoma	Pancreatic Adenocarcinoma	Pancreas	Primary Tumor	Female	TCGA
344	Tumor	TCGA-L1-A7W4-01	Pancreatic Adenocarcinoma	Pancreatic Adenocarcinoma	Pancreas	Primary Tumor	Female	TCGA
345	Tumor	TCGA-M8-A5N4-01	Pancreatic Adenocarcinoma	Pancreatic Adenocarcinoma	Pancreas	Primary Tumor	Female	TCGA
346	Tumor	TCGA-IB-7645-01	Pancreatic Adenocarcinoma	Pancreatic Adenocarcinoma	Pancreas	Primary Tumor	Female	TCGA
347	Tumor	TCGA-PZ-A5RE-01	Pancreatic Adenocarcinoma	Pancreatic Adenocarcinoma	Pancreas	Primary Tumor	Female	TCGA
348	Tumor	TCGA-FB-AAPU-01	Pancreatic Adenocarcinoma	Pancreatic Adenocarcinoma	Pancreas	Primary Tumor	Female	TCGA
349	Tumor	TCGA-3A-A9IL-01	Pancreatic Adenocarcinoma	Pancreatic Adenocarcinoma	Pancreas	Primary Tumor	Female	TCGA
350	Tumor	TCGA-HZ-A9TJ-06	Pancreatic Adenocarcinoma	Pancreatic Adenocarcinoma	Pancreas	Metastatic	Male	TCGA
