# Supplementary material for: The Solute Carrier Superfamily as Therapeutic Targets in Pancreatic Ductal Adenocarcinoma
Source: Genes (Basel). 2025 Apr 18;16(4):463. doi: 10.3390/genes16040463 (PMC12027052; doi:10.3390/genes16040463)
Supplement: Supplementary file 1 [file genes-16-00463-s001.zip › Table S4.pdf]

Table S3. Overall survival analysis by Cutoff finder

No.	Name	outcome_cutoff	outcome_p	outcome_HR	dis_cutoff	dis_p	dis_HR	mean_cutoff	mean_p	mean_HR
1	SLC1A1	11.490	0.0328556709	1.5605059916	8.797	0.064299288	2.059115798	10.97	0.145827088	1.362531309
2	SLC1A2	5.587	0.0614764559	0.6707922672	8.024	0.7413561	0.921340367	6.668	0.230714712	0.779130097
3	SLC1A3	9.559	0.0046936191	1.8912878699	8.025	0.070022093	1.88356823	9.75	0.019830424	1.649143976
4	SLC1A4	11.440	0.0076117989	0.3630618946	9.792	0.416652636	0.623007694	10.88	0.377028315	0.831112368
5	SLC1A5	12.750	0.0122189975	1.8145578931	11.25	0.001360198	86084553.52	12.91	0.634582314	1.104498139
6	SLC1A6	2.143	0.0707099921	1.5897177218	#N/A	#N/A	#N/A	1.015	0.384109488	1.199437727
7	SLC1A7	5.014	0.0120428828	3.9329854088	6.06	0.352831626	1.310075876	6.875	0.955640881	0.98849187
8	SLC2A1	13.080	0.0000788755	2.6337766792	13.64	0.005845222	1.806764895	13.73	0.000757518	2.071992373
9	SLC2A2	1.111	0.1232421980	2.8667857579	4.245	0.563583934	1.172391764	6.413	0.766768043	1.065207226
10	SLC2A3	11.120	0.0047181690	2.5119743505	#N/A	#N/A	#N/A	12.1	0.032954727	1.560524698
11	SLC2A3P1	6.072	0.4345074614	0.6718778980	#N/A	#N/A	#N/A	0.8263	0.646791866	1.134926076
12	SLC2A3P2	1.578	0.2248870824	1.2904633015	#N/A	#N/A	#N/A	1.542	0.484485719	1.158616563
13	SLC2A3P4	2.009	0.1855816387	1.3232838715	#N/A	#N/A	#N/A	1.926	0.225634262	1.292460404
14	SLC2A4	5.103	0.3899738109	1.2744138189	#N/A	#N/A	#N/A	6.084	0.647260535	0.908392654
15	SLC2A5	6.604	0.0021943310	11.5007055498	6.213	0.006944526	9.371903448	8.658	0.465884244	1.167234437
16	SLC2A6	8.434	0.1749580398	0.7009111183	9.13	0.742283717	0.930684402	9.497	0.916833464	0.978466126
17	SLC2A7	2.118	0.1517257471	0.4404259413	#N/A	#N/A	#N/A	0.4785	0.438640394	0.834292646
18	SLC2A8	9.196	0.0002357495	0.3988299171	9.92	0.103602114	0.712805937	9.916	0.043893639	0.657508642
19	SLC2A9	8.835	0.1871508707	0.4017721096	6.833	0.001930785	3.86664857	7.847	0.691288978	1.086985667
20	SLC2A10	9.545	0.0005881557	8.0781861566	9.866	0.005650501	3.366639733	11.18	0.018615071	1.664689288
21	SLC2A11	8.954	0.0219456355	0.5580073603	#N/A	#N/A	#N/A	9.532	0.279596567	0.798283078
22	SLC2A12	6.302	0.0083155574	5.4389587011	5.935	0.071221439	5.102569725	8.143	0.483380459	1.158752598
23	SLC2A13	9.306	0.2874917424	1.4514571716	8.672	0.876178409	1.074423185	10.15	0.521975064	1.142557736
24	SLC2A14	1.190	0.0044103111	4.9219026034	3.93	0.247856126	1.278267992	4.205	0.318629022	1.23277579
25	SLC3A1	6.054	0.0083298672	4.1970291240	9.367	0.17440026	1.440326791	11	0.145842849	1.367483357
26	SLC3A2	14.150	0.5791787309	1.1951085161	14.48	0.440334388	0.578661023	13.38	0.512158932	1.146133867
27	SLC4A1	0.779	0.0036995283	0.5472669912	#N/A	#N/A	#N/A	1.18	0.048071869	0.657907435
28	SLC4A2	12.780	0.0375365286	1.6585121093	12.88	0.045104794	1.585103032	13.09	0.319995152	1.234519649

29	SLC4A3	9.411	0.0151102057	0.5902993933	#N/A	#N/A	#N/A	9.208	0.067956788	0.684985491
30	SLC4A4	15.380	0.0633633484	0.1887024186	11.31	0.108607251	1.507969685	12.41	0.576018312	1.125128199
31	SLC4A5	7.671	0.0445046594	0.5707297312	6.231	0.879249919	1.040894203	7.081	0.422486529	0.844254437
32	SLC4A7	9.896	0.0006091473	3.0310760051	9.087	0.037249264	2.789173273	10.5	0.608314444	1.113861126
33	SLC4A8	7.332	0.0010927856	0.4798937547	10.26	0.005158449	0.259975512	8.356	0.001132699	0.503023682
34	SLC4A9	4.834	0.1357180545	0.3598001898	2.356	0.604494438	1.115463355	2.647	0.633459584	1.104195542
35	SLC4A10	6.052	0.0946509847	0.7068880781	6.295	0.11275181	0.718032568	6.116	0.119456165	0.723686788
36	SLC4A11	10.520	0.1304415223	1.3754917912	11.25	0.563397514	1.154470561	9.78	0.246491552	1.273998473
37	SLC5A1	12.590	0.0584586445	1.5486523441	8.688	0.011063386	3.99419327	11.27	0.505939673	1.15396611
38	SLC5A2	6.815	0.0967035202	1.4202071473	3.896	0.783410317	0.890097887	6.163	0.613535087	0.900341639
39	SLC5A3	10.460	0.0011767500	2.0420222036	9.469	0.00735446	3.255558292	10.55	0.011625471	1.703078265
40	SLC5A4	3.840	0.0517533717	0.6681249586	2.897	0.957377625	0.984584465	4.098	0.436415124	0.850877929
41	SLC5A5	0.949	0.0660295729	2.4921223290	7.666	0.893749667	1.070793838	3.757	0.606304936	1.112928067
42	SLC5A6	10.310	0.0053335360	3.0981148387	10.11	0.012188496	3.034752715	10.83	0.96319355	0.990392482
43	SLC5A7	0.464	0.0676187343	1.7494594118	#N/A	#N/A	#N/A	3.432	0.896601871	0.973323908
44	SLC5A8	4.326	0.1872501275	0.5981142167	3.648	0.6057564	0.847081822	1.914	0.414273837	1.185594291
45	SLC5A9	8.550	0.1619255185	0.6282615002	4.864	0.437535811	0.786372839	6.9	0.618240827	1.110320686
46	SLC5A10	5.145	0.1289452351	0.6827729386	3.171	0.400734568	0.770934017	4.598	0.985371043	1.003820554
47	SLC5A11	1.471	0.0175089921	0.5744218186	2.681	0.554337896	0.883689856	2.637	0.771574164	0.94135364
48	SLC5A12	1.348	0.0372664651	0.5984496353	2.662	0.087509198	0.701541732	3.225	0.077975119	0.689694992
49	SLC6A1	8.600	0.1914626006	0.4724985334	#N/A	#N/A	#N/A	7.068	0.129503476	1.376444477
50	SLC6A2	3.464	0.2309894146	1.3604395419	3.74	0.677648831	1.132776238	1.957	0.95632976	1.0115173
51	SLC6A3	1.055	0.0496096927	1.8664095276	#N/A	#N/A	#N/A	2.693	0.260168598	1.266248953
52	SLC6A4	7.835	0.0077390950	0.3391912327	10.66	0.099718161	3.91288E-08	5.913	0.101847812	0.712402332
53	SLC6A5	0.885	0.8136534933	0.9239569677	#N/A	#N/A	#N/A	0.1634	0.442979264	0.773321141
54	SLC6A6	11.850	0.0002785091	6.5334797936	#N/A	#N/A	#N/A	12.91	0.388688288	1.201821894
55	SLC6A7	3.170	0.6939845035	0.9124432997	2.6	0.883215942	1.043928957	4.006	0.334678335	1.222008217
56	SLC6A8	11.340	0.0319690709	1.8161188248	14.6	0.70138556	1.193112442	12.3	0.270555257	1.260611605
57	SLC6A9	7.445	0.0019760983	11.6977064665	8.043	0.077908934	1.846425438	8.915	0.658162128	1.097718738
58	SLC6A10P	2.976	0.5011164624	0.8176212242	#N/A	#N/A	#N/A	1.638	0.837674013	1.044150757

59	SLC6A11	3.463	0.0770748540	1.4406332819	4.07	0.478247755	1.183740328	3.114	0.036771582	1.546299824
60	SLC6A12	6.041	0.0297394712	1.7802319086	4.453	0.145325313	2.082038264	6.855	0.267042306	1.261511634
61	SLC6A13	2.022	0.0123995611	0.5862372617	2.24	0.024506732	0.625736432	2.688	0.021326162	0.619360737
62	SLC6A14	6.791	0.0001125374	17.4311616528	6.765	0.000112537	17.43116165	10.68	0.000673711	2.148992331
63	SLC6A15	3.806	0.5997608319	0.8932028220	#N/A	#N/A	#N/A	3.321	0.809968568	1.051455417
64	SLC6A16	6.950	0.0013396960	0.3189555075	4.816	0.924135593	0.974092251	5.863	0.48422627	0.864583407
65	SLC6A17	10.160	0.0016227801	0.3076354999	9.214	0.169644187	0.737835362	8.161	0.290895982	0.803297857
66	SLC6A18	1.881	0.1730241526	0.5659000461	#N/A	#N/A	#N/A	0.492	0.768016264	0.931801819
67	SLC6A19	8.726	0.0303235228	0.4558063361	7	0.266932967	0.791426309	6.197	0.526541445	0.876322186
68	SLC6A20	9.980	0.0009891404	2.7775169039	8.324	0.00043947	6.262879299	10.69	0.032538871	1.644777231
69	SLC7A1	11.840	0.0034574679	2.1620447752	12.21	0.792664925	1.056353326	12.17	0.523124728	1.14335395
70	SLC7A2	13.090	0.0392801121	0.3145469810	#N/A	#N/A	#N/A	11.09	0.667747472	0.91471209
71	SLC7A3	5.551	0.1182135861	1.5508525959	3.267	0.400018418	0.833850424	3.839	0.210733798	0.771124538
72	SLC7A4	7.166	0.9530647826	1.0145178749	7.967	0.350275515	0.819355728	7.966	0.350275515	0.819355728
73	SLC7A5	13.370	0.1046856787	1.6191433797	14.08	0.327382526	1.643232035	11.93	0.113772705	1.392476662
74	SLC7A5P1	3.919	0.1489258684	0.4849528903	#N/A	#N/A	#N/A	2.529	0.247287407	1.275982798
75	SLC7A6	9.564	0.0710266137	1.9319411899	10.23	0.691743156	1.0930758	10.42	0.791933016	1.05649708
76	SLC7A7	10.780	0.0003577760	3.5013828717	9.873	0.000597025	8.099838395	11.52	0.067264584	1.489178167
77	SLC7A8	11.570	0.0123592158	0.5968017435	12.97	0.044259372	0.404602363	11.8	0.033778826	0.639183334
78	SLC7A9	4.815	0.4603400914	1.1749787317	8.695	0.117096711	0.23500499	5.21	0.382042903	1.199941294
79	SLC7A10	5.887	0.0301034790	0.2399617593	2.769	0.03269433	0.590907764	2.129	0.045779529	0.641222733
80	SLC7A11	7.354	0.0002708971	88081155.710	#N/A	#N/A	#N/A	9.739	0.103780075	1.404691293
81	SLC7A13	0.885	0.0049500295	1.9889015337	#N/A	#N/A	#N/A	0.3747	0.061199166	1.568642099
82	SLC7A14	8.642	0.0016254678	0.2580434115	5.361	0.028403303	0.626239084	6.17	0.058519245	0.674021975
83	SLC7A15P	#N/A	#N/A	#N/A	#N/A	#N/A	#N/A	#N/A	#N/A	#N/A
84	SLC8A1	9.962	0.0822931098	0.6851321745	9.282	0.022008378	0.611295751	9.642	0.103748195	0.714405168
85	SLC8A2	9.284	0.0162846865	0.3102107637	7.258	0.50564349	0.870042248	6.989	0.77200854	0.940858679
86	SLC8A3	7.124	0.0025599137	0.1516206746	4.531	0.049883615	0.652639809	5.197	0.177357931	0.754594968
87	SLC8B1	10.780	0.0018625222	5.2507821144	#N/A	#N/A	#N/A	11.65	0.084360163	1.446764716
88	SLC9A1	11.230	0.0001438439	93015550.133	11.2	0.000165084	93213986.17	12.64	0.450693949	1.170566854

89	SLC9A2	6.003	0.0022083759	3.1739217236	4.829	0.028344633	2.657242023	8.174	0.312395809	1.242841778
90	SLC9A3	9.444	0.2422394117	0.7522247960	7.468	0.697138101	1.084258611	7.472	0.697138101	1.084258611
91	SLC9A3P1	#N/A	#N/A	#N/A	#N/A	#N/A	#N/A	0.03815	0.583268166	0.676665496
92	SLC9A3P2	0.321	0.7527637524	0.8512285084	#N/A	#N/A	#N/A	0.1294	0.752763752	0.851228508
93	SLC9A3P3	0.804	0.3140655386	0.7399493623	#N/A	#N/A	#N/A	0.2549	0.509084582	0.829795985
94	SLC9A4	1.516	0.0003523222	4.5595445456	6.134	0.171242449	1.328805567	5.834	0.051421067	1.503514672
95	SLC9A5	5.411	0.4807109847	0.8330997312	5.916	0.733738545	0.927164595	6.345	0.335575382	0.818787735
96	SLC9A6	10.440	0.1555014292	1.4071352326	10.21	0.131479083	1.596341391	10.56	0.23624096	1.290062755
97	SLC9A7	10.530	0.2000020742	1.4009941025	12.7	0.008859177	3.593163922	11.01	0.795990323	0.947548367
98	SLC9A7P1	5.258	0.0204391530	0.5604371950	4.277	0.700826747	1.217013277	6.098	0.650013801	0.90950836
99	SLC9A8	11.020	0.1018431394	1.4335603793	11.17	0.548238723	1.133265276	11.16	0.436995494	1.176015468
100	SLC9A9	9.552	0.1462956870	0.6648005056	7.378	0.307846196	1.458983178	8.785	0.894705641	0.972677074
101	SLC9B1	4.584	0.0004335789	0.4125491199	3.217	0.264085328	0.707625387	4.217	0.004288419	0.552431721
102	SLC9B2	11.090	0.0089399715	1.7137002302	#N/A	#N/A	#N/A	10.7	0.091644332	1.42892139
103	SLC9C1	4.618	0.0150426296	0.2578367921	2.474	0.754320651	0.929030012	2.976	0.731524191	0.930008669
104	SLC9C2	2.430	0.0625739835	1.4870693021	#N/A	#N/A	#N/A	2.497	0.226313723	1.290063152
105	SLC10A1	1.334	0.0778544381	0.6805533781	#N/A	#N/A	#N/A	2.023	0.218372066	0.77396837
106	SLC10A2	1.368	0.0002514271	2.1528504896	#N/A	#N/A	#N/A	1.547	0.001506917	1.942685619
107	SLC10A3	11.620	0.0008171001	2.0174278382	10.6	0.004335179	4.641299403	11.56	0.010804956	1.724576881
108	SLC10A4	5.073	0.0326980561	0.3498033646	2.233	0.439629497	0.82482978	3.365	0.305884631	0.806490885
109	SLC10A5	6.339	0.2543945044	1.3332687174	6.148	0.647464604	1.113475561	5.7	0.808229499	0.950759446
110	SLC10A6	5.007	0.0994140262	0.6346381455	2.218	0.76768474	1.145744281	4.307	0.526960456	1.140937711
111	SLC10A7	9.745	0.0007005264	2.0579031954	8.177	0.324880273	1.994942625	9.373	0.027817223	1.592438846
112	SLC11A1	8.766	0.0000666039	7.4673093376	10.04	0.037874193	1.608533831	10.56	0.117278139	1.393541599
113	SLC11A2	11.460	0.0414973553	1.8280797666	12.57	0.329029186	1.640584291	11.74	0.442135963	1.172887598
114	SLC12A1	3.353	0.0004824261	0.3117954418	4.568	0.004216909	0.16345483	2.238	0.004608446	0.554524157
115	SLC12A2	11.150	0.0000178900	20.8863323121	11.28	0.000167114	6.830558661	12.63	0.80181668	1.053988808
116	SLC12A3	5.178	0.3889394785	0.6730835626	2.752	0.70285127	1.09865263	3.562	0.972835595	1.007151997
117	SLC12A4	11.210	0.0000766118	7.3730310863	11.19	7.66118E-05	7.373031086	11.97	0.438914302	1.180447584
118	SLC12A5	7.202	0.0013599130	0.3347219579	8.575	0.00272722	0.089094412	6.233	0.126462207	0.725978712

119	SLC12A6	10.550	0.0007382239	3.8366276163	10.07	0.009446848	4.103560957	11.12	0.500912935	1.154619328
120	SLC12A7	12.650	0.0038634959	2.5519867213	11.66	0.415737981	1.780180208	13.21	0.492259454	1.154092713
121	SLC12A8	10.070	0.0004196111	3.7941807561	9.067	0.001754948	86774968.64	10.69	0.195019368	1.314255412
122	SLC12A9	10.820	0.0874054768	0.5669201538	11.87	0.458895949	1.172487199	11.95	0.685699712	1.088324476
123	SLC13A1	1.548	0.1837634403	0.7445035490	#N/A	#N/A	#N/A	1.152	0.907955987	0.976122306
124	SLC13A2	1.962	0.0809138217	2.0585810092	5.446	0.210389804	1.305383978	5.819	0.157578041	1.346958811
125	SLC13A3	8.839	0.0000252190	3.2543591190	6.588	0.01951946	2.125535395	7.61	0.499740293	1.154993569
126	SLC13A4	5.654	0.5919593427	0.8911668882	5.133	0.294740447	1.383014696	6.103	0.41503004	1.185215461
127	SLC13A5	9.984	0.0016829717	1.9482897430	7.337	0.034445127	1.564677581	7.912	0.033977566	1.557406777
128	SLC14A1	10.280	0.0789202005	1.6616027133	9.361	0.204052868	1.39507641	7.79	0.500351986	0.869257206
129	SLC14A2	3.697	0.0146726043	0.3945172703	#N/A	#N/A	#N/A	2.439	0.300500267	0.806633348
130	SLC15A1	9.154	0.0278224562	1.7192338602	6.741	0.080205917	2.077376682	9.534	0.207165189	1.322936132
131	SLC15A2	6.436	0.0021708193	6.7630120684	8.357	0.539316556	1.145063203	8.865	0.198489828	1.317027462
132	SLC15A3	9.172	0.0014221927	12.3370241729	10.64	0.062158328	1.779023168	11.41	0.642851541	1.104019384
133	SLC15A4	10.340	0.0008433375	5.7345403980	9.938	0.049941939	3.005124658	11.02	0.203694817	1.307645527
134	SLC16A1	11.160	0.0013650350	2.1301702165	#N/A	#N/A	#N/A	11.37	0.118280107	1.394060705
135	SLC16A2	11.110	0.0393685257	0.5919123999	9.464	0.360531933	1.520046474	10.65	0.511094584	0.871072463
136	SLC16A3	10.610	0.0006339924	14.0591134451	11.11	0.002711778	4.985899451	13.21	0.317309673	1.233245309
137	SLC16A4	7.074	0.0034916684	10.6352836299	8.551	0.074934203	1.770554989	10.04	0.212737414	1.309570001
138	SLC16A5	10.870	0.0053199753	1.7773753996	7.612	0.000796675	7.793083091	10.35	0.135335625	1.374962558
139	SLC16A6	6.488	0.1037645404	1.8778085284	5.697	0.273076818	1.883497985	7.652	0.810231823	1.051359236
140	SLC16A7	10.820	0.1347032356	1.3657612295	#N/A	#N/A	#N/A	10.36	0.601776494	1.116572532
141	SLC16A8	3.689	0.0925751283	0.6311211055	7.529	0.950229464	0.939087051	4.919	0.826231829	0.955435905
142	SLC16A9	6.652	0.1039631275	1.7592316550	10.87	0.517145705	0.631545473	8.075	0.695211138	1.08531016
143	SLC16A10	9.109	0.0023117829	1.8895844251	8.336	0.325875351	1.233506137	8.562	0.399671144	1.194032809
144	SLC16A11	7.446	0.0000118885	0.2369456174	5.175	0.206809852	0.723210434	6.389	0.066021898	0.681366564
145	SLC16A12	9.918	0.0175650545	0.4033323779	7.227	0.521731797	0.875008687	7.293	0.504107566	0.870219764
146	SLC16A13	6.801	0.0281511426	6.7318113153	7.3	0.070423442	1.870407961	7.986	0.707354045	0.924949695
147	SLC16A14	7.689	0.0381948162	1.8467798188	7.358	0.090625341	2.022546252	8.365	0.846846653	1.041408393
148	SLC17A1	1.974	0.3669984419	1.2061564681	#N/A	#N/A	#N/A	1.554	0.666209513	1.093800782

149	SLC17A2	2.616	0.0594834223	0.3468689367	#N/A	#N/A	#N/A	0.6197	0.674791158	0.914028142
150	SLC17A3	3.190	0.0717964285	0.3618704335	#N/A	#N/A	#N/A	1.119	0.589404363	1.118697046
151	SLC17A4	10.330	0.2509368067	0.5600107694	5.065	0.551121373	1.17858407	7.29	0.738582757	1.073665128
152	SLC17A5	11.030	0.0073941027	1.9224686167	11.75	0.098404613	1.527149757	11.21	0.146534373	1.356316973
153	SLC17A6	3.823	0.5195990696	1.1432113653	#N/A	#N/A	#N/A	3.496	0.587530357	1.119652694
154	SLC17A7	4.288	0.0145023292	3.8165764792	4.002	0.167927814	2.202736897	6.161	0.933968024	1.017485731
155	SLC17A8	1.996	0.3881100974	1.2015589843	#N/A	#N/A	#N/A	1.469	0.871485818	0.966542166
156	SLC17A9	11.840	0.1044897308	1.4383439484	8.78	0.044788181	2.484862076	10.8	0.721722288	1.077050087
157	SLC18A1	3.866	0.0047140917	0.3218701208	5.941	0.102232257	0.329237077	2.448	0.357813736	0.825604526
158	SLC18A2	8.531	0.1901586007	0.6467678987	6.019	0.823302628	0.941601403	7.257	0.193640748	0.762411539
159	SLC18A3	6.072	0.0062581805	0.2643716046	#N/A	#N/A	#N/A	3.407	0.229793073	0.779374471
160	SLC18B1	10.650	0.3657636178	1.2357457237	9.432	0.154304676	1.815350285	10.25	0.98320187	1.004390699
161	SLC19A1	8.675	0.0003980860	15.0284249358	#N/A	#N/A	#N/A	10.05	0.261441254	1.270638802
162	SLC19A2	9.470	0.0001708495	87870678.645	9.093	0.000541809	86947854.73	10.51	0.274010433	1.257824408
163	SLC19A3	9.443	0.0866561132	0.6775662858	#N/A	#N/A	#N/A	8.645	0.151238736	0.742521709
164	SLC20A1	10.370	0.0001648708	94381862.842	11.19	0.001158186	5.576992736	12.57	0.17921772	1.32439973
165	SLC20A2	11.430	0.0106643611	2.6430402394	10.76	0.011051672	5.166313185	11.94	0.11973965	1.387878174
166	SLCO2A1	11.990	0.0538906303	0.6701040736	11.64	0.23491404	0.774791885	12.03	0.104297831	0.71304239
167	SLCO4A1	7.686	0.0004801477	14.6351914830	10.27	0.226969225	1.28648942	10.32	0.226969225	1.28648942
168	SLCO5A1	6.381	0.0092293817	0.5599634860	7.89	0.189486779	0.710904432	7.104	0.052391843	0.661712692
169	SLCO3A1	10.210	0.0001644179	16.5512945646	9.934	0.000708206	13.64860705	11.21	0.094746136	1.435855426
170	SLCO6A1	1.009	0.0324158405	0.4135180950	#N/A	#N/A	#N/A	0.2509	0.049627101	0.506121566
171	SLCO1A2	2.346	0.6855388293	0.9173640297	#N/A	#N/A	#N/A	3.226	0.936717261	1.016665802
172	SLCO2B1	11.880	0.3574233446	0.8242942184	12.04	0.506716143	0.8702887	12	0.546758143	0.881466284
173	SLCO1B1	2.387	0.0518080107	1.5164935806	#N/A	#N/A	#N/A	1.718	0.49324167	1.155607412
174	SLCO1B3	9.564	0.0009805248	2.3386790692	6.947	0.159124575	1.347995421	5.655	0.0274836	1.585787739
175	SLCO4C1	6.071	0.0128919912	0.5950060719	5.088	0.805717225	0.945621344	5.904	0.023146632	0.621831854
176	SLCO1C1	5.480	0.2176766979	0.7132646146	3.803	0.382179232	0.818801376	4.522	0.431515121	1.177634345
177	SLC22A1	3.194	0.6696110011	0.9035140816	2.24	0.991856234	1.004329721	4.008	0.835066015	0.957631707
178	SLC22A2	1.341	0.6743160976	0.9079201813	5.939	0.417778406	0.451907815	2.323	0.806210551	1.052540354

179	SLC22A3	7.632	0.0009729978	13.2307124577	7.451	0.002506744	11.31564566	9.972	0.239174259	1.286072136
180	SLC22A4	7.926	0.1733044240	0.6665773803	6.033	0.610576167	0.791234428	7.346	0.632058237	0.905143946
181	SLC22A5	10.690	0.0288758208	0.4541946289	#N/A	#N/A	#N/A	10.04	0.982005403	1.004700702
182	SLC22A6	#N/A	#N/A	#N/A	#N/A	#N/A	#N/A	0.1026	0.501927459	0.710035394
183	SLC22A7	1.409	0.0285418718	0.6079401343	#N/A	#N/A	#N/A	1.068	0.224054097	0.771726187
184	SLC22A8	0.321	0.1032782974	0.4795500411	#N/A	#N/A	#N/A	0.07323	0.103278297	0.479550041
185	SLC22A9	2.112	0.0266880449	0.6272357234	2.733	0.147580127	0.735071368	2.71	0.101377024	0.70661009
186	SLC22A10	1.346	0.0070196220	0.5040953437	#N/A	#N/A	#N/A	0.8741	0.106187391	0.709753247
187	SLC22A11	1.804	0.3002776785	0.6802117626	2.669	0.425170859	1.324442793	4.763	0.193027161	0.762259912
188	SLC22A12	1.216	0.0459856654	0.4403373817	#N/A	#N/A	#N/A	0.3085	0.21127221	0.688944951
189	SLC22A13	4.004	0.8252703786	0.8782738562	#N/A	#N/A	#N/A	2.098	0.818544237	1.049625579
190	SLC22A14	2.067	0.2313269339	0.7598603595	2.688	0.975315723	0.993479384	2.891	0.833417419	1.044796067
191	SLC22A15	8.009	0.1324100790	1.7343378439	#N/A	#N/A	#N/A	8.847	0.358387564	1.210145298
192	SLC22A16	4.241	0.0228508413	0.5470851068	2.124	0.699355327	0.896592405	3.479	0.106127443	0.714724103
193	SLC22A17	11.440	0.0019212564	0.4049805221	12.72	0.002422747	0.148787697	10.95	0.00522154	0.555650565
194	SLC22A18	8.767	0.0028156518	6.5416764570	10.25	0.045348341	1.681369311	11.17	0.24273979	1.278628998
195	SLC22A20	6.854	0.0988502712	1.4495315515	4.302	0.082461545	1.649517225	5.761	0.489860857	1.154795369
196	SLC22A23	11.010	0.4578355938	0.8567207227	10.93	0.72524827	0.929237527	11.01	0.522681468	0.875374207
197	SLC22A24	#N/A	#N/A	#N/A	#N/A	#N/A	#N/A	0.02371	0.246292015	0.442648905
198	SLC22A25	1.411	0.1711506776	1.4883072917	#N/A	#N/A	#N/A	0.3611	0.612865312	0.881814265
199	SLC22A31	2.148	0.0365938378	2.1483423575	7.082	0.99705622	1.00124224	4.384	0.204655185	1.30625215
200	SLC22A32	13.430	0.0266407776	1.6385080354	12.84	0.110460405	1.392028424	12.64	0.040398327	1.54098543
201	SLC22B1	10.330	0.0011760419	0.2965136013	7.816	0.495355641	0.731348514	9.415	0.104908948	0.706789657
202	SLC22B2	7.385	0.3870266862	0.8236597694	6.044	0.983464062	1.007316639	8.395	0.738270213	0.932515745
203	SLC22B3	3.304	0.0219791082	3.0559981964	3.77	0.077424238	1.848302313	5.336	0.416754517	1.185225178
204	SLC22B4	8.910	0.0030917260	0.3447061320	6.645	0.02550634	0.630506479	6.655	0.02550634	0.630506479
205	SLC22B5	4.790	0.0041760038	1.8355363088	3.628	0.074185516	1.453893701	3.648	0.046700172	1.516397771
206	SLC23A1	5.486	0.0107536526	1.8260464293	4.654	0.039027609	1.888988231	5.817	0.065939773	1.481078941
207	SLC23A2	11.110	0.0002206819	0.4660259011	11.01	0.038122094	0.627083182	11.27	0.059960809	0.676334638
208	SLC23A3	4.104	0.0276859262	3.4076611441	6.229	0.421100337	1.196901654	6.932	0.852572086	1.039648316

209	SLC24A1	10.170	0.0230787177	1.8570292107	8.956	0.069459284	2.038872747	9.644	0.54578304	1.135500053
210	SLC24A2	4.336	0.0359996220	2.3695304818	3.253	0.061700323	2.87167749	6.586	0.042504708	1.534404831
211	SLC24A3	11.580	0.1199556692	0.4129118326	9.929	0.278021498	1.288299608	10.24	0.725402817	0.928959143
212	SLC24A4	5.739	0.0241266647	0.6144734000	2.334	0.922419327	0.932596569	5.51	0.187508696	0.758930223
213	SLC24A5	0.865	0.0036948876	0.4441759663	#N/A	#N/A	#N/A	0.4699	0.00860743	0.495345503
214	SLC25A1	12.660	0.0426142060	1.5956690473	12.52	0.047717228	1.537287509	12.05	0.528009178	1.139998328
215	SLC25A2	0.939	0.5147908274	0.8586038916	#N/A	#N/A	#N/A	1.921	0.775564794	1.061006963
216	SLC25A3	14.780	0.0008557851	2.5066186197	14.46	0.375650478	1.220138867	14.23	0.321861045	1.229849157
217	SLC25A4	11.340	0.0023141096	0.3852590880	11.46	0.048916236	0.531829307	10.92	0.116696809	0.714440274
218	SLC25A5	13.190	0.0128846495	8.1883139238	12.95	0.026253698	28646304.53	14.01	0.452054093	1.169082828
219	SLC25A5P1	3.371	0.0654008296	0.6738782810	2.158	0.713974922	0.87898362	3.843	0.86445993	0.964872558
220	SLC25A6	14.890	0.2110854430	0.7691966112	14.72	0.704997517	1.08387768	14.87	0.591420311	0.894358739
221	SLC25A6P1	#N/A	#N/A	#N/A	#N/A	#N/A	#N/A	#N/A	#N/A	#N/A
222	SLC25A7	2.538	0.0516825027	0.3813595451	#N/A	#N/A	#N/A	0.9742	0.611534287	0.899560501
223	SLC25A8	13.610	0.3940118720	0.6483287446	12.27	0.228038426	0.770165418	12.14	0.0676606	0.683625755
224	SLC25A9	5.993	0.0687115950	0.6687600287	6.24	0.13196084	0.700009137	5.727	0.095185487	0.706637495
225	SLC25A10	11.810	0.0106932950	1.9100604249	11.04	0.251673979	1.268720031	10.78	0.163410996	1.341869481
226	SLC25A11	11.120	0.0233860142	0.5600741692	12.23	0.103571801	3.91474E-08	11.5	0.373979794	0.831159312
227	SLC25A12	10.750	0.1561324322	1.3559225025	9.487	0.513341473	1.349729936	10.44	0.534746553	1.138989806
228	SLC25A13	10.320	0.0011197836	12.7927375103	11.11	0.264931325	1.26631204	10.95	0.041529102	1.531348953
229	SLC25A14	9.584	0.0047005517	0.2574084317	10.03	0.003564264	1.18152E-08	9.092	0.073427623	0.68619562
230	SLC25A15	7.867	0.0252729174	1.7348935745	6.939	0.002670774	6.559668639	8.052	0.052491417	1.53468212
231	SLC25A15P1	#N/A	#N/A	#N/A	#N/A	#N/A	#N/A	#N/A	#N/A	#N/A
232	SLC25A16	10.490	0.0090058173	1.7939418458	10.62	0.157970733	1.459245399	10.15	0.411235563	0.841472623
233	SLC25A17	10.880	0.0002383971	2.5658005620	10.21	0.028160308	1.65018803	10.4	0.223516651	1.286949052
234	SLC25A18	6.845	0.0731356615	0.4771061233	4.279	0.209982021	0.591554148	5.749	0.854071184	0.962142979
235	SLC25A19	8.583	0.0090574788	5.3511904267	9.9	0.670330555	0.889577156	9.466	0.467633786	1.163243931
236	SLC25A20	9.986	0.0655902294	1.6624337173	10.29	0.710643551	1.080663878	10.3	0.575861567	1.123994281
237	SLC25A20P1	#N/A	#N/A	#N/A	#N/A	#N/A	#N/A	0.009083	0.703691107	8.233E-07
238	SLC25A21	4.240	0.0022754220	4.2085469281	4.347	0.018890294	2.236373593	5.5	0.429174102	1.179408071

239	SLC25A22	11.220	0.0122259557	1.6753920109	11.08	0.150693683	1.348358426	11.07	0.150693683	1.348358426
240	SLC25A23	11.930	0.1331923125	1.4207679704	12.08	0.433710361	1.185226031	12.21	0.423760579	1.181332495
241	SLC25A24	10.900	0.0000332127	6.4706132799	10.59	0.000217878	6.729149296	11.64	0.066792657	1.47976274
242	SLC25A25	13.140	0.1606709378	1.6299718938	11.8	0.919129811	0.978457677	11.77	0.87825495	0.967862261
243	SLC25A26	10.780	0.0204966356	1.7014770135	9.49	0.082394542	3.24634226	10.34	0.159732222	1.339758066
244	SLC25A27	9.255	0.0000307875	0.1509172340	6.737	0.33649362	0.756571803	8.095	0.015441024	0.602213173
245	SLC25A28	10.590	0.5305646371	0.8719393811	11.46	0.332608859	1.289954934	10.93	0.794856828	0.947360406
246	SLC25A29	10.180	0.1268087235	0.6349018507	11.5	0.109611363	0.71716311	11.56	0.081641696	0.695406794
247	SLC25A30	9.770	0.0014945643	2.2067413200	9.352	0.003386554	3.014476978	9.989	0.03543805	1.582886114
248	SLC25A31	1.786	0.2150814119	1.4869764592	#N/A	#N/A	#N/A	0.5209	0.999533412	1.000130677
249	SLC25A32	11.010	0.0685133235	1.5681107739	#N/A	#N/A	#N/A	10.59	0.105378623	1.404172841
250	SLC25A33	9.453	0.5079559561	1.1534886883	10.44	0.420766903	0.453883491	9.227	0.957721749	0.989043953
251	SLC25A34	6.980	0.0103896658	0.5906491065	7.984	0.019769211	0.546663241	7.25	0.249207347	0.786734344
252	SLC25A35	8.135	0.1019874725	0.6878448431	6.969	1	NA	8.639	0.635554854	0.906226975
253	SLC25A36	11.000	0.0593704853	2.8818275453	11.04	0.127792277	2.141537068	11.78	0.125779397	1.377108226
254	SLC25A37	10.210	0.0003060210	15.5255063301	12.43	0.262479376	1.273806616	12.05	0.410557278	1.187422459
255	SLC25A38	11.560	0.0056991284	2.1175329696	11.14	0.56095133	1.129503929	11.11	0.755408297	1.066877612
256	SLC25A39	13.700	0.0010126024	2.0533628672	13.16	0.054984199	1.488688424	13.15	0.054984199	1.488688424
257	SLC25A40	9.627	0.0010660458	4.0604669634	9.596	0.002771476	3.657283302	10.11	0.133613752	1.378021589
258	SLC25A41	2.438	0.0123746544	0.5662467953	3.27	0.181361114	0.756538876	3.492	0.143792327	0.737089256
259	SLC25A42	9.547	0.0048542208	0.4685426050	9.857	0.044704043	0.461587386	9.318	0.124429172	0.726684588
260	SLC25A43	10.070	0.0001747597	2.7858311337	#N/A	#N/A	#N/A	10.36	0.0014302	1.988418743
261	SLC25A44	10.950	0.1704103807	1.4697756106	10.39	0.173345722	3.598396973	11.2	0.197871564	0.764899757
262	SLC25A45	8.788	0.0112332578	0.5938454190	10.06	0.990239438	1.005674496	9.028	0.049443844	0.658167494
263	SLC25A46	11.980	0.0000024753	3.0334414872	#N/A	#N/A	#N/A	11.57	0.19839897	1.307654711
264	SLC25A47	4.379	0.0021590346	0.1494937755	#N/A	#N/A	#N/A	2.17	0.357810871	0.826058354
265	SLC25A48	3.936	0.0093354533	1.7129879651	3.25	0.041441113	1.558434481	3.485	0.116835107	1.397158884
266	SLC25A49	13.890	0.3584813282	1.2291515762	12.67	0.492177616	1.627417822	13.61	0.431186548	1.177516502
267	SLC25A50	11.610	0.0007551959	3.7650033821	#N/A	#N/A	#N/A	12	0.13246383	1.3671143
268	SLC25A51	8.660	0.1175287889	1.6173856915	8.347	0.980434218	1.011351676	9.016	0.3171422	0.811166545

269	SLC25A51P1	#N/A	#N/A	#N/A	#N/A	#N/A	#N/A	0.0251	0.880771303	0.859797303
270	SLC25A51P2	#N/A	#N/A	#N/A	#N/A	#N/A	#N/A	#N/A	#N/A	#N/A
271	SLC25A51P3	#N/A	#N/A	#N/A	#N/A	#N/A	#N/A	0.004195	0.683247632	1.504331211
272	SLC25A52	1.072	0.0316569351	0.6298842088	#N/A	#N/A	#N/A	0.8914	0.021978204	0.6152479
273	SLC25A53	7.212	0.0049755553	0.5619063749	8.437	0.046423366	0.371726848	7.453	0.149925752	0.735973539
274	SLC26A1	5.841	0.0576922627	0.6347555034	6.464	0.310012413	0.804666805	6.871	0.298202064	0.806044734
275	SLC26A2	11.370	0.0450470541	1.7836062684	9.448	0.908013144	1.06091775	10.71	0.750799571	0.935886107
276	SLC26A3	7.556	0.0225355356	1.8350779137	#N/A	#N/A	#N/A	3.101	0.965077213	0.99087997
277	SLC26A4	3.883	0.0834096597	2.0497590430	6.409	0.668897003	1.14268994	5.254	0.849609232	1.040182969
278	SLC26A5	3.478	0.1990569980	1.3052635495	3.086	0.343301485	1.217625367	3.12	0.230896766	1.282415958
279	SLC26A6	11.000	0.4965426372	1.1667073366	10.39	0.294439499	1.243185865	10.36	0.277335734	1.253734047
280	SLC26A7	6.779	0.0041102985	0.2853274691	4.843	0.759037529	1.069878119	5.285	0.953053525	0.987814724
281	SLC26A8	3.840	0.1503710597	1.3693064368	1.709	0.664120066	1.188991974	3.156	0.773332894	1.061769873
282	SLC26A9	4.377	0.0010132522	13.2197276679	6.253	0.035760631	2.261631773	10.15	0.720285692	0.92824497
283	SLC26A10	6.367	0.0074414410	0.5741996481	5.831	0.149090467	0.718587609	6.629	0.05348409	0.667796175
284	SLC26A11	10.410	0.0000183465	0.0455943394	10.57	0.000192328	0.061209929	9.652	0.001654666	0.51405755
285	SLC27A1	12.350	0.3705256019	1.2794620047	11.59	0.678347372	1.090074548	11.57	0.754243526	1.067276714
286	SLC27A2	6.954	0.1005232045	1.6269666607	7.566	0.334453558	1.256673081	8.17	0.878723903	0.968478809
287	SLC27A3	11.010	0.0880773159	0.6955078968	11.66	0.813150121	1.073443826	10.95	0.250243462	0.784130188
288	SLC27A4	11.650	0.0079374019	1.7320860445	11.07	0.358477402	1.279280344	11.53	0.026706391	1.587939978
289	SLC27A5	8.015	0.2228182403	0.7723416136	8.444	0.346509216	0.811796979	8.287	0.245222096	0.783338645
290	SLC27A6	5.577	0.5209371505	0.8299649962	3.418	0.641690967	1.105873304	3.834	0.542483503	0.880718718
291	SLC28A1	2.012	0.1141754684	0.6470397503	1.929	0.331543293	0.753071979	3.442	0.88754295	1.030023276
292	SLC28A2	6.886	0.1805695775	0.6869019925	4.737	0.857599726	1.038396096	4.553	0.961001835	1.010291368
293	SLC28A3	7.128	0.0002131760	9.1612716307	7.839	0.019501845	2.34931738	9.911	0.391976937	1.204234071
294	SLC29A1	11.920	0.0096650319	2.5248342948	11.17	0.228285455	2.310739066	12.59	0.580690764	1.122067462
295	SLC29A2	8.764	0.0195548161	2.1556884863	7.536	0.281104625	1.728616667	9.844	0.294565757	1.246207396
296	SLC29A3	9.158	0.0086437670	2.8903680390	8.656	0.003428553	82189950.71	9.914	0.034907003	1.55835437
297	SLC29A4	11.340	0.0003350066	0.1895338243	12	0.003452155	0.156963491	9.691	0.072707974	0.687973284
298	SLC30A1	12.080	0.0003954483	2.0717370625	10.41	0.006686637	79352924.36	11.76	0.007456912	1.751146723

299	SLC30A2	5.004	0.0175331121	2.2635473798	7.858	0.251608119	1.276083962	7.891	0.251608119	1.276083962
300	SLC30A3	4.513	0.1216626504	0.5247481589	2.198	0.968111487	0.989516238	3.244	0.931052699	0.982143683
301	SLC30A4	9.177	0.0374562480	0.6505680999	7.908	0.454087755	2.088447171	9.21	0.110293212	0.718115463
302	SLC30A5	11.730	0.0117699323	1.6919998396	10.88	0.411353417	1.518227734	11.7	0.019787811	1.630721176
303	SLC30A6	11.000	0.0030144875	1.9048144399	#N/A	#N/A	#N/A	11	0.003085513	1.912385271
304	SLC30A7	10.270	0.0000900657	94570816.634	10.78	0.000331849	6.461603374	11.42	0.23812254	1.284895331
305	SLC30A8	3.244	0.0266805847	4.2890038666	8.57	0.907131086	1.027734593	9.936	0.36808043	0.827777317
306	SLC30A9	11.700	0.0054143022	2.9095461483	12.32	0.012524384	1.733813855	12.08	0.373385417	1.202684698
307	SLC30A10	3.101	0.0025120303	2.5608303359	3.006	0.014857606	2.155204608	4.516	0.643360643	1.101592741
308	SLC31A1	10.690	0.0042299073	10.3395555920	10.47	0.005291671	80826744.59	11.49	0.351381585	1.215892172
309	SLC31A1P1	1.360	0.2421241288	1.3378321508	#N/A	#N/A	#N/A	0.5498	0.776344376	1.06445037
310	SLC31A2	8.959	0.0008641818	2.8819226640	8.861	0.00757913	2.352832671	9.756	0.158029912	1.352940592
311	SLC32A1	1.325	0.0134398210	0.5458926595	#N/A	#N/A	#N/A	0.9179	0.025697009	0.61188664
312	SLC33A1	11.060	0.0033976666	2.3436280027	11.5	0.021367143	1.624525391	11.38	0.012313302	1.681673306
313	SLC33A2	8.588	0.1477807450	0.6301296364	9.842	0.730417364	1.07769112	10.06	0.340406773	1.221791356
314	SLC34A1	0.361	0.0408937061	1.6241763899	#N/A	#N/A	#N/A	1.503	0.353219154	1.216106969
315	SLC34A2	6.250	0.0004971347	14.2664413056	8.948	0.026125428	1.866807409	10.03	0.079263278	1.488446439
316	SLC34A3	5.194	0.2224555612	1.4103987344	2.885	0.7237821	1.077303848	3.09	0.856031581	0.962955179
317	SLC35A1	9.884	0.0026747073	11.1847272140	10.95	0.014018075	1.668632753	10.93	0.02719765	1.586795592
318	SLC35A2	11.470	0.0002188070	2.3820202964	11.62	0.000152413	2.253820938	11.65	0.000343549	2.149846011
319	SLC35A3	10.440	0.0000618484	4.7120366021	9.823	4.76001E-05	19.32440074	11.09	0.007333544	1.771150934
320	SLC35A4	11.910	0.0097212005	5.2433072773	12.23	0.157951719	1.46124487	12.51	0.497794271	1.151436104
321	SLC35A5	10.520	0.0024985449	2.6625053875	10.55	0.004525065	2.430216458	10.86	0.014432438	1.69200149
322	SLC35B1	12.110	0.1147066864	1.4880821318	11.73	0.870491647	0.965715439	11.62	0.798922527	0.947915967
323	SLC35B2	12.140	0.0013664149	2.1857638347	11.69	0.018057156	2.687099	12.35	0.018544528	1.640472422
324	SLC35B3	10.830	0.0381738638	1.5338589932	9.999	0.005656589	4.593750021	10.69	0.114401718	1.391399193
325	SLC35B4	9.751	0.0416610644	1.8701028065	10.25	0.053158496	1.522932123	10.23	0.033919935	1.590327833
326	SLC35C1	11.780	0.0089077353	1.8945769347	11.18	0.010943013	2.818563316	12	0.241748374	1.288404244
327	SLC35C2	12.450	0.0094784845	1.7463752314	11.98	0.647017955	1.105322654	12.12	0.664770636	1.094640994
328	SLC35D1	11.280	0.0396330678	1.5667239924	#N/A	#N/A	#N/A	10.82	0.188870207	1.322555085

329	SLC35D2	10.800	0.0091724927	2.0975476391	#N/A	#N/A	#N/A	11.2	0.877544937	1.032663899
330	SLC35D3	1.902	0.0888542179	2.6268088609	2.961	0.337502951	1.425101097	5.171	0.827335811	1.046368611
331	SLC35D4	8.286	0.0005888576	3.8752411419	8.885	0.008319591	1.735531442	8.93	0.008308719	1.730549676
332	SLC35E1	12.230	0.0154351607	1.6970530024	12.72	0.002814918	2.029024414	12.29	0.017942757	1.642497817
333	SLC35E2	8.121	0.1148192606	0.4082943095	7.198	0.997838837	0.999419886	7.3	0.844180605	1.042008121
334	SLC35E3	10.280	0.0281183632	1.8458881949	10.69	0.384293953	1.556244455	9.832	0.814334449	1.050151051
335	SLC35E4	9.389	0.0245753737	1.5929626936	9.043	0.039156575	1.539755088	9.045	0.023070339	1.608075888
336	SLC35F1	7.044	0.0189756875	0.3205331811	7.671	0.059814271	0.28288942	5.971	0.22386515	0.776788816
337	SLC35F2	11.510	0.0000169266	2.4725334330	9.119	0.000268066	8.866947971	10.71	0.001561009	2.017582799
338	SLC35F3	9.210	0.0029730968	0.0910729529	4.056	0.402870294	1.627286357	6.838	0.754836232	0.936324251
339	SLC35F4	5.866	0.0182834207	0.2122455164	#N/A	#N/A	#N/A	3.189	0.613931574	0.900252761
340	SLC35F5	11.150	0.0003225874	3.5241745747	11.19	0.001467222	2.800880366	11.66	0.247222134	1.278766443
341	SLC35F6	11.350	0.0177752333	2.6302932086	11.27	0.051639672	2.985586721	11.89	0.181260215	1.320906191
342	SLC35G1	7.113	0.0270888448	4.2743481368	9.235	0.988136772	0.992411515	8.126	0.058489741	1.480887387
343	SLC35G2	8.919	0.0939863990	0.7016518605	7.116	0.241103133	1.967499196	8.845	0.330688464	0.814229956
344	SLC35G3	1.497	0.0666822818	0.3565404839	#N/A	#N/A	#N/A	0.273	0.619093375	0.861696209
345	SLC35G4	#N/A	#N/A	#N/A	#N/A	#N/A	#N/A	0.02621	0.003880197	4.810773745
346	SLC35G5	2.044	0.4107355482	0.8421622919	#N/A	#N/A	#N/A	2.226	0.420893467	0.844012895
347	SLC35G6	1.209	0.0573781758	0.6517655977	#N/A	#N/A	#N/A	0.872	0.143148482	0.734422725
348	SLC36A1	9.110	0.0000923554	95823975.378	9.673	0.005527394	3.088790071	10.65	0.614754211	1.116896085
349	SLC36A2	0.880	0.1405792807	1.4633738461	#N/A	#N/A	#N/A	0.3377	0.126388695	1.473612409
350	SLC36A3	1.237	0.0293348161	0.1502829271	#N/A	#N/A	#N/A	0.159	0.083753739	0.532332748
351	SLC36A4	9.940	0.1802909378	0.7080280991	#N/A	#N/A	#N/A	9.633	0.93311505	1.017640942
352	SLC37A1	10.580	0.0103330133	2.8246560043	9.735	0.428542992	3332715.752	11.54	0.478879336	1.15973551
353	SLC37A2	7.716	0.0000732158	7.6171074548	8.95	0.125310893	1.4528696	9.466	0.295147361	1.257257554
354	SLC37A3	10.920	0.0000658866	10.1864604086	10.79	0.002428572	6.61777957	11.44	0.008793605	1.748076338
355	SLC37A4	11.000	0.0455397195	1.9911721031	11.63	0.999898837	1.000026681	11.73	0.644725754	0.9086614
356	SLC38A1	12.010	0.0035616084	2.8309392797	10.97	0.006259016	79400760.88	12.71	0.074325135	1.458353974
357	SLC38A2	12.260	0.0013571864	12.4770426718	13.07	0.036537043	1.793682538	13.43	0.002499007	1.953370998
358	SLC38A3	9.548	0.2499289919	1.3040491837	7.085	0.314768923	1.236856097	7.427	0.386906397	1.198572415

359	SLC38A4	11.230	0.0061177834	0.1731989520	10.04	0.046629794	0.435987952	7.796	0.706519483	0.924670688
360	SLC38A5	7.338	0.0008116456	13.6334346198	8.941	0.17627389	1.485053462	10.9	0.403702965	1.192921247
361	SLC38A6	9.044	0.0043664689	1.8295540808	8.072	0.018795376	7.50703479	9.049	0.007125636	1.759771378
362	SLC38A7	9.678	0.0007176455	85538172.621	10.22	0.013658443	2.120974212	10.58	0.10837853	1.400311343
363	SLC38A8	1.791	0.6891513654	0.9169043775	5.611	0.278366532	0.530277863	2.19	0.606942849	0.898602139
364	SLC38A9	9.826	0.0415207343	1.6827269335	8.818	0.007431117	3.643426802	9.385	0.215615908	1.294933017
365	SLC38A10	12.770	0.0042391449	0.4539781227	13.72	0.565255805	0.886038921	13.57	0.985720137	0.996271554
366	SLC38A11	8.641	0.0051002959	0.5030561966	10.03	0.082266734	0.42107437	7.87	0.088410087	0.70271908
367	SLC39A1	12.500	0.0001272981	17.1906446972	#N/A	#N/A	#N/A	13.46	0.038833706	1.556407598
368	SLC39A2	2.421	0.9537148594	0.9879421220	5.264	0.319183781	1.446126543	2.499	0.877327085	1.032998835
369	SLC39A3	9.831	0.0107775498	0.5159118971	10.05	0.271431589	0.767339699	10.56	0.586103193	1.121364608
370	SLC39A4	11.170	0.0094854694	1.7531197922	10.17	0.075367096	1.649135379	11.34	0.010563797	1.72045716
371	SLC39A5	5.239	0.0164272026	2.5044507641	7.034	0.137014415	1.476550264	8.746	0.384135355	1.200562478
372	SLC39A6	12.170	0.0253085137	1.6101955937	11.76	0.592209124	1.121274763	11.87	0.416371261	1.184114918
373	SLC39A7	14.250	0.0011226160	2.2722998247	14.02	0.317427855	1.260052418	13.73	0.409553077	1.186882427
374	SLC39A8	9.516	0.0002407222	15.8814767985	11.04	0.168455095	1.3803814	10.58	0.00695939	1.771269459
375	SLC39A9	12.530	0.0374335741	1.5657643683	11.92	0.041153651	3.876574473	12.57	0.077107476	1.443741367
376	SLC39A10	11.000	0.0026067424	1.8935151976	9.869	0.00765934	3.22877868	10.97	0.008936617	1.745489252
377	SLC39A11	11.420	0.0020963076	2.2800156101	10.18	0.006523679	78873067.34	11.79	0.017419763	1.645373958
378	SLC39A12	1.523	0.0664834347	1.4677058769	#N/A	#N/A	#N/A	1.234	0.294227435	1.253115824
379	SLC39A13	12.670	0.1773174105	1.4472790548	11.51	0.463901414	1.200453268	12.08	0.83097207	1.045726069
380	SLC39A14	11.880	0.0003279710	89398029.949	11.88	0.000327971	89398029.95	13.25	0.135173471	1.370860082
381	SLC40A1	13.650	0.2367541223	1.2876366729	12.65	0.068610511	1.839388594	13.72	0.308439412	1.241862581
382	SLC41A1	11.190	0.1657161205	1.3771328946	10.84	0.223559598	1.439307514	11.28	0.724190213	1.076365152
383	SLC41A2	10.920	0.0110553178	1.8123848325	9.586	0.034455826	3.244732883	11.09	0.047297082	1.548038795
384	SLC41A3	11.670	0.0579935954	1.5345476204	#N/A	#N/A	#N/A	11.81	0.228617581	1.288349668
385	SLC42A1	3.501	0.3275650823	0.6088964522	#N/A	#N/A	#N/A	1.149	0.128053961	1.372185236
386	SLC42A2	1.581	0.0643409699	1.5655829347	2.355	0.703765811	1.082316607	2.372	0.641967107	1.101418163
387	SLC42A3	2.809	0.0007846576	7.7183757588	6.943	0.026248201	1.716236004	5.429	0.019230392	1.632769116
388	SLC43A1	10.340	0.3955772744	0.8376443821	11.64	0.653218795	1.1557203	10.4	0.823081571	1.047760699

389	SLC43A2	11.570	0.0003581678	0.4538462834	12.64	0.06415077	0.187651172	11.45	0.001851774	0.518211672
390	SLC43A3	9.649	0.0095205862	5.3062668373	10.56	0.001813242	3.981628109	11.54	0.253533403	1.278481017
391	SLC44A1	11.830	0.0002553264	91928262.470	12.12	0.000228513	16.03155021	13.46	0.020277205	1.65152192
392	SLC44A2	14.240	0.0004283366	2.0796078693	12.59	0.003505356	4.82740235	13.8	0.00375964	1.885585826
393	SLC44A3	10.250	0.0128993286	2.3363927300	11.06	0.104164487	1.429235241	11.11	0.14226326	1.372067788
394	SLC44A4	14.030	0.0273654962	1.5960324630	10.94	0.004150324	10.39263057	13.64	0.335944953	1.240742841
395	SLC44A5	8.922	0.0891480021	1.4529798487	8.293	0.995449088	1.001211613	7.265	0.259924324	0.791191198
396	SLC45A1	8.788	0.0008150262	0.2100167835	6.108	0.837996214	0.886847099	7.836	0.006755424	0.56044612
397	SLC45A2	4.220	0.0012629841	0.2166592767	2.256	0.246759717	0.775284562	2.723	0.075589704	0.690866234
398	SLC45A3	8.833	0.0001438439	93015550.133	#N/A	#N/A	#N/A	10.89	0.131858314	1.377231778
399	SLC45A4	10.730	0.0105221974	2.6420273390	11.4	0.112875692	1.409146347	11.57	0.129776276	1.369041842
400	SLC46A1	11.310	0.0000658213	0.0532746088	11.93	0.014280252	3.35429E-08	10.53	0.535573383	0.878869375
401	SLC46A2	2.406	0.2068848648	1.4583202369	1.894	0.14886736	1.655282827	3.504	0.824646713	1.048389341
402	SLC46A3	10.720	0.0292445105	1.7564657359	#N/A	#N/A	#N/A	11.01	0.448461449	1.17518652
403	SLC47A1	5.517	0.0005376324	3.4385865246	6.099	0.010411486	2.059424932	6.979	0.798403047	1.055323259
404	SLC47A2	4.175	0.4318037094	0.6975043365	1.754	0.664363737	0.898988023	2.555	0.224058292	0.776652556
405	SLC48A1	11.370	0.0044337358	2.5432511020	#N/A	#N/A	#N/A	10.66	0.831304175	1.045542672
406	SLC49A1	10.420	0.0132417666	0.4824767193	10.29	0.12053466	0.683618009	9.972	0.852034853	1.039641342
407	SLC49A2	6.666	0.0011382247	12.8860426758	7.097	0.008590145	3.570248825	8.815	0.564826732	1.131228019
408	SLC49A3	7.489	0.0047387103	4.5913970980	7.907	0.007325394	3.268451362	9.401	0.320808848	1.229867769
409	SLC49A4	9.609	0.0000100554	12.1567179002	9.616	1.00554E-05	12.1567179	10.31	0.009406368	1.769265134
410	SLC50A1	11.520	0.0006529818	2.4282660481	10.64	0.011784123	3.995567323	11.88	0.11939847	1.381052615
411	SLC51A	5.935	0.0427411937	3.1127166187	7.7	0.863358188	0.941182911	6.939	0.779115105	0.943071601
412	SLC51B	3.227	0.0709196666	1.4771968956	1.631	0.892426138	0.955592496	3.406	0.169525721	1.334922906
413	SLC52A1	3.038	0.0013542491	4.5322197502	3.259	0.008820415	2.578371355	4.692	0.613546067	0.900273094
414	SLC52A2	11.930	0.0257872776	1.6673061706	11.86	0.088853018	1.48746594	12.15	0.080015552	1.447810431
415	SLC52A3	8.388	0.0003820440	5.1722085014	7.34	0.000603008	14.25056071	9.683	0.011159093	1.744702907
416	SLC53A1	11.590	0.0150292337	1.7154328880	10.81	0.018025894	3.688242759	11.64	0.039274544	1.559030477
417	SLC54A1	10.220	0.2295658721	0.7630690307	11.65	0.38662001	1.845479567	10.51	0.858139407	1.037900966
418	SLC54A2	11.770	0.2249778929	0.7724367113	12.55	0.169843178	1.579040508	11.71	0.125651347	0.72443446

419	SLC54A3	0.443	0.3192568111	1.3948415693	#N/A	#N/A	#N/A	0.1104	0.319256811	1.394841569
420	SLC55A1	11.910	0.1153248388	1.4052191418	#N/A	#N/A	#N/A	11.65	0.331689961	1.224076693
421	SLC55A2	8.657	0.0002307706	2.1454245256	10.18	0.004417141	2.360890016	8.614	0.000569923	2.047351342
422	SLC55A3	11.880	0.0128886565	1.6764791830	12.14	0.17554787	1.399638176	11.7	0.585766052	1.12088649
423	SLC56A1	11.110	0.3823747717	1.2036387263	11.02	0.708873005	1.084513625	11.26	0.434725728	1.177569191
424	SLC56A2	9.981	0.0000296888	3.0221729887	7.394	0.130015055	4.083868451	9.067	0.59333627	0.894843755
425	SLC56A3	11.270	0.0001287022	94076361.426	11.09	0.000932364	85640017.44	12.59	0.02773549	1.593440857
426	SLC56A4	10.590	0.0892134205	1.4871800447	11.54	0.994138678	0.995686384	10.14	0.421153201	1.182063575
427	SLC56A5	9.670	0.0000366757	0.4314532376	11.01	0.012185037	3.5347E-08	9.817	0.001229552	0.506136972
428	SLC57A1	10.510	0.0018687896	3.4401153199	10.1	0.016252131	3.726722074	11.02	0.280924544	1.254437712
429	SLC57A2	11.270	0.0000512747	10.4694977220	10.84	0.0005293	86984930.48	11.79	0.028481324	1.580048829
430	SLC57A3	6.835	0.0005719922	82987037.329	8.213	0.009240753	2.039292	8.636	0.005218454	1.818528115
431	SLC57A4	11.480	0.1716332318	1.3310600797	#N/A	#N/A	#N/A	11.26	0.600523158	1.117786535
432	SLC57A5	10.880	0.0033346029	3.0160897354	10.47	0.009984056	5.240237887	11.35	0.737976469	1.07226561
433	SLC57A6	2.417	0.0743904402	2.4205227097	5.361	0.616060939	1.110916448	5.276	0.814090361	1.05043246
434	SLC58A1	11.970	0.0001618536	17.4742954398	13.26	0.004008397	2.216541999	12.7	0.001767193	1.944971558
435	SLC58A2	12.870	0.0156207591	0.3090642387	9.128	0.744112823	1.211260228	11.61	0.077244393	0.693011616
436	SLC59A1	7.384	0.0613378687	3.5322194926	#N/A	#N/A	#N/A	9.569	0.43415425	1.179171899
437	SLC59A2	3.186	0.0013720856	4.4777773032	4.277	0.01653398	1.659161572	4.335	0.021393755	1.615820664
438	SLC60A1	10.830	0.0209205362	1.6279634915	10.68	0.039185089	1.543221702	10.12	0.181867496	1.319550751
439	SLC61A1	10.660	0.0009969955	4.0810918255	11.29	0.048563875	1.525930059	11.32	0.082336688	1.438754227
440	SLC62A1	13.800	0.0985831196	0.3256521217	10.96	1	NA	12.85	0.45109512	0.854741001
441	SLC63A1	12.400	0.2422541515	1.4548659647	11.19	0.348757608	1.254646299	11.64	0.303641174	1.239698008
442	SLC63A2	13.470	0.1608395913	1.5996364166	10.02	0.027698611	2.679049827	11.65	0.187669635	1.328976314
443	SLC63A3	8.362	0.0218769420	2.1315075907	5.463	0.05613754	2.579658618	7.072	0.55214078	1.13505135
444	SLC64A1	11.730	0.0008893933	13.4630029441	11.23	0.001189377	85859692.79	12.53	0.31952236	1.23082136
445	SLC65A1	11.200	0.0007608423	3.4830374641	12.46	0.229110686	1.320937171	11.89	0.009859894	1.711622548
446	SLC65A2	8.161	0.1041804443	0.6339746528	9.494	0.961585135	0.987650006	10.61	0.333324296	0.811375773

*Abbreviations: HR – Hazard Ratio, p – p-value, cutoff – optimal threshold value for grouping, outcome – overall survival, dis – disease-specific survival, mean – mean expression-based grouping.
