# Supplementary material for: The Solute Carrier Superfamily as Therapeutic Targets in Pancreatic Ductal Adenocarcinoma
Source: Genes (Basel). 2025 Apr 18;16(4):463. doi: 10.3390/genes16040463 (PMC12027052; doi:10.3390/genes16040463)
Supplement: Supplementary file 1 [file genes-16-00463-s001.zip › Table S5.pdf]

Table S4. Disease specific survival analysis by Cutoff finder

No.	Name	outcome_cutoff	outcome_p	outcome_HR	dis_cutoff	dis_p	dis_HR	mean_cutoff	mean_p	mean_HR
1	SLC1A1	10.69	0.026417939	1.78477771	8.797	0.169092352	1.795079841	10.97	0.156217864	1.406704458
2	SLC1A2	5.587	0.00196354	0.488424215	8.024	0.920139543	1.02761796	6.668	0.165052045	0.721516248
3	SLC1A3	10.78	0.049739891	1.671078646	8.025	0.113270007	1.871046057	9.75	0.066582128	1.555923523
4	SLC1A4	11.44	0.021544186	0.386311968	9.792	0.217447904	0.490090668	10.88	0.783638326	0.937459749
5	SLC1A5	12.48	0.003584456	2.85548341	11.25	0.005619409	84076283.66	12.91	0.998318128	1.000495887
6	SLC1A6	2.143	0.067685176	1.669584129	#N/A	#N/A	#N/A	1.015	0.64129162	1.116733687
7	SLC1A7	7.582	0.055308155	0.526145758	6.06	0.751391561	1.102182016	6.875	0.321985438	0.791596956
8	SLC2A1	12.44	0.000133424	5.01739049	13.64	0.016746769	1.783780447	13.73	0.004561872	1.983798726
9	SLC2A2	1.111	0.230598099	2.305923646	4.245	0.863318835	1.052571637	6.413	0.757838239	0.929814286
10	SLC2A3	11.21	0.003014589	3.094664271	#N/A	#N/A	#N/A	12.1	0.024910324	1.694066533
11	SLC2A3P1	2.729	0.352739173	1.318452763	#N/A	#N/A	#N/A	0.8263	0.451716511	1.251265362
12	SLC2A3P2	3.618	0.14877088	0.259345395	#N/A	#N/A	#N/A	1.542	0.762722353	1.073744998
13	SLC2A3P4	3.384	0.111626021	0.403626728	#N/A	#N/A	#N/A	1.926	0.928171623	0.979040837
14	SLC2A4	6.396	0.184098415	0.692994283	#N/A	#N/A	#N/A	6.084	0.380671052	0.810952198
15	SLC2A5	7.946	0.003485812	2.61879328	6.213	0.027672339	6.823794425	8.658	0.322201276	1.269926161
16	SLC2A6	8.369	0.173130909	0.660945254	9.13	0.841447123	0.951834566	9.497	0.948560371	0.984969735
17	SLC2A7	1.787	0.30128888	0.590602197	#N/A	#N/A	#N/A	0.4785	0.51872915	0.844198116
18	SLC2A8	9.156	8.01582E-05	0.343455779	9.92	0.155272059	0.716807736	9.916	0.095706892	0.676920867
19	SLC2A9	8.835	0.135087572	0.24890766	6.833	0.003042486	4.97421975	7.847	0.924495326	1.022638554
20	SLC2A10	9.545	0.000461157	90498101.47	9.866	0.007483373	4.31083611	11.18	0.071198534	1.545460046
21	SLC2A11	8.932	0.012407276	0.499346732	#N/A	#N/A	#N/A	9.532	0.344884707	0.800954214
22	SLC2A12	6.273	0.02755351	6.795622785	5.935	0.039373658	26783585.82	8.143	0.578428804	1.14067248
23	SLC2A13	10.85	0.230517859	0.65447398	8.672	0.834785259	1.113303118	10.15	0.999427154	1.00016819
24	SLC2A14	1.19	0.005777427	10.07262889	3.93	0.305516771	1.278195959	4.205	0.268544758	1.299056523
25	SLC3A1	14.39	0.114893868	0.233173925	9.367	0.30334998	1.358334271	11	0.341931282	1.257474585
26	SLC3A2	14.15	0.352855448	1.370576948	14.48	0.28456405	0.355834757	13.38	0.582341335	1.137416358
27	SLC4A1	1.408	0.026821418	0.575794203	#N/A	#N/A	#N/A	1.18	0.124568827	0.693707548
28	SLC4A2	12.7	0.021104076	2.094917005	12.88	0.02926587	1.782930188	13.09	0.497284563	1.175436907

29	SLC4A3	9.065	0.060209911	0.645281442	#N/A	#N/A	#N/A	9.208	0.06926216	0.654305261
30	SLC4A4	15.38	0.035939906	3.75642E-08	11.31	0.224753212	1.40791199	12.41	0.829440479	1.052284427
31	SLC4A5	7.671	0.003706116	0.353239199	6.231	0.782815499	1.0855997	7.081	0.162729832	0.7164656
32	SLC4A7	9.906	0.001396314	3.161297189	9.087	0.050645575	3.010000929	10.5	0.836303364	1.050114962
33	SLC4A8	7.332	0.001744859	0.457660067	10.26	0.015048462	0.259896507	8.356	0.005640593	0.518030977
34	SLC4A9	1.447	0.063801256	0.617405353	2.356	0.817089352	0.947051834	2.647	0.882541393	0.96593282
35	SLC4A10	3.429	0.19452064	1.936634315	6.295	0.314140622	0.789483908	6.116	0.374903466	0.812538699
36	SLC4A11	11.77	0.236123329	0.548356386	11.25	0.884067251	0.957534043	9.78	0.631182338	1.119056454
37	SLC5A1	12.59	0.079083781	1.57209971	8.688	0.041224066	3.144396496	11.27	0.957590432	1.012810536
38	SLC5A2	5.807	0.203289623	0.741327038	3.896	0.69535335	0.833837898	6.163	0.262109712	0.769225663
39	SLC5A3	10.46	0.003275239	2.078787505	9.469	0.024114578	3.051055907	10.55	0.029363375	1.678976605
40	SLC5A4	4.774	0.17111114	0.685922136	2.897	0.750741448	0.904539295	4.098	0.522655114	0.860964563
41	SLC5A5	5	0.787373957	0.917928268	7.666	0.547997821	0.651926728	3.757	0.905831649	0.972547132
42	SLC5A6	10.31	0.00947458	3.530126407	10.11	0.015460815	3.809349057	10.83	0.657229027	0.900652538
43	SLC5A7	0.4644	0.046891992	2.072777054	#N/A	#N/A	#N/A	3.432	0.725624199	0.920956909
44	SLC5A8	4.867	0.148570783	0.436904383	3.648	0.805776283	0.916151347	1.914	0.66643346	1.107196438
45	SLC5A9	8.545	0.089639275	0.515211381	4.864	0.212274014	0.666292264	6.9	0.783793415	0.937330689
46	SLC5A10	5.145	0.070038425	0.586756715	3.171	0.372673949	0.7386993	4.598	0.860151324	0.95955027
47	SLC5A11	1.471	0.005800285	0.499127626	2.681	0.445157703	0.834865398	2.637	0.556427298	0.870871679
48	SLC5A12	3.359	0.050564277	0.624850162	2.662	0.179988154	0.730120011	3.225	0.053077165	0.629881351
49	SLC6A1	8.6	0.068617585	0.193402811	#N/A	#N/A	#N/A	7.068	0.08552921	1.50965934
50	SLC6A2	1.452	0.27834075	0.775418534	3.74	0.726257921	1.126569885	1.957	0.989539664	0.996932577
51	SLC6A3	0.3754	0.082453191	2.066473016	#N/A	#N/A	#N/A	2.693	0.373139645	1.23392921
52	SLC6A4	8.174	0.03307086	0.303844759	10.66	0.135564353	3.90764E-08	5.913	0.397260438	0.82008947
53	SLC6A5	1.239	0.14786261	0.436073591	#N/A	#N/A	#N/A	0.1634	0.567910915	0.807348439
54	SLC6A6	11.85	0.003287599	4.883846735	#N/A	#N/A	#N/A	12.91	0.255874385	1.31839261
55	SLC6A7	3.17	0.101499526	0.667409659	2.6	0.560914022	0.835942299	4.006	0.657222596	1.109714732
56	SLC6A8	11.34	0.118295277	1.606196139	14.6	0.453758983	0.587855044	12.3	0.290101274	1.281962425
57	SLC6A9	7.842	0.003621013	10.54581138	8.043	0.028098816	2.672966444	8.915	0.731277364	1.084402564
58	SLC6A10P	4.103	0.194400253	0.406459393	#N/A	#N/A	#N/A	1.638	0.585240937	1.136872105

59	SLC6A11	4.981	0.033110373	1.93733851	4.07	0.891987501	1.038464971	3.114	0.216786697	1.335795054
60	SLC6A12	6.101	0.039031584	1.807116642	4.453	0.414628901	1.519190855	6.855	0.798284492	1.061791702
61	SLC6A13	1.883	0.00953227	0.537767178	2.24	0.058997167	0.640746532	2.688	0.041779885	0.620348826
62	SLC6A14	12.02	0.000568064	2.205013578	6.765	0.001126741	12.93209992	10.68	0.005189675	2.020425745
63	SLC6A15	1.17	0.067293959	2.041648277	#N/A	#N/A	#N/A	3.321	0.58676749	1.136462469
64	SLC6A16	6.921	0.002218152	0.266437888	4.816	0.287424262	0.740656575	5.863	0.234250252	0.755748216
65	SLC6A17	10.43	0.014357364	0.303224837	9.214	0.379457148	0.805138709	8.161	0.820907941	0.948197505
66	SLC6A18	1.596	0.127132152	0.499075114	#N/A	#N/A	#N/A	0.492	0.816259052	1.063056687
67	SLC6A19	8.726	0.053137914	0.447000369	7	0.279673628	0.773165939	6.197	0.585513599	0.879633944
68	SLC6A20	9.98	0.002375793	2.982322493	8.324	0.001601042	7.17512726	10.69	0.195101664	1.392257481
69	SLC7A1	11.74	0.002585659	2.79936241	12.21	0.788083074	1.065307396	12.17	0.412540381	1.215089562
70	SLC7A2	13.2	0.078059583	0.302410864	#N/A	#N/A	#N/A	11.09	0.85610038	1.043598513
71	SLC7A3	5.551	0.081229731	1.69635565	3.267	0.6524978	0.894967618	3.839	0.711968186	0.916370397
72	SLC7A4	7.738	0.935274226	1.020595658	7.967	0.384869606	0.811687443	7.966	0.384869606	0.811687443
73	SLC7A5	11.12	0.014931543	2.424131879	14.08	0.159245614	2.034549208	11.93	0.061367197	1.548756613
74	SLC7A5P1	3.822	0.425811226	0.691520664	#N/A	#N/A	#N/A	2.529	0.161995791	1.396001797
75	SLC7A6	9.658	0.06329265	2.067802766	10.23	0.587013056	1.148965917	10.42	0.655594917	1.110662025
76	SLC7A7	10.78	0.001338406	3.638580459	9.873	0.00524843	5.956942051	11.52	0.202534182	1.363052631
77	SLC7A8	11.62	0.022190434	0.588145625	12.97	0.045790562	0.324910006	11.8	0.090599218	0.669970811
78	SLC7A9	2.81	0.232656953	0.641507083	8.695	0.191514296	0.290332635	5.21	0.998809892	0.999649762
79	SLC7A10	1.866	0.008713388	0.519471431	2.769	0.044181474	0.566709786	2.129	0.024514922	0.561617565
80	SLC7A11	7.354	0.001358889	87479912.99	#N/A	#N/A	#N/A	9.739	0.228031713	1.327555485
81	SLC7A13	1.89	0.673906008	0.740064802	#N/A	#N/A	#N/A	0.3747	0.273881971	1.363651313
82	SLC7A14	8.642	0.0070925	0.272580575	5.361	0.196133293	0.728406475	6.17	0.296886013	0.782558107
83	SLC7A15P	#N/A	#N/A	#N/A	#N/A	#N/A	#N/A	#N/A	#N/A	#N/A
84	SLC8A1	9.962	0.130316355	0.689627914	9.282	0.080492036	0.653949989	9.642	0.208236654	0.745465093
85	SLC8A2	9.284	0.039343108	0.314901347	7.258	0.859934187	0.959413244	6.989	0.943782598	0.983471208
86	SLC8A3	7.124	0.005694078	0.102539445	4.531	0.099984873	0.667932188	5.197	0.527665228	0.862215434
87	SLC8B1	10.78	0.004768723	6.043779811	#N/A	#N/A	#N/A	11.65	0.198484384	1.362245849
88	SLC9A1	11.43	0.00031109	96271634.21	11.2	0.001134252	90470726.77	12.64	0.567935024	1.143982405

89	SLC9A2	5.903	0.008081001	3.245460481	4.829	0.064463231	2.521575506	8.174	0.709910346	1.093156889
90	SLC9A3	3.441	0.419415393	0.774637929	7.468	0.501199856	0.853691577	7.472	0.501199856	0.853691577
91	SLC9A3P1	#N/A	#N/A	#N/A	#N/A	#N/A	#N/A	0.03815	0.813417408	0.844137768
92	SLC9A3P2	0.3209	0.677123821	0.782593813	#N/A	#N/A	#N/A	0.1294	0.677123821	0.782593813
93	SLC9A3P3	1.533	0.440001003	1.388102337	#N/A	#N/A	#N/A	0.2549	0.933237381	0.974663898
94	SLC9A4	1.516	0.000218551	9.112755821	6.134	0.477333738	1.181067701	5.834	0.228091401	1.327316869
95	SLC9A5	5.702	0.107163858	0.670317933	5.916	0.375026459	0.805038971	6.345	0.048220824	0.628762027
96	SLC9A6	10.69	0.146568188	1.403232919	10.21	0.322332842	1.398799481	10.56	0.487630557	1.180521787
97	SLC9A7	11.74	0.119616562	1.583431223	12.7	0.024056352	3.566490156	11.01	0.530872127	0.862880052
98	SLC9A7P1	5.258	0.030246096	0.546146097	4.277	0.941052843	0.96261922	6.098	0.948088669	0.984675154
99	SLC9A8	11.93	0.066315372	0.290162094	11.17	0.889423159	1.033188639	11.16	0.726279024	1.085738338
100	SLC9A9	9.552	0.226411457	0.683128504	7.378	0.489305959	1.317388848	8.785	0.986220399	1.004090821
101	SLC9B1	4.584	7.28279E-05	0.293058693	3.217	0.630174828	0.834524019	4.217	0.0045291	0.513708092
102	SLC9B2	11.13	0.071299245	1.528171481	#N/A	#N/A	#N/A	10.7	0.37100941	1.235763491
103	SLC9C1	3.822	0.094676237	0.582430528	2.474	0.971707862	1.009524201	2.976	0.818757736	0.947308123
104	SLC9C2	4.748	0.203340674	0.479866545	#N/A	#N/A	#N/A	2.497	0.282882937	1.288098458
105	SLC10A1	2.752	0.050883141	0.585295951	#N/A	#N/A	#N/A	2.023	0.249915185	0.763325754
106	SLC10A2	1.368	0.000836728	2.183699617	#N/A	#N/A	#N/A	1.547	0.005699133	1.911735295
107	SLC10A3	10.63	0.001364195	12.56810754	10.6	0.003022885	10.95083672	11.56	0.092502706	1.491979225
108	SLC10A4	3.534	0.031668589	0.59820072	2.233	0.456008663	0.813236383	3.365	0.107879061	0.686264091
109	SLC10A5	7.009	0.123074672	0.239560255	6.148	0.343427435	0.760026456	5.7	0.081308076	0.664366533
110	SLC10A6	5.007	0.164481405	0.646101256	2.218	0.875664396	0.929909384	4.307	0.424038515	1.206126492
111	SLC10A7	8.972	0.005341805	2.763444896	8.177	0.491216212	1.630865625	9.373	0.048697689	1.601542518
112	SLC11A1	8.797	0.000932988	5.71028547	10.04	0.06811639	1.602190504	10.56	0.123271196	1.446943007
113	SLC11A2	11.46	0.070859586	1.836283501	12.57	0.939388275	1.056092639	11.74	0.938221157	0.981920515
114	SLC12A1	3.211	0.001395581	0.300328074	4.568	0.008783149	0.111733029	2.238	0.021649649	0.58397809
115	SLC12A2	11.15	0.000223572	15.97802803	11.28	0.00202919	5.178708755	12.63	0.667595757	1.107195353
116	SLC12A3	1.492	0.310750361	0.718284743	2.752	0.804008023	1.071472584	3.562	0.670783895	1.105618543
117	SLC12A4	11.4	0.000198884	9.122174661	11.19	0.000356278	8.562774335	11.97	0.41021782	1.221191569
118	SLC12A5	7.163	0.006608427	0.352064151	8.575	0.004354009	1.19329E-08	6.233	0.196229175	0.736789924

119	SLC12A6	10.55	0.001681448	4.464400501	10.07	0.037543522	3.20721044	11.12	0.768918595	1.072500336
120	SLC12A7	12.82	0.016980428	2.095067087	11.66	0.63751152	1.402435996	13.21	0.776853482	1.068844339
121	SLC12A8	10.07	0.001895207	3.914227434	9.067	0.008142667	82607188.27	10.69	0.325377257	1.261419485
122	SLC12A9	10.82	0.128022594	0.568429318	11.87	0.615544217	1.129015961	11.95	0.93153061	1.020430118
123	SLC13A1	3.492	0.597154353	1.252989055	#N/A	#N/A	#N/A	1.152	0.892703276	1.032247562
124	SLC13A2	8.942	0.130560986	0.502538388	5.446	0.336421088	1.258389821	5.819	0.217164673	1.340720635
125	SLC13A3	6.436	0.00422139	3.531277083	6.588	0.0175685	2.521699807	7.61	0.56051736	1.150517189
126	SLC13A4	7.553	0.047367377	3.79928E-08	5.133	0.80287818	1.082324389	6.103	0.97394076	1.007745111
127	SLC13A5	12.2	0.018245906	2.196352151	7.337	0.019848067	1.752731486	7.912	0.027110004	1.686358052
128	SLC14A1	5.233	0.359115104	0.722250502	9.361	0.392738507	1.298326959	7.79	0.651008071	0.899401854
129	SLC14A2	3.655	0.088362815	0.513379439	#N/A	#N/A	#N/A	2.439	0.826645346	0.949925421
130	SLC15A1	11.63	0.046633914	0.17093156	6.741	0.135534646	1.983102376	9.534	0.797363938	1.064456832
131	SLC15A2	10.14	0.301274103	0.71364561	8.357	0.703188003	1.099126175	8.865	0.301572424	1.28305462
132	SLC15A3	9.172	0.007079258	9.320840631	10.64	0.086992322	1.833824151	11.41	0.955514252	1.013400139
133	SLC15A4	11.36	0.017603581	1.77527276	9.938	0.166587048	2.217088479	11.02	0.319784211	1.266757561
134	SLC16A1	10.97	0.001362433	2.586875633	#N/A	#N/A	#N/A	11.37	0.223707782	1.337663793
135	SLC16A2	10.27	0.060468934	1.771928167	9.464	0.693376446	1.200894959	10.65	0.496502051	0.851383106
136	SLC16A3	10.61	0.001290657	88907463.85	11.11	0.007284503	5.607137686	13.21	0.473782293	1.184212062
137	SLC16A4	7.074	0.014693307	7.970134233	8.551	0.166530464	1.636556757	10.04	0.312231763	1.27920535
138	SLC16A5	12.31	0.049627357	1.933898686	7.612	0.005995201	5.819270135	10.35	0.414795335	1.214636892
139	SLC16A6	6.956	0.170240649	1.517739015	5.697	0.469453982	1.527348528	7.652	0.794800017	1.062972853
140	SLC16A7	10.66	0.179121445	1.371433011	#N/A	#N/A	#N/A	10.36	0.69685886	1.097171878
141	SLC16A8	4.236	0.252603989	0.752502003	7.529	0.905081447	1.127730455	4.919	0.380261251	0.814140598
142	SLC16A9	10.02	0.027904286	0.147965064	10.87	0.333674369	0.39090754	8.075	0.933926125	0.980709018
143	SLC16A10	9.133	0.00295092	1.996305846	8.336	0.285880052	1.292673264	8.562	0.518584029	1.164443908
144	SLC16A11	7.412	3.6191E-05	0.219937224	5.175	0.042152633	0.579439521	6.389	0.095512134	0.675084585
145	SLC16A12	10.53	0.095026745	0.386370587	7.227	0.724437859	0.920177628	7.293	0.664982875	0.903116676
146	SLC16A13	7.248	0.041964679	3.119129377	7.3	0.090506957	1.938536188	7.986	0.426960874	0.829923243
147	SLC16A14	7.689	0.057137686	1.896194391	7.358	0.066487262	2.49952324	8.365	0.664639727	1.108738785
148	SLC17A1	1.974	0.447910804	1.194566833	#N/A	#N/A	#N/A	1.554	0.88993584	1.03299689

149	SLC17A2	2.616	0.026144854	0.145378017	#N/A	#N/A	#N/A	0.6197	0.723333989	0.918067882
150	SLC17A3	3.353	0.110901615	0.3353023	#N/A	#N/A	#N/A	1.119	0.956100498	1.013020894
151	SLC17A4	9.892	0.131674534	0.572255131	5.065	0.6652866	0.884449305	7.29	0.974853762	1.007561444
152	SLC17A5	10.24	0.002616443	83888492.69	11.75	0.338453257	1.339430602	11.21	0.802302064	1.060524398
153	SLC17A6	3.823	0.192976917	1.358558722	#N/A	#N/A	#N/A	3.496	0.280488204	1.290757238
154	SLC17A7	6.397	0.14341183	1.409299449	4.002	0.332846549	1.757710305	6.161	0.244953559	1.322857805
155	SLC17A8	3.516	0.183326868	1.640770923	#N/A	#N/A	#N/A	1.469	0.59253864	0.881688894
156	SLC17A9	12.77	0.028558273	0.236309269	8.78	0.108839758	2.262723179	10.8	0.543055154	0.867072571
157	SLC18A1	3.866	0.007354492	0.275647924	5.941	0.200588623	0.411246358	2.448	0.74594509	0.926866741
158	SLC18A2	5.449	0.054022424	2.383125848	6.019	0.576395898	1.200792336	7.257	0.384653265	0.815280692
159	SLC18A3	6.072	0.022944927	0.283536191	#N/A	#N/A	#N/A	3.407	0.337446891	0.798875549
160	SLC18B1	11.06	0.13845009	0.251555208	9.432	0.274149074	1.654625086	10.25	0.676181573	0.906849822
161	SLC19A1	9.198	0.003217364	4.09654188	#N/A	#N/A	#N/A	10.05	0.929374971	1.021226031
162	SLC19A2	9.47	0.000973781	86762525.27	9.093	0.002535087	85478773.05	10.51	0.298685289	1.278889604
163	SLC19A3	9.443	0.038486567	0.579495423	#N/A	#N/A	#N/A	8.645	0.171873208	0.726750443
164	SLC20A1	10.37	0.001261204	90508245.4	11.19	0.003630267	6.30664803	12.57	0.176758272	1.374178297
165	SLC20A2	11.43	0.020845616	2.798662571	10.76	0.051261029	3.695919822	11.94	0.19257356	1.362705445
166	SLCO2A1	11.98	0.196470466	0.739103809	11.64	0.387603068	0.810962598	12.03	0.277379486	0.77564207
167	SLCO4A1	7.686	0.003376404	10.86945959	10.27	0.283507925	1.286839081	10.32	0.283507925	1.286839081
168	SLCO5A1	7.065	0.021527857	0.579685479	7.89	0.206938274	0.685370359	7.104	0.07956946	0.659660297
169	SLCO3A1	10.21	0.001453275	12.33603012	9.934	0.004004068	10.37656351	11.21	0.109968703	1.480478522
170	SLCO6A1	1.009	0.069549537	0.438114014	#N/A	#N/A	#N/A	0.2509	0.139393154	0.576393023
171	SLCO1A2	5.165	0.347020365	0.715621727	#N/A	#N/A	#N/A	3.226	0.984986633	0.995592966
172	SLCO2B1	12.83	0.181986773	1.385703393	12.04	0.924498816	0.977893759	12	0.992136204	1.002332877
173	SLCO1B1	2.387	0.070141502	1.546801714	#N/A	#N/A	#N/A	1.718	0.545823037	1.154503749
174	SLCO1B3	9.698	0.004764195	2.273591456	6.947	0.050093158	1.584159285	5.655	0.023319905	1.710182739
175	SLCO4C1	6.071	0.00601887	0.522314641	5.088	0.709845915	0.910464563	5.904	0.016214866	0.570933634
176	SLCO1C1	5.562	0.237285048	0.680594325	3.803	0.244062004	0.746255643	4.522	0.714081864	1.089935823
177	SLC22A1	3.444	0.419148466	0.819301452	2.24	0.584109099	0.791927584	4.008	0.964065223	0.989476543
178	SLC22A2	1.341	0.333824351	0.783262147	5.939	0.548567288	0.551222385	2.323	0.75639003	1.075659039

179	SLC22A3	7.632	0.005317791	10.01205961	7.451	0.011403337	8.488287752	9.972	0.46491479	1.189566944
180	SLC22A4	6.618	0.056858973	2.20633104	6.033	0.931671629	1.051928583	7.346	0.562184808	0.872992961
181	SLC22A5	10.58	0.000295785	0.188939504	#N/A	#N/A	#N/A	10.04	0.571112029	0.875630915
182	SLC22A6	#N/A	#N/A	#N/A	#N/A	#N/A	#N/A	0.1026	0.848402988	0.906296173
183	SLC22A7	1.409	0.029115439	0.567619386	#N/A	#N/A	#N/A	1.068	0.389443467	0.813862104
184	SLC22A8	0.8054	0.553743269	0.760495986	#N/A	#N/A	#N/A	0.07323	0.328062338	0.637770054
185	SLC22A9	2.103	0.004559765	0.516311264	2.733	0.104704726	0.679751664	2.71	0.073628969	0.654186368
186	SLC22A10	1.346	0.005593326	0.437858939	#N/A	#N/A	#N/A	0.8741	0.241187799	0.756183807
187	SLC22A11	6.588	0.668757387	1.174263955	2.669	0.471902749	1.332173141	4.763	0.36861611	0.80878282
188	SLC22A12	1.453	0.085644571	0.377128534	#N/A	#N/A	#N/A	0.3085	0.734455913	0.901276346
189	SLC22A13	2.07	0.228256707	0.753493651	#N/A	#N/A	#N/A	2.098	0.567000348	0.874043557
190	SLC22A14	2.067	0.089289922	0.656043042	2.688	0.769536331	0.933346202	2.891	0.85738794	0.95864732
191	SLC22A15	8.009	0.084513866	2.181123319	#N/A	#N/A	#N/A	8.847	0.381019589	1.227902682
192	SLC22A16	3.095	0.065296082	0.649994937	2.124	0.730714613	1.124319054	3.479	0.423688522	0.829281818
193	SLC22A17	12.35	0.005143608	0.165379455	12.72	0.0053053	0.099846422	10.95	0.079214572	0.662402269
194	SLC22A18	8.767	0.005632828	9.850029204	10.25	0.060056793	1.750505322	11.17	0.261714659	1.306110315
195	SLC22A20	5.16	0.118017614	1.513455657	4.302	0.125318725	1.645577012	5.761	0.854425611	1.04394127
196	SLC22A23	11.01	0.18613621	0.732236243	10.93	0.328172713	0.7949691	11.01	0.237749252	0.75747109
197	SLC22A24	#N/A	#N/A	#N/A	#N/A	#N/A	#N/A	0.02371	0.508250264	0.6243975
198	SLC22A25	1.485	0.560505808	1.244159479	#N/A	#N/A	#N/A	0.3611	0.553623138	0.845453997
199	SLC22A31	2.148	0.064005979	2.177809684	7.082	0.957247652	0.980034222	4.384	0.336349429	1.255904152
200	SLC22A32	13.8	0.151984632	0.520777031	12.84	0.230917145	1.323767095	12.64	0.080249031	1.515747368
201	SLC22B1	11.03	0.00645692	1.24456E-08	7.816	0.542593398	0.731669645	9.415	0.455545976	0.837719319
202	SLC22B2	6.865	0.184562171	0.687745903	6.044	0.773603428	0.89772672	8.395	0.708582936	0.915837266
203	SLC22B3	4.004	0.100387319	1.837563444	3.77	0.126883416	1.824201947	5.336	0.793868991	1.063348163
204	SLC22B4	10.27	0.00857364	0.110312324	6.645	0.274152307	0.773569553	6.655	0.274152307	0.773569553
205	SLC22B5	4.224	0.006386187	1.877519827	3.628	0.034510927	1.646670801	3.648	0.020991888	1.722191234
206	SLC23A1	7.018	0.225678874	0.65080523	4.654	0.183913787	1.547693117	5.817	0.182580366	1.376081389
207	SLC23A2	11.11	0.000331584	0.438987144	11.01	0.070103146	0.631897823	11.27	0.053816684	0.635553014
208	SLC23A3	8.075	0.157150225	0.658084169	6.229	0.90627828	0.971657549	6.932	0.584137671	0.879443305

209	SLC24A1	9.5	0.034081576	1.763965527	8.956	0.182324258	1.768442869	9.644	0.688539425	1.099478974
210	SLC24A2	4.336	0.041343364	2.743406514	3.253	0.088235956	3.189470098	6.586	0.107941082	1.46113191
211	SLC24A3	11.15	0.070986538	0.44263007	9.929	0.378218274	1.259786102	10.24	0.822488436	0.948304559
212	SLC24A4	6.831	0.040897393	0.364170425	2.334	0.680145636	1.510890243	5.51	0.192887811	0.736088906
213	SLC24A5	1.555	0.038907019	0.393753202	#N/A	#N/A	#N/A	0.4699	0.041373122	0.546793623
214	SLC25A1	11.28	0.632374877	0.870466293	12.52	0.256300355	1.327100938	12.05	0.930520357	1.020668503
215	SLC25A2	0.877	0.503536287	0.83898905	#N/A	#N/A	#N/A	1.921	0.683412034	0.908701005
216	SLC25A3	14.61	0.036648608	1.796154909	14.46	0.945857325	1.018060461	14.23	0.805169312	1.060284321
217	SLC25A4	11.7	0.001872703	0.083618993	11.46	0.032487592	0.433815676	10.92	0.253862195	0.761064114
218	SLC25A5	13.19	0.045219136	5.912084748	12.95	0.067261812	27080456.31	14.01	0.535698385	1.155951252
219	SLC25A5P1	3.358	0.001762833	0.485430581	2.158	0.319844622	0.702454403	3.843	0.174547948	0.725942233
220	SLC25A6	14.89	0.086694539	0.663815383	14.72	0.699396498	0.912713713	14.87	0.409708768	0.823810693
221	SLC25A6P1	#N/A	#N/A	#N/A	#N/A	#N/A	#N/A	#N/A	#N/A	#N/A
222	SLC25A7	2.352	0.134660824	0.471095475	#N/A	#N/A	#N/A	0.9742	0.991133334	0.997396663
223	SLC25A8	12.11	0.004950878	0.518737917	12.27	0.100373824	0.667926331	12.14	0.05663674	0.639004669
224	SLC25A9	6.464	0.002999606	0.324725915	6.24	0.021449031	0.518872014	5.727	0.032002127	0.603287909
225	SLC25A10	11.84	0.007025975	2.146048625	11.04	0.37884825	1.228978149	10.78	0.498524547	1.173495105
226	SLC25A11	11.12	0.003153945	0.448601349	12.23	0.140085579	3.90945E-08	11.5	0.321355353	0.792323614
227	SLC25A12	11.18	0.221849364	0.493381593	9.487	0.530339104	1.379484699	10.44	0.479396357	1.182341172
228	SLC25A13	10.66	0.001586011	2.804265874	11.11	0.334663273	1.259264868	10.95	0.069244009	1.535265455
229	SLC25A14	9.584	0.000520447	1.12802E-08	10.03	0.014283288	1.24766E-08	9.092	0.006853092	0.521803452
230	SLC25A15	8.226	0.008146616	1.859421487	6.939	0.00562353	9.782401316	8.052	0.021075289	1.803867827
231	SLC25A15P1	#N/A	#N/A	#N/A	#N/A	#N/A	#N/A	#N/A	#N/A	#N/A
232	SLC25A16	10.14	0.023625328	0.575542868	10.62	0.755081295	0.894861211	10.15	0.03254568	0.59288473
233	SLC25A17	9.925	0.04329886	3.10058188	10.21	0.102210091	1.515610571	10.4	0.529508498	1.1585835
234	SLC25A18	4.584	0.085897804	0.547185639	4.279	0.192885689	0.550708854	5.749	0.930730347	0.979679702
235	SLC25A19	9.718	0.017701025	1.744591375	9.9	0.618989912	0.854771476	9.466	0.278737577	1.290107359
236	SLC25A20	11.07	0.211213743	0.485633047	10.29	0.901135672	1.029750634	10.3	0.762886622	1.073751316
237	SLC25A20P1	#N/A	#N/A	#N/A	#N/A	#N/A	#N/A	0.009083	0.757888746	8.23245E-07
238	SLC25A21	3.242	0.005096275	77996846.74	4.347	0.028203062	2.337174994	5.5	0.826872836	1.052688047

239	SLC25A22	11.14	0.02254017	1.699323381	11.08	0.130831848	1.426056	11.07	0.130831848	1.426056
240	SLC25A23	13.19	0.086298422	0.209383018	12.08	0.660252349	1.112729225	12.21	0.69657502	1.095837527
241	SLC25A24	11.25	6.47154E-05	5.357245979	10.59	0.000857272	7.784612575	11.64	0.331479138	1.260390462
242	SLC25A25	10.69	0.096732792	3.097694483	11.8	0.949341874	1.015445664	11.77	0.939768942	1.018271958
243	SLC25A26	10.04	0.5113034	0.847129407	9.49	0.067464458	5.215897031	10.34	0.526124665	1.159985215
244	SLC25A27	9.255	0.000104605	0.101840916	6.737	0.27819203	0.710642804	8.095	0.004901429	0.517537968
245	SLC25A28	10.59	0.121046605	0.691168573	11.46	0.567767225	1.191208452	10.93	0.237502315	0.756314696
246	SLC25A29	10.16	0.082524538	0.56998351	11.5	0.031072323	0.602477551	11.56	0.016900655	0.567592827
247	SLC25A30	9.77	0.008776443	2.077285504	9.352	0.016653169	2.679826682	9.989	0.185177968	1.379734241
248	SLC25A31	1.098	0.16539086	1.417318543	#N/A	#N/A	#N/A	0.5209	0.614077159	1.13269173
249	SLC25A32	10.51	0.023686416	1.747005351	#N/A	#N/A	#N/A	10.59	0.125068792	1.438056899
250	SLC25A33	8.782	0.217349467	1.548343938	10.44	0.571396987	0.569284858	9.227	0.378597272	0.812585501
251	SLC25A34	5.639	0.000350969	0.338381288	7.984	0.066852952	0.590989289	7.25	0.332857041	0.796599467
252	SLC25A35	8.135	0.125771721	0.677575962	6.969	1	NA	8.639	0.37656285	0.812389252
253	SLC25A36	11	0.062369002	3.503930883	11.04	0.145833238	2.30317912	11.78	0.543335607	1.154172275
254	SLC25A37	10.21	0.002479246	11.44591815	12.43	0.623599017	1.128870585	12.05	0.94311175	1.016888527
255	SLC25A38	10.89	0.147379147	0.708204142	11.14	0.79544713	0.940390253	11.11	0.764490629	0.931911771
256	SLC25A39	13.72	0.026528465	1.762898337	13.16	0.136107531	1.416593117	13.15	0.136107531	1.416593117
257	SLC25A40	9.761	0.005593493	2.732336871	9.596	0.010911733	3.454481485	10.11	0.263004574	1.308637364
258	SLC25A41	3.093	0.137040823	0.704105191	3.27	0.383711144	0.814898573	3.492	0.341741698	0.800361604
259	SLC25A42	9.547	0.01797147	0.49116382	9.857	0.101240409	0.503022894	9.318	0.414881117	0.826217251
260	SLC25A43	10.09	0.002340255	2.471931203	#N/A	#N/A	#N/A	10.36	0.00977823	1.863015208
261	SLC25A44	10.95	0.174166278	1.555107035	10.39	0.283286437	2.812854513	11.2	0.093221691	0.674208518
262	SLC25A45	9.022	0.009550084	0.529681925	10.06	0.605593325	0.737492223	9.028	0.012628934	0.5421128
263	SLC25A46	11.09	0.096838425	2.294042202	#N/A	#N/A	#N/A	11.57	0.321254402	1.262250904
264	SLC25A47	4.255	0.004986278	0.169157306	#N/A	#N/A	#N/A	2.17	0.352643985	0.804436429
265	SLC25A48	1.59	0.032206269	2.426808345	3.25	0.122084127	1.454867869	3.485	0.355278613	1.245548915
266	SLC25A49	13.45	0.533451848	0.862656108	12.67	0.325818563	2.594892941	13.61	0.758105348	1.074828294
267	SLC25A50	11.48	0.010144311	8.609227036	#N/A	#N/A	#N/A	12	0.178612377	1.37061828
268	SLC25A51	8.66	0.01954019	2.610202763	8.347	0.295382449	2.083174603	9.016	0.383170128	0.814780309

269	SLC25A51P1	#N/A	#N/A	#N/A	#N/A	#N/A	#N/A	0.0251	0.930817203	1.091645028
270	SLC25A51P2	#N/A	#N/A	#N/A	#N/A	#N/A	#N/A	#N/A	#N/A	#N/A
271	SLC25A51P3	#N/A	#N/A	#N/A	#N/A	#N/A	#N/A	0.004195	0.538009246	1.844523948
272	SLC25A52	0.7952	0.02351271	0.584273962	#N/A	#N/A	#N/A	0.8914	0.043719068	0.619058766
273	SLC25A53	7.212	0.008816495	0.545379943	8.437	0.094123912	0.383939906	7.453	0.132023183	0.694808021
274	SLC26A1	5.888	0.050991584	0.606367531	6.464	0.14083626	0.705244579	6.871	0.176177118	0.729104137
275	SLC26A2	10.96	0.03180316	1.654678419	9.448	0.831874367	1.133396477	10.71	0.62258754	1.124312313
276	SLC26A3	7.876	0.030048405	2.013213674	#N/A	#N/A	#N/A	3.101	0.948712063	0.984928724
277	SLC26A4	4.637	0.018734016	1.99202283	6.409	0.488980441	1.267698688	5.254	0.832158638	1.050893029
278	SLC26A5	5.535	0.09248156	0.384574377	3.086	0.74001532	1.080855073	3.12	0.572818359	1.141196184
279	SLC26A6	10.17	0.662367423	0.902482517	10.39	0.947355988	0.984501728	10.36	0.920349715	1.023798155
280	SLC26A7	6.779	0.002433504	0.151520484	4.843	0.734635828	0.921072558	5.285	0.673988952	0.906085397
281	SLC26A8	2.705	0.113642049	0.688730892	1.709	0.637529177	0.828333453	3.156	0.414156574	0.825673822
282	SLC26A9	4.377	0.006505051	9.642367112	6.253	0.109363935	1.971908663	10.15	0.371355742	0.811332983
283	SLC26A10	6.354	0.016926922	0.57527562	5.831	0.270665776	0.752306489	6.629	0.095329602	0.677464347
284	SLC26A11	10.29	4.49293E-05	0.051773499	10.57	0.001664322	0.082216825	9.652	0.005007652	0.511689683
285	SLC27A1	12.55	0.513385416	1.262230539	11.59	0.879193994	0.965014613	11.57	0.809162832	0.944988805
286	SLC27A2	10.09	0.305313077	1.499476113	7.566	0.741801475	1.088961148	8.17	0.448200173	0.836897687
287	SLC27A3	10.98	0.142293057	0.70530712	11.66	0.769530355	0.900917853	10.95	0.345614662	0.800405876
288	SLC27A4	11.74	0.007337943	1.860564491	11.07	0.375719067	1.31086275	11.53	0.056781364	1.562454306
289	SLC27A5	7.732	0.131831006	0.669977324	8.444	0.431532657	0.821473102	8.287	0.434197959	0.831157952
290	SLC27A6	1.935	0.083061948	1.975398379	3.418	0.428420766	1.215598956	3.834	0.870550531	0.962248314
291	SLC28A1	2.644	0.124601035	1.601362186	1.929	0.946289511	1.025645865	3.442	0.796080381	0.941092847
292	SLC28A2	1.305	0.131438642	2.817726211	4.737	0.547939691	0.86528115	4.553	0.51050809	0.854285164
293	SLC28A3	7.128	0.002321842	6.775801841	7.839	0.151913388	1.71439312	9.911	0.989374995	0.996811517
294	SLC29A1	12.04	0.017138232	2.503099806	11.17	0.154908953	3.789949875	12.59	0.222353466	1.33343564
295	SLC29A2	11.44	0.062519333	0.187530044	7.536	0.290649902	1.851561345	9.844	0.636279492	1.117964154
296	SLC29A3	9.158	0.029054442	2.649961302	8.656	0.01192548	79791879.53	9.914	0.18988889	1.362333641
297	SLC29A4	11.34	0.001636604	0.186327506	12	0.020790364	0.21634783	9.691	0.175886508	0.727698295
298	SLC30A1	11.5	0.002678944	2.452541488	10.41	0.017231508	78323815.13	11.76	0.036075657	1.634337537

299	SLC30A2	11.98	0.005197827	2.758097052	7.858	0.437504838	1.20376408	7.891	0.437504838	1.20376408
300	SLC30A3	4.238	0.08458459	0.509844635	2.198	0.506185127	1.233451323	3.244	0.522469591	1.163138836
301	SLC30A4	8.6	0.055122136	2.939990976	7.908	0.573597363	1.750033168	9.21	0.379694664	0.814211148
302	SLC30A5	11.73	0.032357367	1.650173859	10.88	0.405345708	1.626329558	11.7	0.044165673	1.605499088
303	SLC30A6	10.82	0.002737988	2.51575781	#N/A	#N/A	#N/A	11	0.013401422	1.831761934
304	SLC30A7	10.96	0.000245201	6.767503053	10.78	0.001202324	7.471535817	11.42	0.614694842	1.127121062
305	SLC30A8	2.948	0.033388254	6.451621468	8.57	0.830709237	1.058772927	9.936	0.837124455	0.951703885
306	SLC30A9	11.7	0.016521938	2.901750965	12.32	0.092258962	1.531990275	12.08	0.570071628	1.142171676
307	SLC30A10	3.101	0.045019777	1.910617604	3.006	0.15696012	1.585743006	4.516	0.779566492	0.936369087
308	SLC31A1	10.82	0.001916098	87293795.41	10.47	0.017107706	78328623.87	11.49	0.212205823	1.341836576
309	SLC31A1P1	1.843	0.401308439	0.612594957	#N/A	#N/A	#N/A	0.5498	0.849516986	0.953771171
310	SLC31A2	9.691	0.01177278	1.908389017	8.861	0.015598185	2.445774329	9.756	0.050396941	1.618652465
311	SLC32A1	1.325	0.063900525	0.607119372	#N/A	#N/A	#N/A	0.9179	0.157031331	0.70825354
312	SLC33A1	11.06	0.004832514	2.530417348	11.5	0.148176482	1.417837924	11.38	0.043321052	1.601038896
313	SLC33A2	8.786	0.065211323	0.572635771	9.842	0.933697965	1.020417313	10.06	0.669763243	1.105857315
314	SLC34A1	0.7796	0.010643634	2.038830067	#N/A	#N/A	#N/A	1.503	0.316569544	1.26886908
315	SLC34A2	6.25	0.002488492	11.23728779	8.948	0.295402176	1.356527063	10.03	0.339546947	1.269690136
316	SLC34A3	2.293	0.68151327	0.902618769	2.885	0.490787488	1.179263235	3.09	0.965128358	0.98978513
317	SLC35A1	9.892	0.006480178	9.533474192	10.95	0.089743732	1.487923127	10.93	0.129732859	1.426796619
318	SLC35A2	11.47	0.00046638	2.56064271	11.62	0.000578473	2.289933343	11.65	0.001349881	2.155808849
319	SLC35A3	10.44	0.000214425	5.520398854	9.823	0.00048999	14.75253984	11.09	0.046157623	1.610831373
320	SLC35A4	11.92	0.01761061	4.693447406	12.23	0.050274133	1.883783258	12.51	0.555187287	1.148566415
321	SLC35A5	10.5	0.002070605	3.80067894	10.55	0.008431717	2.59926849	10.86	0.025041714	1.723767433
322	SLC35B1	12.39	0.130785827	0.42099895	11.73	0.73835186	0.922522472	11.62	0.931869551	0.980022029
323	SLC35B2	11.79	0.005667686	5.802171965	11.69	0.005667686	5.802171965	12.35	0.022038912	1.717304841
324	SLC35B3	9.988	0.001911007	85420347.49	9.999	0.005830718	9.726414225	10.69	0.453382816	1.192742546
325	SLC35B4	9.751	0.057755291	1.952910775	10.25	0.295010935	1.287915925	10.23	0.204915432	1.360673195
326	SLC35C1	11.02	0.006127643	5.734093569	11.18	0.016076677	3.242485228	12	0.425407421	1.213184235
327	SLC35C2	12.46	0.154730912	1.429058511	11.98	0.81481053	1.059021411	12.12	0.889592135	1.033141614
328	SLC35D1	11.35	0.046502331	1.645022103	#N/A	#N/A	#N/A	10.82	0.205615781	1.351649923

329	SLC35D2	10.81	0.020647649	2.100547696	#N/A	#N/A	#N/A	11.2	0.678792879	0.907434477
330	SLC35D3	6.231	0.099294494	1.497925041	2.961	0.373421151	1.458855887	5.171	0.348495433	1.247125516
331	SLC35D4	8.286	0.000388263	6.273091655	8.885	0.070912917	1.525961227	8.93	0.085983308	1.493289718
332	SLC35E1	12.23	0.004839394	2.025862237	12.72	0.061006148	1.690756694	12.29	0.020015188	1.72939933
333	SLC35E2	7.709	0.010097199	0.440365839	7.198	0.495382624	0.850311883	7.3	0.572795067	0.875860711
334	SLC35E3	9.707	0.424963902	0.829357298	10.69	0.937342317	0.945139729	9.832	0.791200072	0.939568958
335	SLC35E4	7.952	0.037234184	2.547091797	9.043	0.110489291	1.455385138	9.045	0.072257555	1.525374023
336	SLC35F1	7.044	0.013605188	0.202877049	7.671	0.144225554	0.365789115	5.971	0.237576594	0.758302809
337	SLC35F2	9.741	0.000559809	6.030854356	9.119	0.00267612	6.599077273	10.71	0.051982401	1.602148806
338	SLC35F3	9.21	0.003296944	1.19183E-08	4.056	0.323347159	2.003437028	6.838	0.697688561	0.912026703
339	SLC35F4	5.866	0.028546851	0.147334288	#N/A	#N/A	#N/A	3.189	0.820626862	0.948130973
340	SLC35F5	10.99	0.00078155	5.818041016	11.19	0.006371763	2.694260204	11.66	0.485519709	1.181038172
341	SLC35F6	11.35	0.0242762	3.017802408	11.27	0.181214972	2.162475948	11.89	0.353945651	1.24291643
342	SLC35G1	7.754	0.00712153	2.190647037	9.235	0.488140561	0.610965131	8.126	0.208389956	1.345399758
343	SLC35G2	8.951	0.090987486	0.669166081	7.116	0.42404173	1.597357187	8.845	0.395471023	0.818179229
344	SLC35G3	1.497	0.151905792	0.439393421	#N/A	#N/A	#N/A	0.273	0.807708108	0.923392465
345	SLC35G4	#N/A	#N/A	#N/A	#N/A	#N/A	#N/A	0.02621	0.000289008	6.757172856
346	SLC35G5	2.575	0.100672669	0.659721114	#N/A	#N/A	#N/A	2.226	0.192902476	0.733186866
347	SLC35G6	1.209	0.049005707	0.604133819	#N/A	#N/A	#N/A	0.872	0.314696139	0.789115837
348	SLC36A1	9.238	0.000675037	92304065.6	9.673	0.009052548	3.563427242	10.65	0.386714207	1.244786525
349	SLC36A2	0.8804	0.065017794	1.681914067	#N/A	#N/A	#N/A	0.3377	0.102026526	1.587660987
350	SLC36A3	1.184	0.04169115	0.165468248	#N/A	#N/A	#N/A	0.159	0.084778854	0.486889835
351	SLC36A4	9.438	0.151650073	0.707468872	#N/A	#N/A	#N/A	9.633	0.977121492	0.993290921
352	SLC37A1	12.64	0.04940185	0.331681269	9.735	0.484721059	3331991.911	11.54	0.926711292	0.978637719
353	SLC37A2	7.716	0.001180749	5.703327365	8.95	0.054290569	1.736122205	9.466	0.158729434	1.424493366
354	SLC37A3	10.99	0.000205315	9.067004706	10.79	0.004318469	10.26340886	11.44	0.01559556	1.793419059
355	SLC37A4	12.23	0.008507864	0.418488987	11.63	0.426198936	0.829705803	11.73	0.142420514	0.709205056
356	SLC38A1	11.84	0.003547752	4.805693817	10.97	0.018525277	77197188.85	12.71	0.194825416	1.360716539
357	SLC38A2	13.36	0.000915497	2.385316552	13.07	0.043648135	1.918101772	13.43	0.004654895	2.02687641
358	SLC38A3	4.135	0.475309259	0.80398291	7.085	0.936754533	1.018891058	7.427	0.838750789	0.953375273

359	SLC38A4	11.23	0.011436276	0.117891791	10.04	0.125883352	0.494631034	7.796	0.892616841	0.968779301
360	SLC38A5	7.338	0.005476353	9.957811097	8.941	0.258753756	1.451314316	10.9	0.796497354	1.062893131
361	SLC38A6	9.044	0.004764427	1.970540586	8.072	0.060739565	5.39549215	9.049	0.009388629	1.85271261
362	SLC38A7	10.19	0.003383255	3.588017842	10.22	0.00532132	2.899710385	10.58	0.155391279	1.396738449
363	SLC38A8	2.078	0.6157679	1.127708578	5.611	0.323430416	0.497433662	2.19	0.831747746	0.951379511
364	SLC38A9	9.186	0.016397584	2.107401076	8.818	0.050418656	2.653613243	9.385	0.481935589	1.179423805
365	SLC38A10	13.1	0.22380578	0.723703434	13.72	0.785825288	0.93784695	13.57	0.794805101	1.063152092
366	SLC38A11	8.625	0.003517917	0.437621111	10.03	0.122679801	0.413682721	7.87	0.102717923	0.683057856
367	SLC39A1	13.14	0.003728056	2.703155636	#N/A	#N/A	#N/A	13.46	0.069458616	1.548843705
368	SLC39A2	1.077	0.028254269	1.975899911	5.264	0.224075803	1.618631801	2.499	0.771611019	1.071109323
369	SLC39A3	9.831	0.012254168	0.490002828	10.05	0.406642096	0.798391491	10.56	0.49106842	1.177516033
370	SLC39A4	11.32	0.020036092	1.747773229	10.17	0.239573265	1.434143437	11.34	0.034203269	1.658337522
371	SLC39A5	11.88	0.155589013	1.652225971	7.034	0.782725643	1.07813378	8.746	0.626522227	1.121697802
372	SLC39A6	12.31	0.027225539	1.733944431	11.76	0.238148104	1.337102499	11.87	0.132782274	1.425762976
373	SLC39A7	14.27	0.012757526	2.112042343	14.02	0.852813233	1.051684637	13.73	0.42226252	1.206854684
374	SLC39A8	10.52	0.000236461	2.52776339	11.04	0.217971797	1.381201471	10.58	0.003612779	2.008497788
375	SLC39A9	12.21	0.044174061	2.465162219	11.92	0.092124025	3.142242161	12.57	0.110094258	1.453670024
376	SLC39A10	10.4	0.002134968	3.032417464	9.869	0.054138492	2.393293612	10.97	0.016629067	1.78035072
377	SLC39A11	11.3	0.003817477	2.829863325	10.18	0.020069846	76151549.68	11.79	0.164970692	1.385429973
378	SLC39A12	1.589	0.041425373	1.610872996	#N/A	#N/A	#N/A	1.234	0.175037779	1.391625988
379	SLC39A13	11.75	0.963612617	0.988871443	11.51	0.601976223	1.156434512	12.08	0.979992005	0.994107984
380	SLC39A14	11.88	0.002150086	85407932.66	11.88	0.002150086	85407932.66	13.25	0.101229487	1.481055599
381	SLC40A1	14.76	0.458263418	0.745001422	12.65	0.404487898	1.332099474	13.72	0.9641022	1.010681611
382	SLC41A1	11.19	0.300978361	1.30669202	10.84	0.451872738	1.279574599	11.28	0.991418766	1.002528184
383	SLC41A2	10.92	0.026071292	1.79754556	9.586	0.095509989	2.586566637	11.09	0.09492054	1.512147591
384	SLC41A3	11.32	0.061616764	2.324697725	#N/A	#N/A	#N/A	11.81	0.552396349	1.150757331
385	SLC42A1	2.1	0.046686449	1.656589532	#N/A	#N/A	#N/A	1.149	0.146491874	1.405032602
386	SLC42A2	4.705	0.030500566	3.61523E-08	2.355	0.753389513	0.929039548	2.372	0.780896706	0.936869834
387	SLC42A3	2.809	0.001319916	12.51815551	6.943	0.015602111	1.890869259	5.429	0.019437129	1.740622809
388	SLC43A1	10.33	0.048563316	0.625809788	11.64	0.400853975	1.330975898	10.4	0.459234459	0.837823842

389	SLC43A2	11.29	0.000104305	0.408410356	12.64	0.164530016	0.270351465	11.45	0.003631584	0.500229719
390	SLC43A3	11.52	0.016354858	1.84925763	10.56	0.012150906	3.445790189	11.54	0.047771631	1.642784297
391	SLC44A1	12.66	0.000543144	14.27036296	12.12	0.001822721	12.01388163	13.46	0.125296285	1.444475773
392	SLC44A2	14.09	0.001375535	2.09829384	12.59	0.008960274	5.425243462	13.8	0.005590882	1.989765199
393	SLC44A3	10.02	0.021502941	3.562823263	11.06	0.157621071	1.420558404	11.11	0.17164121	1.396275233
394	SLC44A4	11.21	0.011285882	8.47508881	10.94	0.020023255	7.429030777	13.64	0.970514611	1.009109623
395	SLC44A5	4.01	0.467068023	1.675849363	8.293	0.994398304	1.001682638	7.265	0.482215194	0.847943795
396	SLC45A1	8.788	0.002428891	0.198449854	6.108	0.544672905	0.700711781	7.836	0.06765338	0.647087901
397	SLC45A2	4.086	0.00048863	0.069059829	2.256	0.51065269	0.84849218	2.723	0.218329407	0.749086549
398	SLC45A3	8.949	0.000782667	90357379.83	#N/A	#N/A	#N/A	10.89	0.622303176	1.123363662
399	SLC45A4	10.73	0.040128731	2.342264282	11.4	0.370459332	1.240874917	11.57	0.467694409	1.185921171
400	SLC46A1	11.31	0.000203483	1.0183E-08	11.93	0.046216721	3.68088E-08	10.53	0.691422152	1.099259481
401	SLC46A2	4.29	0.522401453	1.171545295	1.894	0.364035493	1.405137557	3.504	0.948899579	1.015513066
402	SLC46A3	11.41	0.283082203	0.738382541	#N/A	#N/A	#N/A	11.01	0.753123791	1.078058607
403	SLC47A1	5.629	0.002012619	3.527342396	6.099	0.014705355	2.209074627	6.979	0.937485831	1.018792831
404	SLC47A2	3.583	0.100686959	0.561589274	1.754	0.369605887	0.786821458	2.555	0.055204226	0.638155876
405	SLC48A1	11.29	0.02432931	2.063590686	#N/A	#N/A	#N/A	10.66	0.34461197	0.800237281
406	SLC49A1	10.37	0.004120414	0.373839393	10.29	0.037996443	0.551328116	9.972	0.34696675	0.799725631
407	SLC49A2	8.098	0.01566858	2.554618297	7.097	0.023697079	3.524869971	8.815	0.291057187	1.294770043
408	SLC49A3	8.731	0.005803425	2.605809386	7.907	0.023969082	3.060470947	9.401	0.296657563	1.278270799
409	SLC49A4	9.775	1.65418E-05	8.538777228	9.616	4.98791E-05	19.08310544	10.31	0.021595634	1.762238722
410	SLC50A1	11.55	0.007201735	2.105613761	10.64	0.062915469	2.880728985	11.88	0.185537969	1.362896847
411	SLC51A	6.911	0.511100086	0.856440653	7.7	0.509238962	0.754932141	6.939	0.652633269	0.899294253
412	SLC51B	2.732	0.021574053	1.995122281	1.631	0.809396286	1.100793219	3.406	0.22321622	1.333818617
413	SLC52A1	3.038	0.001792895	6.965891256	3.259	0.003388777	4.075881504	4.692	0.779481994	0.936444072
414	SLC52A2	11.93	0.036068788	1.725217295	11.86	0.096419436	1.555747639	12.15	0.199441839	1.356565436
415	SLC52A3	8.388	0.001845999	5.236937178	7.34	0.001303011	90552408.74	9.683	0.046951438	1.625590999
416	SLC53A1	11.5	0.034074767	1.770060066	10.81	0.035852612	4.025776656	11.64	0.064758969	1.559805305
417	SLC54A1	10.22	0.134460189	0.689279799	11.65	0.355792957	1.09858E-07	10.51	0.785280354	0.938085397
418	SLC54A2	11.18	0.076270872	0.625651352	12.55	0.41185277	1.384266791	11.71	0.124133578	0.693364225

419	SLC54A3	1.003	0.682714451	1.190456312	#N/A	#N/A	#N/A	0.1104	0.637661305	1.205877744
420	SLC55A1	11.92	0.093354695	1.503849132	#N/A	#N/A	#N/A	11.65	0.593532983	1.133491065
421	SLC55A2	8.89	0.002164977	2.027114648	10.18	0.17907223	1.698260761	8.614	0.001847936	2.072339292
422	SLC55A3	11.4	0.007429616	0.534745642	12.14	0.906996152	0.963657899	11.7	0.837359154	0.952855118
423	SLC56A1	10.95	0.423031486	0.817123541	11.02	0.790919569	1.066987762	11.26	0.685233187	1.100108473
424	SLC56A2	10.22	0.005110548	2.754309045	7.394	0.069963926	26371097.98	9.067	0.364095927	0.80846401
425	SLC56A3	11.56	0.000577926	14.18351075	11.09	0.00415535	83789802.92	12.59	0.106900455	1.465467839
426	SLC56A4	10.18	0.463495557	0.838123341	11.54	0.77370021	0.813653314	10.14	0.841065773	0.95354685
427	SLC56A5	9.684	6.52278E-06	0.355317276	11.01	0.023277747	3.52641E-08	9.817	0.000951082	0.455048946
428	SLC57A1	10.61	0.002123196	2.852497122	10.1	0.05774863	2.910708653	11.02	0.537142249	1.155903559
429	SLC57A2	11.08	0.00168145	83451967.15	10.84	0.003009766	83188851.94	11.79	0.074232579	1.521422007
430	SLC57A3	7.827	0.000838995	3.832512849	8.213	0.021637323	2.050458036	8.636	0.016157367	1.787510671
431	SLC57A4	11.06	0.154235821	1.508789448	#N/A	#N/A	#N/A	11.26	0.897784514	1.031105995
432	SLC57A5	11.05	0.032362563	1.992647095	10.47	0.041630924	3.893223602	11.35	0.935393488	1.019214681
433	SLC57A6	7.921	0.190182974	0.470200841	5.361	0.65000891	1.113166982	5.276	0.831160771	1.051525045
434	SLC58A1	12.22	0.000115434	17.27369215	13.26	0.168243205	1.628425445	12.7	0.030218393	1.67114529
435	SLC58A2	13.04	0.022809666	0.139945106	9.128	0.550495662	1.530070587	11.61	0.244908195	0.760615745
436	SLC59A1	7.519	0.08694704	3.201243484	#N/A	#N/A	#N/A	9.569	0.758872097	1.075287769
437	SLC59A2	3.186	0.00349334	4.782798748	4.277	0.0914083	1.491497862	4.335	0.142100521	1.41068075
438	SLC60A1	10.99	0.027636025	1.711085544	10.68	0.103150408	1.476971981	10.12	0.638511193	1.116291583
439	SLC61A1	10.66	0.001731373	5.256775875	11.29	0.049287773	1.611338651	11.32	0.133034099	1.42510315
440	SLC62A1	12.51	0.459798471	1.226959496	10.96	1	NA	12.85	0.672267938	0.905284708
441	SLC63A1	10.71	0.071953457	2.463282746	11.19	0.276044568	1.354336959	11.64	0.536669082	1.156401211
442	SLC63A2	13.47	0.276953876	1.536223421	10.02	0.144492861	1.956039752	11.65	0.740397062	1.082410776
443	SLC63A3	7.048	0.238408159	1.335023972	5.463	0.155007558	2.056790912	7.072	0.336399982	1.263571321
444	SLC64A1	11.73	0.001740105	88691616.57	11.23	0.005092675	83919468.34	12.53	0.758009216	1.075112105
445	SLC65A1	11.25	0.001869365	3.492281514	12.46	0.543328075	1.176374594	11.89	0.028117972	1.669544355
446	SLC65A2	12.81	0.226060545	1.612261277	9.494	0.553104408	1.198886113	10.61	0.524959264	0.856515473

*Abbreviations: HR – Hazard Ratio, p – p-value, cutoff – optimal threshold value for grouping, outcome – overall survival, dis – disease-specific survival, mean – mean expression-based grouping.
